# Fractional Sobolev spaces on Riemannian manifolds

**DOI:** 10.1007/s00208-024-02894-w

**Published:** 2024-06-03

**Authors:** Michele Caselli, Enric Florit-Simon, Joaquim Serra

**Affiliations:** 1 Scuola Normale Superiore, Piazza dei Cavalieri 7, 56126 Pisa, Italy; 2 Department of Mathematics, ETH Zürich, Rämistrasse 101, 8092 Zürich, Switzerland

## Abstract

This article studies the canonical Hilbert energy $H^{s/2}(M)$ on a Riemannian manifold for $s\in (0,2)$, with particular focus on the case of closed manifolds. Several equivalent definitions for this energy and the fractional Laplacian on a manifold are given, and they are shown to be identical up to explicit multiplicative constants. Moreover, the precise behavior of the kernel associated with the singular integral definition of the fractional Laplacian is obtained through an in-depth study of the heat kernel on a Riemannian manifold. Furthermore, a monotonicity formula for stationary points of functionals of the type ${\mathcal {E}}(v)=[v]^2_{H^{s/2}(M)}+\int _M F(v) \, dV$, with $F\ge 0$, is given, which includes in particular the case of nonlocal *s*-minimal surfaces. Finally, we prove some estimates for the Caffarelli–Silvestre extension problem, which are of general interest. This work is motivated by Caselli et al. (Yau’s conjecture for nonlocal minimal surfaces, arxiv preprint, 2023), which defines nonlocal minimal surfaces on closed Riemannian manifolds and shows the existence of infinitely many of them for any metric on the manifold, ultimately proving the nonlocal version of a conjecture of Yau (Ann Math Stud 102:669–706, 1982). Indeed, the definitions and results in the present work serve as an essential technical toolbox for the results in Caselli et al. (Yau’s conjecture for nonlocal minimal surfaces, arxiv preprint, 2023).

## Introduction

In recent years we have seen a great development of the theory of nonlocal equations. The simplest example of a nonlocal operator is the fractional Laplacian, $(-\Delta )^{\sigma }u=\int _{{\mathbb {R}}^n} (u(x)-u(y))\frac{1}{|x-y|^{n+2\sigma }}$, where $\sigma \in (0,1)$ and $u:{\mathbb {R}}^n\rightarrow {\mathbb {R}}$. Formally, it corresponds to the $\sigma$-th power of the usual Laplacian, and it is, therefore, an operator of order (of differentiation) $2\sigma$. Another way to look at it is as the operator arising from the Euler–Lagrange equation of the functional

$$\begin{aligned} {\mathcal {E}}(v)=[v]^2_{H^\sigma ({\mathbb {R}}^n)}=\int \!\int \limits _{{\mathbb {R}}^n\times {\mathbb {R}}^n} (u(x)-u(y))^2\frac{1}{|x-y|^{n+2\sigma }}, \end{aligned}$$

which involves a fractional Sobolev energy term. There are precise multiplicative constants one should put in front of these objects and which will be given later, but we will omit them in the introduction for the sake of exposition.

The present work addresses how the fractional Sobolev energy $H^{\sigma }(M)=W^{\sigma ,2}(M)$ and the associated fractional Laplacian on *M* have a natural, canonical interpretation in the case where *M* is a closed Riemannian manifold. We give several definitions for these objects and show them to be identical, which justifies their canonical nature. Moreover, we obtain fundamental properties for these objects thanks to a deeper study of their different definitions. We give a brief account in what follows:

Let $(M^n, g)$ be an *n*-dimensional, closed Riemannian manifold, with $n\ge 2$. Let us start by giving the definition of the fractional Sobolev seminorm $H^{s/2}(M)$ —for convenience and consistency with the notation in our paper [12], throughout the paper we put $\sigma = s/2$, where $s\in (0,2)$.

The $H^{s/2}(M)$ seminorm can be defined in at least three equivalent ways:

(i) Using the *heat kernel*^1^$H_M(t,p,q)$ of *M*, we can put
  1 $$\begin{aligned} K_s(p,q):= \int \limits _{0}^\infty H_M(p,q,t)\,\frac{dt}{t^{1+s/2}}. \end{aligned}$$
  We then define
  2 $$\begin{aligned}{}[u]^2_{H^{s/2}(M)}:= \int \!\int \limits _{M\times M}(u(p)-u(q))^2 K_s(p,q) \,dV_p \,d V_q. \end{aligned}$$
(ii) Following a *spectral approach*, we can set
  3 $$\begin{aligned}{}[u]^2_{H^{s/2}(M)} = \sum _{k\ge 1} \lambda _k^{s/2} \langle u,\phi _k \rangle ^2_{L^2(M)} \end{aligned}$$
  where $\{\phi _k\}_k$ is an orthonormal basis of eigenfunctions of the Laplace–Beltrami operator $(-\Delta _g)$ and $\{\lambda _k\}_k$ are the corresponding eigenvalues. For $s=2$ this immediately recovers the usual $[u]^2_{H^{1}(M)}$ seminorm.
(iii) Considering a *Caffarelli–Silvestre type extension* (cf. [3, 9]), namely, a degenerate-harmonic extension problem in one extra dimension, we can set
  4 $$\begin{aligned}{}[u]^2_{H^{s/2}(M)} = \inf \left\{ \int \limits _{M\times {\mathbb {R}}_+} z^{1-s} |\widetilde{\nabla } U(p,z)|^2 \, dV_p dz \hspace{7pt}{\text{ s.t. }} \hspace{7pt}U(x, 0)=u(x) \right\} . \end{aligned}$$
  Here ${{\widetilde{\nabla }}}$ denotes the Riemannian gradient of the manifold ${\widetilde{M}} = M\times (0, \infty )$, with respect natural product metric ${\widetilde{g}}= g + dz\otimes dz$, and the infimum is taken over all *U* belonging to the weighted Hilbert space $\widetilde{H}^1(\widetilde{M})$ (see Definition 2.24 for the precise definition of this space, and we refer to Sect. 2.3 in general for all the basic properties of this extension characterization).

We will prove that (i)–(iii) define the same norm (not merely equivalent norms) up to explicit multiplicative constants. We emphasize that this gives a canonical definition of the $H^{s/2}(M)$ seminorm on a closed manifold.

Definition (i) through the expression (2) will allow us to control precisely the behavior of the fractional Sobolev energy. See, for example, Lemmas 3.10 and 3.11, which show that the fractional Sobolev energy is smooth with quantitative bounds under inner variations. For that, we will give precise quantitative estimates for the kernel $K_s(p,q)$ (defined in (1)) and its derivatives, depending only on local quantities. In particular, we will show that it is comparable to $\frac{1}{d(p,q)^{n+s}}$ if *p* and *q* are contained in a Riemannian ball with controlled geometry. We recall that the two kernels coincide in the case $M={\mathbb {R}}^n$ (up to a constant factor).

The estimates for $K_s$ will follow from corresponding estimates for the heat kernel $H_M$. Albeit of somewhat “standard flavor”, they are hard to find in the literature with this level of precision, and we give an almost completely self-contained account that we believe to be of independent interest.

The extension definition (iii) will be used to give a monotonicity formula for stationary points *u* of semilinear elliptic functionals, that is, of functionals of the form

$$\begin{aligned} {\mathcal {E}}(v)=[v]^2_{H^{s/2}(M)}+\int \limits _M F(v) \, dV, \end{aligned}$$

under the assumption that $F\ge 0$. More precisely, *u* need only be stationary for ${\mathcal {E}}(v)$ under inner variations; in particular, setting $F\equiv 0$ will give a monotonicity formula for nonlocal *s*-minimal surfaces, which we will define in a moment. Up to now, the result was known on ${\mathbb {R}}^n$ by [7, 8] and [20].

### Definition 1.1

Given $s \in (0, 1)$ and a (measurable) set $E \subset M$, we define the *s*-*perimeter of* *E* as

5 $$\begin{aligned} \text {Per}_s(E): = [\chi _E]^2_{H^{{s/2}}(M)}= \frac{1}{4}[\chi _E-\chi _{E^c}]^2_{H^{{s/2}}(M)}, \end{aligned}$$

where $\chi _E$ is the characteristic function of *E* and $E^c: = M\setminus E$.

From the estimates that we will prove for $K_s$, one can see that for every set $E\subset M$ with smooth boundary, one has that $(1-s)\text {Per}_s(E) \rightarrow \text {Per}(E)$ as $s\uparrow 1$ (up to a multiplicative dimensional constant, see [4] and also [1, 11, 15] for further details on the computation in the case of ${\mathbb {R}}^n$).

### Definition 1.2

The boundary $\partial E$ of a set $E\subset M$ is said to be an *s*-*minimal surface* if $\text {Per}_s(E)<\infty$ and, for every smooth vector field *X* on *M*, we have

$$\begin{aligned} \frac{d}{dt}\left| _{t=0} \text {Per}_s(\psi ^t_X(E)) = 0\right. \,, \end{aligned}$$

where $\psi ^t_X: M \times {\mathbb {R}}\rightarrow M$ denotes the flow of *X* at time *t*.

### Remark 1.3

As we prove^2^ in Lemma 3.10 and Lemma 3.11, if $\text {Per}_s(E,\Omega )<\infty$ and $X\in {\mathfrak {X}}_c({\mathcal {U}})$ is such that $\textrm{spt}(X) \subset {{\overline{\Omega }}}$ then the map $t\mapsto \text {Per}_s(\psi ^t_X(E), \Omega )$ is well-defined for all *t* and of class $C^{\infty }$. Thus, the previous definitions are meaningful.

In other words, $\partial E$ is an *s*-minimal surface if $u=\chi _E-\chi _{E^c}$ is stationary under inner variations for ${\mathcal {E}}(v)=[v]^2_{H^{s/2}(M)}$. The general monotonicity formula claimed before is the following (see Sect. 3.3 for the precise definitions and notation):

### Theorem 1.4

(Monotonicity formula) Let $(M^n, g)$ be an *n*-dimensional, closed Riemannian manifold. Let $s \in (0,2)$ and

$$\begin{aligned} {\mathcal {E}}(v)=[v]^2_{H^{s/2}(M)}+\int \limits _M F(v) \, dV, \end{aligned}$$

where *F* is any smooth nonnegative function. Let $u:M\rightarrow {\mathbb {R}}$ be stationary for ${\mathcal {E}}$ under inner variations, meaning that ${\mathcal {E}}(u)<\infty$ and for any smooth vector field *X* on *M* there holds $\frac{d}{dt}\big |_{t=0} {\mathcal {E}}(u\circ \psi _X^t)=0$, where $\psi _X^t$ is the flow of *X* at time *t*. For $(p_\circ ,0) \in \widetilde{M}$ and $R>0$ define

$$\begin{aligned} \Phi (R):= \frac{1}{R^{n-s}} \left( \beta _s \int \limits _{\widetilde{B}^+_R(p_\circ ,0)} z^{1-s}| \nabla U(p,z)|^2 \, dV_p dz + \int \limits _{B_R(p_\circ )} F(u) \, dV \right) , \end{aligned}$$

where *U* is the unique solution (see Theorem 2.25) in ${\widetilde{H}}^1({\widetilde{M}})$ to

$$\begin{aligned} {\left\{ \begin{array}{ll} \widetilde{ \textrm{div}}(z^{1-s} {{\widetilde{\nabla }}} U) = 0 &{} \text{ in } \hspace{7pt}\widetilde{M} , \\ U(p,0) = u(p) &{} \text{ for } \hspace{7pt}p \in \partial \widetilde{M} = M. \end{array}\right. } \end{aligned}$$

Then, there exists a positive constant *C* with the following property: whenever $R_\circ \le \text {inj}_{M}(p_\circ )/4$ and *K* is an upper bound for all the sectional curvatures of *M* in $B_{R_\circ }(p_\circ )$, then

$$\begin{aligned} R \mapsto \Phi (R)e^{C \sqrt{K} R } \hspace{7pt}\textit{is nondecreasing for} \hspace{7pt}R < R_\circ , \end{aligned}$$

and the inequality

$$\begin{aligned} \Phi '(R)&\ge - C \sqrt{K} \Phi (R)+\frac{ s }{R^{n-s+1}} \int \limits _{B_R(p_\circ )} F(u) \, dV\\&\quad + \frac{2\beta _s}{R^{n-s}} \int \limits _{\partial ^+ \widetilde{B}_R^+(p_\circ ,0)} z^{1-s} \langle \nabla U , \nabla d \rangle ^2 \, d\,{\widetilde{\sigma }} \end{aligned}$$

holds for all $$R < R_0 $$, with $$d(\cdot ) = d_{\widetilde{g}}((p_\circ ,0), \,\cdot \, )$$ the distance function on $$\widetilde{M}$$ from the point $$(p_\circ , 0)$$.

Moreover, in the particular case where $M={\mathbb {R}}^n$, $F\equiv 0$, $s\in (0,1)$, and $u=\chi _E-\chi _{E^c}$ where *E* is an *s*-minimal surface, there holds

$$\begin{aligned} \Phi '(R) = \frac{2\beta _{s}}{R^{n-s}} \int \limits _{\partial ^+ \widetilde{{\mathcal {B}}}_R^+(p_\circ ,0)} z^{1-s} \langle \nabla U , \nabla d \rangle ^2 \, dxdz \ge 0, \end{aligned}$$

which shows that $\Phi$ is nondecreasing and that it is constant if and only if *E* is a cone.

### Overview of the kernel estimates

Here is an overview of the estimates for the heat kernel $H_M$ and the singular kernel $K_s$ (defined in (1)) that will be proved in Sect. 2.2. In particular, the reader is advised to consult Theorem 2.13, which records several of the main results for $K_s$ including an explicit asymptotic expansion for short distances.

**Table:** 

	Heat kernel $H_M$	Singular kernel $K_s$
Global comparability on $({\mathbb {R}}^n,g)$	Lemma 2.14	Lemma 2.14
Short distance comparability	Lemma 2.15, Lemma 2.18	Lemma 2.20
Long distance estimates	Lemma 2.16, Lemma 2.17	Theorem 2.13, Proposition 2.21
Precise asymptotics	Proposition 2.19	Theorem 2.13

## The fractional Laplacian on a closed manifold (*M*, *g*)

Throughout the paper (unless otherwise stated) (*M*, *g*) will be a closed (i.e. compact and without boundary) Riemannian manifold of dimension *n*.

Taking inspiration from the case of ${\mathbb {R}}^n$, in this section we give several equivalent definitions for the fractional Laplacian $(-\Delta )^{s/2}$ on a closed Riemannian manifold, with $s \in (0,2)$.

### Spectral and singular integral definitions

The fractional Laplacian $(-\Delta )^{s/2}$ can be define as the *s*/2-th power (in the sense of spectral theory) of the usual Laplace–Beltrami operator on a Riemannian manifold, through Bochner’s subordination.

Given $\lambda >0$ and $s \in (0,2)$, the following numerical formula holds:

6 $$\begin{aligned} \lambda ^{s/2} = \frac{1}{\Gamma (-s/2)} \int \limits _{0}^{\infty } (e^{-\lambda t}-1)\frac{dt}{t^{1+s/2}} \,. \end{aligned}$$

Formally applying the above relation to the operator $L=(-\Delta )$ in place of $\lambda$, one obtains the following definition for the fractional Laplacian.

#### Definition 2.1

(*Spectral definition*) Let $s\in (0,2)$. The fractional Laplacian $(-\Delta )^{s/2}$ is the operator that acts on smooth functions *u* by

7 $$\begin{aligned} (-\Delta )^{s/2} \, u = \frac{1}{\Gamma (-s/2)} \int \limits _{0}^{\infty } (e^{t\Delta }u-u)\frac{dt}{t^{1+s/2}}\,. \end{aligned}$$

Here, the expression $e^{t\Delta }u$ is to be understood as the solution of the heat equation on *M* at time *t* and with initial datum *u*.

From now on, to denote the solution of the heat equation with initial datum *u*, we will write $P_t u$ in place of $e^{t\Delta } u$.

#### Remark 2.2

On a closed Riemannian manifold a closely related definition of the fractional Laplacian is available: if $\{\phi _k\}_{k=1}^{\infty }$ is an $L^2(M)$ orthonormal basis of eigenfunctions for $(-\Delta )$ with eigenvalues

$$\begin{aligned} 0< \lambda _1 < \lambda _2 \le \dotsc \le \lambda _k \xrightarrow {\,k \rightarrow \infty \,} +\infty \end{aligned}$$

and *u* is a smooth function then

$$\begin{aligned} (-\Delta )^{s/2} u = \sum _{k=1}^\infty \lambda _k^{s/2} \langle u, \phi _k\rangle _{L^2(M)} \phi _k . \end{aligned}$$

Since the solution to the heat equation on *M* with initial datum an eigenfunction $\phi _k$ is given by $e^{t\Delta }\phi _k = e^{-\lambda _k t}\phi _k$, the above definition is easily shown to be identical to (7) by first observing that they coincide for eigenfunctions (thanks to (6)), and then extending the result by approximation. In [21] (see also [10]) all the details of this equivalence are carried out in the case of certain positive second order operators with discrete spectrum on a domain $\Omega \subset {\mathbb {R}}^n$. In our case of $(-\Delta )$ on a closed Riemannian manifold, the proof is then completely analogous. Nevertheless, this characterization will not be used in what follows and is given only as complementary information.

The second definition for the fractional Laplacian, closely related to the spectral one, expresses it as a singular integral. It will be our working definition in a substantial portion of the article.

#### Definition 2.3

(*Singular integral definition*) The fractional Laplacian $(-\Delta )^{s/2}$ of order (of differentiation) $s \in (0,2)$ is the operator that acts on a regular function *u* by

8 $$\begin{aligned} (-\Delta )^{s/2} u(p)&= p.v. \int \limits _{M} (u(p)-u(q))K_s(p,q)\, dV_q\\&:=\lim _{\varepsilon \rightarrow 0}\int \limits _{M} (u(p)-u(q))K_s^{\varepsilon }(p,q) \, dV_q. \nonumber \end{aligned}$$

Here $K_s(p,q): M \times M \rightarrow {\mathbb {R}}$ denotes the singular kernel given by^3^

9 $$\begin{aligned} K_s(p,q)=\frac{s/2}{\Gamma (1-s/2)} \int \limits _{0}^{\infty } H_M(p,q,t) \frac{dt}{t^{1+s/2}} \end{aligned}$$

where $H_M:M\times M \times (0,\infty )\rightarrow {\mathbb {R}}$ denotes the usual heat kernel on *M*, and $K_s^{\varepsilon }(p,q)$ is the natural regularization

10 $$\begin{aligned} K_s^{\varepsilon }(p,q)=\frac{s/2}{\Gamma (1-s/2)} \int \limits _{0}^{\infty } H_M(p,q,t) e^{-\varepsilon ^2/4t} \frac{dt}{t^{1+s/2}}. \end{aligned}$$

#### Remark 2.4

If the compact manifold *M* is replaced by the Euclidean space ${\mathbb {R}}^n$ then

$$\begin{aligned} K_{s}(x,y){} & {} =\frac{s/2}{\Gamma (1-s/2)} \int \limits _{0}^{\infty } H_{{\mathbb {R}}^n}(x,y,t) \frac{dt}{t^{1+s/2}} \\ {}{} & {} = \frac{s/2}{\Gamma (1-s/2)}\int \limits _{0}^{\infty } \left( \frac{1}{(4\pi t)^{\frac{n}{2}}} e^{-\frac{|x-y|^2}{4t}}\right) \frac{dt}{t^{1+s/2}} = \frac{\alpha _{n,s}}{|x-y|^{n+s}} , \end{aligned}$$

where

11 $$\begin{aligned} \alpha _{n,s}= \frac{2^s \Gamma \Big (\tfrac{n+s}{2}\Big )}{\pi ^{ n/2} |\Gamma (-s/2)| } = \frac{s 2^{s-1} \Gamma \Big (\tfrac{n+s}{2}\Big )}{\pi ^{n/2}\Gamma (1-s/2)}. \end{aligned}$$

Hence we recover the usual form of the fractional Laplacian on ${\mathbb {R}}^n$.

Let us briefly comment on our choice of the “natural” regularization $K_s^{\varepsilon }(p,q)$ that we have used to define the principal value (*p*.*v*.) in (8). First, we will see in the proof of (44) that this approximation naturally appears in the computation. This is because $K_s^{\varepsilon }(p,q)$ is directly related to the fractional Poisson kernel ${\mathbb {P}}_{{\widetilde{M}}}(p,q,z)$ of ${\widetilde{M}}:= M\times (0, +\infty )$ by the formula

$$\begin{aligned} K_s^{\varepsilon }(p,q) = {\mathbb {P}}_{{\widetilde{M}}}(p,q,\varepsilon )\varepsilon ^{-s} , \end{aligned}$$

and the fractional Poisson kernel is the fundamental solution of the Caffarelli–Silvestre extension problem.

Moreover, if the compact manifold *M* is replaced by the Euclidean space ${\mathbb {R}}^n$ then

$$\begin{aligned} K_s^{\varepsilon }(x,y) = \frac{s/2}{\Gamma (1-s/2)} \int \limits _{0}^{\infty } H_{{\mathbb {R}}^n}(x,y,t) e^{-\varepsilon ^2/4t}\frac{dt}{t^{1+s/2}} = \frac{\alpha _{n,s}}{(|x-y|^2+\varepsilon ^2)^{\frac{n+s}{2}}} , \end{aligned}$$

which is arguably a very natural regularization of $\frac{\alpha _{n,s}}{|x-y|^{n+s}}$, and is easily seen to give the same notion of principal value that one would get by integrating $K_s(x,y)$ against a function on ${\mathbb {R}}^n\setminus B_{\varepsilon }(y)$ and then taking $\varepsilon \rightarrow 0^+$. This is also true on a Riemannian manifold, and actually many other desingularizations of the (singular) kernel $K_s(p,q)$ are possible and give the same notion of principal value (*p*.*v*.) under mild hypotheses:

#### Proposition 2.5

Let (*M*, *g*) be a closed, *n*-dimensional Riemannian manifold, and let $\{K_s^\varepsilon \}_{\varepsilon >0}$ be a family of nonnegative kernels defined on $L^\infty (M)$. Assume that the following hold:

- The $K_s^\varepsilon$ converge uniformly to $K_s(p,\cdot )$ on compact subsets of $M\setminus \{p\}$, as $\varepsilon \rightarrow 0^+$.
- There exist some $r=r(p)>0$ and some chart parametrization $\varphi :{\mathcal {B}}_{r}\subset {\mathbb {R}}^n\rightarrow M$ with $\varphi (0)=p$ such that:
  (i) The flatness assumptions $\textrm{FA}_2(M,g, r,p,\varphi )$ are satisfied (see Definition 2.9).
  (ii) Setting ${\widetilde{K}}_s^\varepsilon (y):=K_s^\varepsilon (p,\varphi (y))$, there is some positive constant *C* such that, for all $y\in {\mathcal {B}}_{r_p}$,
    12 $$\begin{aligned} {\widetilde{K}}_s^\varepsilon (y)\le \frac{C}{|y|^{n+s}} \end{aligned}$$
    and moreover the symmetry condition
    13 $$\begin{aligned} \Big |{\widetilde{K}}_s^\varepsilon (y)-{\widetilde{K}}_s^\varepsilon (-y)\Big |\le \frac{C}{|y|^{n+s-1}} \end{aligned}$$
    is satisfied.

Then, for every $f\in C^\infty (M)$, the limit $\lim _{\varepsilon \rightarrow 0}\int _M (f(p)-f(q)) K_s^\varepsilon (p,q) \, dV_q$ exists and is independent of the family $K_s^\varepsilon$. In particular, any such family gives the same value for (8) as the choice in (10).

#### Remark 2.6

As discussed above, this covers the case of removing a geodesic ball $B_{\varepsilon }(p)$ in the corresponding definition of the fractional Laplacian as an integral and then sending $\varepsilon$ to 0, as in the usual Euclidean definition of a principal value integral. Indeed, this corresponds to considering $K_s^\varepsilon :=K_s(p,q)\chi _{M\setminus B_{\varepsilon }(p)}$ in the Proposition above. Another reasonable desingularization could be

$$\begin{aligned} K_s^{\varepsilon }(p,q)=\frac{s/2}{\Gamma (1-s/2)} \int \limits _{\varepsilon }^{\infty } H_M(p,q,t) \frac{dt}{t^{1+s/2}} . \end{aligned}$$

The fact that both of these choices for the families $\{K_s^\varepsilon \}_\varepsilon$, as well as the choice (10), satisfy the hypotheses in Proposition 2.5, can be easily seen using the results that will appear in the next section. More precisely, they follow from the combination of Remark 2.10 and (the proof of) estimate (18) from Theorem 2.13. The latter shows that conditions (12) and (13) hold directly for the kernel $K_s$ thanks to precise estimates on the heat kernel $H_M$, and it is then simple to see that they hold for the regularisations $K_s^{\varepsilon }$ as well.

#### Proof of Proposition 2.5

Recall that $\varphi ^{-1}(p)=0$. Given $0<\delta <r_p$, we can write

$$\begin{aligned} \int \limits _M (f(p)-f(q))K_s^\varepsilon (p,q)\, dV_q&= \int \limits _{M\setminus \varphi ({\mathcal {B}}_\delta )} (f(p)-f(q))K_s^\varepsilon (p,q)\, dV_q\\ {}&\quad +\int \limits _{\varphi ({\mathcal {B}}_\delta )} (f(p)-f(q))K_s^\varepsilon (p,q)\, dV_q. \end{aligned}$$

By the dominated convergence theorem, the first term on the RHS converges to

$$\begin{aligned} \int \limits _{M\setminus \varphi ({\mathcal {B}}_\delta )} (f(p)-f(q))K_s(p,q)\, dV_q . \end{aligned}$$

Therefore

$$\begin{aligned}&\limsup _{\varepsilon \rightarrow 0} \left| \,\int \limits _M (f(p)-f(q)) K_s^\varepsilon (p,q)\, dV_q-\int \limits _{M\setminus \varphi ({\mathcal {B}}_\delta )} (f(p)-f(q))K_s(p,q)\, dV_q\right| \\&\quad \le \limsup _{\varepsilon \rightarrow 0}\left| \,\int \limits _{\varphi ({\mathcal {B}}_\delta )} (f(p)-f(q))K_s^\varepsilon (p,q)\, dV_q\right| . \end{aligned}$$

In other words, to conclude our desired result, it suffices to show that $\int _{\varphi ({\mathcal {B}}_\delta )} (f(p)-f(q))K_s^\varepsilon (p,q)\, dV_q$ is bounded independently of $\varepsilon$ and moreover can be made arbitrarily small by choosing $\delta$ small enough.

To check this, we start by changing variables using the coordinates given by $\varphi$, leading to

$$\begin{aligned} \int \limits _{\varphi ({\mathcal {B}}_\delta )} (f(p)-f(q))K_s^\varepsilon (p,q)\, dV_q&=\int \limits _{{\mathcal {B}}_\delta } (f(\varphi (0))-f(\varphi (y))) {\widetilde{K}}_s^\varepsilon (y)\sqrt{|g|}(y)\,dy. \end{aligned}$$

Defining $h(y):=(f(\varphi (0))-f(\varphi (y)))\sqrt{|g|}(y)$, which verifies that $h(y)=y\cdot \nabla h(0)+O(|y|^2)$, and using the symmetry of the Lebesgue measure under the transformation $y\mapsto (-y)$, we can then compute

$$\begin{aligned}&\left| \,\int \limits _{\varphi ({\mathcal {B}}_\delta )} (f(p)-f(q))K_s^\varepsilon (p,q)\, dV_q \right| =\left| \,\int \limits _{{\mathcal {B}}_{\delta }} h(y) {\widetilde{K}}_s^\varepsilon (y)\,dy\right| \\&\quad \le \left| \, \int \limits _{{\mathcal {B}}_{\delta }} y\cdot \nabla h(0) {\widetilde{K}}_s^\varepsilon (y)\,dy \right| +C\int \limits _{{\mathcal {B}}_{\delta }} |y|^2 {\widetilde{K}}_s^\varepsilon (y)\,dy\\&\quad = \left| \, \frac{1}{2} \int \limits _{{\mathcal {B}}_{\delta }} y\cdot \nabla h(0) {\widetilde{K}}_s^\varepsilon (y)\,dy+\frac{1}{2} \int \limits _{{\mathcal {B}}_{\delta }} (-y)\cdot \nabla h(0) {\widetilde{K}}_s^\varepsilon (-y)\,dy \right| \\&\qquad +C\int \limits _{{\mathcal {B}}_{\delta }} |y|^2 {\widetilde{K}}_s^\varepsilon (y)\,dy\\&\quad =\frac{1}{2} \left| \,\int \limits _{{\mathcal {B}}_{\delta }} y\cdot \nabla h(0) ({\widetilde{K}}_s^\varepsilon (y)-{\widetilde{K}}_s^\varepsilon (-y))\,dy\right| +C\int \limits _{{\mathcal {B}}_{\delta }} |y|^2 {\widetilde{K}}_s^\varepsilon (y)\,dy \end{aligned}$$

Using the assumptions on the kernel, we conclude that

$$\begin{aligned}&\left| \, \int \limits _{\varphi ({\mathcal {B}}_\delta )} (f(p)-f(q))K_s^\varepsilon (p,q)\, dV_q \right| \le C\int \limits _{{\mathcal {B}}_{\delta }} |y\cdot \nabla h(0)| \frac{1}{|y|^{n+s-1}}\,dy\\ {}&\quad \quad +C\int \limits _{{\mathcal {B}}_{\delta }} |y|^2\frac{1}{|y|^{n+s}}\,dy\\&\quad \le C\int _{{\mathcal {B}}_{\delta }} \frac{1}{|y|^{n+s-2}}\,dy\\&\quad \le C\delta ^{2-s}. \end{aligned}$$

Since $s\in (0,2)$, this quantity can be made arbitrarily small by choosing $\delta$ small enough, independently of $\varepsilon$. This concludes the proof of our result. $\quad \square$

We now show the equivalence between the spectral and singular integral definitions for the fractional Laplacian.

#### Proposition 2.7

For every $s\in (0,2)$ definitions (7) and (8) coincide, meaning that:

(i): For $u \in C^\infty (M)$ they coincide pointwise everywhere.
(ii): For $u\in L^2(M)$ they coincide as distributions (i.e. in duality with $C^\infty (M)$).

#### Proof

Let $u\in C^{\infty }(M)$. Expressing the solution $P_t u$ to the heat equation in terms of the initial datum as

$$\begin{aligned} P_t u(p)=\int \limits _M u(q) H_M(p,q,t) \,dV_q , \end{aligned}$$

and using that $\int _M H_M(p,q,t) \,dV_q=1$ gives, for every $\varepsilon >0$, that

14 $$\begin{aligned} \frac{1}{\Gamma (-s/2)} \int \limits _{0}^{\infty } (P_t u-u)\frac{e^{-\varepsilon ^2/4t} dt}{t^{1+s/2}} = \int \limits _M (u-u(q))K_s^\varepsilon (p,q) \, dV_q . \end{aligned}$$

Since *u* is smooth, letting $\varepsilon \rightarrow 0^+$ gives convergence of both integrals pointwise everywhere and

$$\begin{aligned} \frac{1}{\Gamma (-s/2)} \int \limits _{0}^{\infty } (P_t u-u)\frac{dt}{t^{1+s/2}} = p.v. \int \limits _M (u-u(q))K_s(p,q) \, dV_q , \end{aligned}$$

and this proves (*i*).

Now to show (*ii*) take $u\in L^2(M)$ and $\varphi \in C^\infty (M)$. Multiply (14) by $\varphi$ and integrate over *M* to get

15 $$\begin{aligned}{} & {} \frac{1}{\Gamma (-s/2)} \int \limits _M \int \limits _{0}^{\infty } (P_t u-u) \varphi \frac{e^{-\varepsilon ^2/4t}}{t^{1+s/2}} \, dt dV \nonumber \\{} & {} \quad = \int \!\int \limits _{M\times M} (u(p)-u(q))\varphi (p) K_s^\varepsilon (p,q) \, dV_q dV_p . \end{aligned}$$

Note that since $\varepsilon >0$ is fixed and positive, neither of the two integrals above is singular, and they are both absolutely convergent. Hence, we can exchange the other of integration in both integrals. For the left-hand-side using that $P_t$ is self adjoint in $L^2(M)$ we get

$$\begin{aligned}&\frac{1}{\Gamma (-s/2)} \int \limits _M \int \limits _{0}^{\infty } (P_t u-u) \varphi \frac{e^{-\varepsilon ^2/4t}}{t^{1+s/2}} \, dt dV = \frac{1}{\Gamma (-s/2)} \int \limits _0^\infty \frac{e^{-\varepsilon ^2/4t}}{t^{1+s/2}} \langle P_t u-u , \varphi \rangle _{L^2} \, dt \\&\quad = \frac{1}{\Gamma (-s/2)} \int \limits _0^\infty \frac{e^{-\varepsilon ^2/4t}}{t^{1+s/2}} \langle P_t \varphi -\varphi , u \rangle _{L^2} \, dt \\&\quad = \int _M \left( \frac{1}{\Gamma (-s/2)} \int \limits _{0}^{\infty } (P_t \varphi - \varphi ) \frac{e^{-\varepsilon ^2/4t}}{t^{1+s/2}} \, dt \right) u \, dV \,. \end{aligned}$$

Regarding the right-hand side, since $K_s^\varepsilon (p,q)$ is symmetric

$$\begin{aligned}{} & {} \int \!\int \limits _{M\times M} (u(p)-u(q))\varphi (p) K_s^\varepsilon (p,q) \, dV_q dV_p\\{} & {} \quad = \int \!\int \limits _{M\times M} (\varphi (p)-\varphi (q))u(p) K_s^\varepsilon (p,q) \, dV_q dV_p . \end{aligned}$$

Thus

$$\begin{aligned}{} & {} \int \limits _M \left( \frac{1}{\Gamma (-s/2)} \int \limits _{0}^{\infty } (P_t \varphi - \varphi ) \frac{e^{-\varepsilon ^2/4t}}{t^{1+s/2}} \, dt \right) u \, dV\\{} & {} \quad = \int \limits _{M} \left( \int \limits _M (\varphi (p)-\varphi (q))K_s^\varepsilon (p,q) \, dV_q \right) u(p) dV_p , \end{aligned}$$

and letting $\varepsilon \rightarrow 0^+$ and using (*i*) proves (*ii*). $\square$

#### Remark 2.8

On a noncompact Riemannian manifold, the mass preservation property $\int _M H_M(p,q,t) \,dV_q=1$ could fail, leading to undesired phenomena such as the fractional Laplacian of a constant being different from zero. It is thus natural in the noncompact case to assume that *M* is stochastically complete, i.e. that $\int _M H_M(p,q,t) \,dV_q=1$ for every $t>0$.

### Properties of the kernel

This section gives important estimates on the singular kernel $K_s(p,q)$. In order to precisely quantify the dependence of the constants in the estimates on the geometry of the ambient manifold, the notion of “local flatness assumption” will be very useful. Let us introduce it below.

Here, as in the rest of the paper, $${\mathcal {B}}_R(0)$$ denotes the Euclidean ball of radius *R* centered at 0 of $${\mathbb {R}}^n$$, and $$B_R(p) $$ denotes the metric ball on *M* of radius *R* and center *p*.

#### Definition 2.9

(**Local flatness assumption**) Let $(M^n,g)$ be an *n*-dimensional Riemannian manifold and $p\in M$. For $R>0$, we say that (*M*, *g*) satisfies the $\ell$-th order *flatness assumption at scale Raround the point p*,*with parametrization* $\varphi$, abbreviated as $\textrm{FA}_\ell (M,g, R,p,\varphi )$, whenever there exists an open neighborhood *V* of *p* and a diffeomorphism

$$\begin{aligned} \varphi : {\mathcal {B}}_R(0) \rightarrow V, \quad \text{ with } \varphi (0)=p , \end{aligned}$$

such that, letting $g_{ij}=g\left( \varphi _*\left( \frac{\partial }{\partial x^i}\right) , \varphi _* \left( \frac{\partial }{\partial x^j} \right) \right)$ be the representation of the metric *g* in the coordinates $\varphi ^{-1}$, we have

16 $$\begin{aligned} \big ( 1-\tfrac{1}{100}\big ) |v|^2 \le g_{ij}(x)v^i v^j \le \big ( 1+\tfrac{1}{100}\big )|v|^2 \hspace{7pt}\hspace{7pt}\forall \, v\in {\mathbb {R}}^n \text{ and } \forall \, x\in {\mathcal {B}}_R(0) , \end{aligned}$$

and

17 $$\begin{aligned} R^{|\alpha |}\bigg |\frac{\partial ^{|\alpha |} g_{ij} (x)}{\partial x^{\alpha }}\bigg |\le \tfrac{1}{100} \hspace{7pt}\hspace{7pt}\forall \alpha \text{ multi-index } \text{ with } 1\le |\alpha |\le \ell , \text{ and } \forall x\in {\mathcal {B}}_R(0).\nonumber \\ \end{aligned}$$

#### Remark 2.10

Notice that for any smooth closed Riemannian manifold (*M*, *g*), given $\ell \ge 0$, there exists $R_0>0$ for which $\textrm{FA}_\ell (M,g,R_0,p,\varphi _p)$ is satisfied for all $p\in M$, where $\varphi _p$ can be chosen to be the restriction of the exponential map^4^(of *M*) at *p* to the (normal) ball ${\mathcal {B}}_{R_0}(0)\subset T_p M \cong {\mathbb {R}}^n$.

#### Remark 2.11

The notion above of local flatness is used in our results to stress the fact that, once the local geometry of the manifold is controlled in the sense of Definition 2.9, then our estimates are independent of *M*. Interestingly, this makes our estimates in the present article and in [12] of local nature, even though the equation we deal with is nonlocal.

#### Remark 2.12

The following useful scaling properties hold.

- Given $M = (M,g)$ and $r>0$, we can consider the “rescaled manifold” ${\widehat{M}} = (M, r^2g)$. When performing this rescaling, the new heat kernel $H_{\widehat{M}}$ satisfies
  $$\begin{aligned} H_{\widehat{M}}(p,q,t) = r^{-n} H_M(p,q,t/r^2) . \end{aligned}$$
  As a consequence, the “rescaled kernel” ${\widehat{K}}_s$ defining the *s*-perimeter on ${\widehat{M}}$ satisfies
  $$\begin{aligned} {\widehat{K}}_s(p,q) = r^{-(n+s)} K_s(p,q). \end{aligned}$$
- Concerning the flatness assumption, it is easy to show that $\textrm{FA}_\ell (M,g, R, p,\varphi ) \Rightarrow \textrm{FA}_\ell (M,g, R',p,\varphi )$ for all $R'<R$ and $\textrm{FA}_\ell (M,g, R, p,\varphi )\Leftrightarrow \textrm{FA}_\ell (M, r^2g, R/r, p,\varphi (r\,\cdot \,))$.
- Similarly, if $\textrm{FA}_\ell (M,g, R, p,\varphi )$ holds, and $q\in \varphi ({\mathcal {B}}_R(0))$ is such that ${\mathcal {B}}_\varrho (\varphi ^{-1}(q))\subset {\mathcal {B}}_R(0)$, then $\textrm{FA}_\ell (M,r^2g, \varrho /r, q,\varphi _{\varphi ^{-1}(q),r})$ holds, where $\varphi _{x,\,\rho }:= \varphi (x+\rho \, \cdot \,)$.

In all the sections, we will use the (standard) multi-index notation for derivatives. A multi-index $\alpha =(\alpha _1,\alpha _2,\dots , \alpha _n)$ will be an *n*-tuple of nonnegative integers (in other words $\alpha \in {\mathbb {N}}^n$). We define

$$\begin{aligned} |\alpha |: = \alpha _1+ \alpha _2 +\cdots +\alpha _n. \end{aligned}$$

For a function $f: {\mathbb {R}}^n \rightarrow {\mathbb {R}}$ is of class $C^\ell$ we shall use the notation

$$\begin{aligned} \frac{\partial ^{|\alpha |}}{\partial x^{\alpha }} f: = \frac{\partial ^{\alpha _1 + \alpha _2 + \cdots \alpha _n}f}{(\partial x^1)^{\alpha _1} (\partial x^2)^{\alpha _2}\cdots (\partial x^n)^{\alpha _n} }. \end{aligned}$$

For $\alpha =0$, we put $\frac{\partial ^{|0|}}{\partial x^{0}} f:=f$.

The next main theorem gives the precise behavior of the kernel around a point satisfying flatness assumptions, including an explicit approximation in coordinates.

#### Theorem 2.13

Let (*M*, *g*) be a Riemannian *n*-manifold, not necessarily closed, $s\in (0,2)$ and let $p\in M$. Assume $\mathrm{FA_\ell }(M,g,R,p, \varphi )$ holds and denote $K(x,y): = K_s(\varphi (x), \varphi (y))$.

Given $x\in {\mathcal {B}}_R(0)$, let *A*(*x*) denote the positive symmetric square root of the matrix $(g_{ij}(x))$—$g_{ij}$ being the metric in coordinates $\varphi ^{-1}$—and, for $x,z\in {\mathcal {B}}_{R/2}(0)$, define

$$\begin{aligned} k(x, z): = K(x,x+z) \quad \text{ and }\quad {\widehat{k}}(x,z):= k(x,z) - \frac{\alpha _{n,s}}{|A(x)z|^{n+s}}. \end{aligned}$$

Then

18 $$\begin{aligned} \big |{\widehat{k}}(x,z)\big | \le R^{-1}\frac{C(n,s)}{|z|^{n+s -1}}\quad \text{ for } \text{ all } x,z \in {\mathcal {B}}_{R/4}(0) , \end{aligned}$$

and, for every multi-indices $\alpha ,\beta$ with $|\alpha |+|\beta | \le \ell$, we have

19 $$\begin{aligned} \bigg |\frac{\partial ^{|\alpha |}}{\partial x^{\alpha }} \frac{\partial ^{|\beta |}}{\partial z^{\beta }} k(x,z)\bigg | \le \frac{C(n,s, \ell )}{|z|^{n+s+ |\beta |}}\quad \text{ for } \text{ all } x,z \in {\mathcal {B}}_{R/4}(0). \end{aligned}$$

The constants *C*(*n*, *s*) and *C*(*n*, *s*, *l*) stay bounded for *s* away from 0 and 2.

Moreover, for all $x\in {\mathcal {B}}_{R/4}(0)$ and for all $q\in M{\setminus } \varphi ({\mathcal {B}}_R(0))$ we have

20 $$\begin{aligned} \bigg |\frac{\partial ^{|\alpha |}}{\partial x^{\alpha }} K_s(\varphi (x),q)\bigg | \le \frac{C(n,\ell )}{R^{n+s}} , \end{aligned}$$

and

21 $$\begin{aligned} \int \limits _{M\setminus \varphi ({\mathcal {B}}_R(0))}\bigg |\frac{\partial ^{|\alpha |}}{\partial x^{\alpha }} K_s(\varphi (x),q)\bigg | dV_q \le \frac{C(n,\ell )}{R^{s}}, \end{aligned}$$

for every multi-index $\alpha$ with $|\alpha |\le \ell$.

#### Heat kernel estimates

To prove Theorem 2.13 we will need several preliminary lemmas studying the properties of the heat kernel of *M*.

The first result compares locally the heat kernel $H_M(p,q,t)$ or the singular kernel $K_s(p,q)$ on ${\mathbb {R}}^n$ endowed with a metric *g* with the standard ones on ${\mathbb {R}}^n$.

##### Lemma 2.14

Let *g* be a smooth metric on ${\mathbb {R}}^n$ such that $\frac{|v|^2}{4}\le g_{ij}(x)v^iv^j\le 4|v|^2$ and $|Dg_{ij}(x)|\le 1$ for all $x, v \in {\mathbb {R}}^n$. Denote $M:= ({\mathbb {R}}^n,g)$ and let $K_s$ be defined by (9). Then, there exist positive constants $c_i=c_i(n)$ for $1\le i \le 6$ such that

$$\begin{aligned} \frac{c_1}{t^{n/2}} e^{- \frac{|x-y|^2}{c_2 t} } \le H_M(x,y,t) \le \frac{c_3}{t^{n/2}} e^{- \frac{|x-y|^2}{c_4 t} }, \end{aligned}$$

and

$$\begin{aligned} c_5 \frac{\alpha _{n,s}}{|x-y|^{n+s}} \le K_s(x,y) \le c_6 \frac{\alpha _{n,s}}{|x-y|^{n+s}} , \end{aligned}$$

for all $$(x,y,t ) \in {\mathbb {R}}^n \times {\mathbb {R}}^n \times [0, \infty )$$.

##### Proof

The two-sided estimates for the heat kernel $H_M$ follow directly from the classical parabolic estimates of Aronson [2]. The second inequality follows by integrating the first one, from the definition (9) of $K_s(x,y)$. $\square$

The next lemma concerns the concentration of mass of the heat kernel.

##### Lemma 2.15

Let (*M*, *g*) be a Riemannian *n*-manifold, $p\in M$, and assume $\mathrm{FA_0}(M,g,1,p, \varphi )$ holds. Then

$$\begin{aligned} 1-C\exp (-c/t)\le \int \limits _{\varphi ({\mathcal {B}}_{1/2}(0))} H_M(p,q,t)dV_q \le 1, \hspace{7pt}\text{ for } \text{ all } t>0, \end{aligned}$$

with $C,c>0$ depending only on *n*.

##### Proof

Put $H(x,y,t): = H_M(\varphi (x), \varphi (y),t)$. Let $g_{ij}\in C^0({\mathcal {B}}_1(0))$ be the metric coefficients in the chart $\varphi ^{-1}$, choose $\xi \in C^0_c({\mathcal {B}}_1(0))$ such that $\chi _{{\mathcal {B}}_{3/4}(0)}\le \xi \le \chi _{{\mathcal {B}}_{1}(0)}$ and put $g'_{ij}: = g_{ij}\xi + \delta _{ij}(1-\xi )$. By assumption we have $\big | {\overline{g}}'_{ij}(x) v^iv^j -|v|^2 \big |\le \tfrac{1}{100} |v|^2$ for all $x,v\in {\mathbb {R}}^n$. Morevoer, $g'_{ij}\equiv g_{ij}$ inside ${\mathcal {B}}_{3/4}(0)$. Consider the complete Riemannian manifold $M':=({\mathbb {R}}^n, g')$ and let $H'(x,y,t)$ denote its associated heat kernel. Then, by Lemma 2.14 we have

22 $$\begin{aligned} \frac{c_1}{t^{n/2}} e^{-c_2|x-y|^2/t}\le H'(x,y,t) \le \frac{c_3}{t^{n/2}} e^{-c_4|x-y|^2/t}. \end{aligned}$$

Now, since $H'(0,x,t)$ is the heat kernel of the stochastically complete manifold $M'$ we have

23 $$\begin{aligned} \int \limits _M H'(0,x,t) \sqrt{|g'|(x)} dx =1\quad \text{ for } \text{ all } t>0. \end{aligned}$$

On the other hand, for every fixed $\tau >0$ set $h(\tau ):= \frac{c_3}{\tau ^{n/2}} e^{-c_4(1/4)^2/\tau }$. Using (22) we have that $u(x,t) = \big (H'(0,x,t)- h(\tau )\big )^+$, where $(\,\cdot \,)^+$ denotes the positive part, is a subsolution of

$$\begin{aligned} {\left\{ \begin{array}{ll} u_t \le \frac{1}{\sqrt{|g'|}} \frac{\partial }{\partial x^i} \bigg ( \sqrt{|g'|} (g')^{ij} \frac{\partial }{\partial x^j} u \bigg ) \quad &{}\text{ in } {\mathcal {B}}_{1/4}(0)\times (0,\tau ) ,\\ u = 0 &{}\text{ in } \partial {\mathcal {B}}_{1/4}(0)\times (0,\tau ) . \end{array}\right. } \end{aligned}$$

Since $g'\equiv g$ in ${\mathcal {B}}_{1/4}(0)$, it easily follows (using that both *H*(0, *x*, *t*) and $H'(0,x,t)$ have as initial condition a Dirac delta with respect to the same volume form $\sqrt{|g|} dx$) that $u\le H(0,x,t)$ for all $t\in (0,\tau )$. This gives, for all $t\in (0, \tau )$

$$\begin{aligned} \int \limits _{{\mathcal {B}}_{1/4}} \big (H'(0,x,t)- h(\tau )\big )^+ \sqrt{|g|} dx =\int \limits _{{\mathcal {B}}_{1/4}} u(x,t) \sqrt{|g|} dx\le \int \limits _{{\mathcal {B}}_{1/4}} H(0,x,t)\sqrt{|g|} dx \end{aligned}$$

On the other hand, using (23) and (22) we obtain that for all $t\in (0, \tau )$

$$\begin{aligned} \begin{aligned} 1 - C\exp (-c/\tau )&\le 1 - \int \limits _{{\mathbb {R}}^n \setminus {\mathcal {B}}_{3/4}(0)} \frac{c_3}{t^{n/2}} e^{-c_4|x|^2/t} \big (1+\tfrac{1}{100}\big )^{n/2} dx\\&\le 1 - \int \limits _{{\mathbb {R}}^n \setminus {\mathcal {B}}_{3/4}(0)} H'(0,x,t) \sqrt{|g'|} dx\\&= \int \limits _{{\mathcal {B}}_{3/4}(0)} H'(0,x,t)\sqrt{|g|} dx. \end{aligned} \end{aligned}$$

Finally, since also $h(\tau )\le C\exp (-c/\tau )$ (notice that we can “absorbe” $\tau ^{-n/2}$ in $Ce^{-c/\tau }$ chosing $c>0$ slightly smaller and a larger *C*), we obtain the desired estimate

$$\begin{aligned} 1-C\exp (-c/\tau )\le & {} \int \limits _{{\mathcal {B}}_{1/4}(0)} H(0,x,t)\sqrt{|g|} dx\\\le & {} \int \limits _{\varphi ({\mathcal {B}}_{1/2}(0))} H(p,q,t)dV_q,\quad \forall t\in (0,\tau ) , \end{aligned}$$

and for all $\tau >0$. The bound by above by 1 of the same quantity follows immediately using that *H* is a heat kernel, i.e. nonnegative and with total mass bounded by 1. $\square$

##### Lemma 2.16

Under the same assumptions as in Theorem 2.13, for all $q\in M\setminus \varphi ({\mathcal {B}}_1(0))$ we have

24 $$\begin{aligned} \bigg |\frac{\partial ^{|\alpha |}}{\partial x^{\alpha }} H_M(\varphi (x),q,t)\bigg | \le C \exp (-c/t), \hspace{7pt}\text{ for } (x,t)\in {\mathcal {B}}_{1/2}(0) \times [0,\infty ) \end{aligned}$$

and for every multi-index $\alpha$ with $|\alpha |\le \ell$, with $C,c>0$ depending only on *n* and $\ell$.

##### Proof

Notice that $u(x,t): = H_M(\varphi (x),q,t)$ satisfies $u_t =Lu$, in ${\mathcal {B}}_1(0)\times [0,\infty )$ and $u\equiv 0$ at $t=0$, where

25 $$\begin{aligned} Lu = \frac{1}{\sqrt{|g|}} \frac{\partial }{\partial x^i} \bigg ( \sqrt{|g|} g^{ij} \frac{\partial u }{\partial x^j} \bigg ) \end{aligned}$$

is the Laplace–Beltrami operator with metric *g*.

Let us show that

26 $$\begin{aligned} |u|\le C \exp (-c/t)\quad \text{ for } (x,t)\in {\mathcal {B}}_{3/4}(0) \times [0,\infty ), \end{aligned}$$

with $C,c>0$ dimensional constants. This follows from the following standard probabilistic consideration. Fix $x_\circ \in \varphi ({\mathcal {B}}_{3/4}(0))$. By continuity of sample paths, the probability that a Brownian motion started at $q \in M{\setminus } \varphi ({\mathcal {B}}_1(0))$ hits $\varphi ({\mathcal {B}}_{\delta }(x_\circ ))$ ($0<\delta \ll 1$) within time $\le t$ is less that the supremum among $q' \in \varphi (\partial {\mathcal {B}}_{8/9})$ of the probability that a Brownian motion started at a point $q'$ hits $\varphi ({\mathcal {B}}_{\delta }(x_\circ ))$ within time $\le t$. This gives

27 $$\begin{aligned} u(x_\circ ,t) \le \sup _{q' \in \varphi (\partial {\mathcal {B}}_{8/9})} H_M(\varphi (x_\circ ), q',t). \end{aligned}$$

Let us now use (27), Lemma 2.15, and the parabolic Harnack inequality as follows to show (26).

For fixed $q' \in \varphi (\partial {\mathcal {B}}_{8/9})$ set $v(x,t):= H_M(\varphi (x), q',t)$ and consider the rescaled ${\widetilde{v}}(x,t):= v(x_\circ +rx, t_\circ + r^2t)$ for $r \in (0, 1/10 )$. Then ${\widetilde{v}}\ge 0$ satisfies a (uniformly) parabolic equation in ${\mathcal {B}}_1(0)\times (0,1)$ with smooth coefficients (that only improve as *r* gets smaller). Thus, by the Harnack inequality for every $x \in {\mathcal {B}}_{1/2}(0)$ and $t \in (1/4, 1/2)$ we have ${\widetilde{v}}(x,t) \le C \inf _{{\mathcal {B}}_{1/2}(0)} {\widetilde{v}}(\, \cdot \,, 1) \le C {\widetilde{v}}(y,1)$ for all $y \in {\mathcal {B}}_{1/2}(0)$. Integrating

$$\begin{aligned} {\widetilde{v}}(0,t)&\le C \int \limits _{{\mathcal {B}}_{1/2}(0)} {\widetilde{v}}(y,1) dy = C \int \limits _{{\mathcal {B}}_{1/2}(0)} v(x_\circ +ry,t_\circ +r^2) dy\\&= C r^{-n} \int \limits _{{\mathcal {B}}_{r/2}(x_\circ )} v(z,t_\circ +r^2) dz , \end{aligned}$$

for some $C=C(n)>0$. Thus, for all $t \in (t_\circ +r^2/4, t_\circ +r^2/2)$

$$\begin{aligned} v(x_\circ ,t) \le C r^{-n} \int \limits _{{\mathcal {B}}_{r/2}(x_\circ )} v(z,t_\circ + r^2) dz . \end{aligned}$$

But $\varphi ({\mathcal {B}}_{r/2}(x_\circ )) \subset M\setminus B_{1/10}(q')$ for every $q' \in \varphi (\partial {\mathcal {B}}_{8/9}(0))$. Then by Lemma 2.15 we get

$$\begin{aligned} v(x_\circ ,t) \le C r^{-n} \int \limits _{ M\setminus \varphi ({\mathcal {B}}_{1/10}(q')) } H_M(z,q',t_\circ +r^2) dz \le Cr^{-n} \exp \left( -\frac{c}{t_\circ +r^2}\right) , \end{aligned}$$

where $C,c>0$ depend only on *n*. Now, for small times $t_\circ \le 1/100$ choosing $r^2 = t_\circ$ (together with the probabilistic argument above) gives the result, since one can absorb the term $r^{-n}=t_\circ ^{-n/2}$ in the exponential up to slightly decreasing the value of *c*. For non-small times $t_\circ > 1/100$, one can just take $r=1/10$ and obtain upper bound by a constant as desired. This concludes the proof of (26).

Now, similarly to above, for all $r\in (0,1/4)$ and $(x_\circ ,t_\circ )\in {\mathcal {B}}_{1/2}(0)\times (0,\infty )$ the rescaled function ${\overline{u}}(x,t) = u(x_\circ +rx,t_\circ + r^2t)$ satisfies a (uniformly) parabolic equation with smooth coefficients (since the bounds on every $C^k$ norm of the coefficients only improve as *r* gets smaller) and, from (26), we have $|{\overline{u}}|\le C \exp (-c/t)$ in ${\mathcal {B}}_{1}\times (0,1)$. Hence standard parabolic Schauder estimates give

$$\begin{aligned} \bigg |\frac{\partial ^{|\alpha |}}{\partial x^{\alpha }} \, {\overline{u}}\bigg | \le C \exp (-c/t), \hspace{7pt}\text{ for } (x,t)\in {\mathcal {B}}_{1/2}(0) \times [1/2,1) , \end{aligned}$$

for every multi-index $\alpha$ with $|\alpha |\le \ell$, with $C>0$ depending only on *n* and $\ell$ and $c>0$ as above.

After scaling back the estimate above we obtain, for all $r\in (0,1/4]$

$$\begin{aligned} \bigg |\frac{\partial ^{|\alpha |}}{\partial x^{\alpha }} u(x_\circ , t_\circ + t )\bigg | \le Cr^{-|\alpha |} \exp (-c/r^2), \hspace{7pt}\text{ for } (x_\circ ,t )\in {\mathcal {B}}_{1/2}(0) \times [r^2/2,r^2) . \end{aligned}$$

Then, for “non-small” times $t_\circ \ge 1/16$ we notice that (24) follows taking $r=1/4$. On the other hand, for small times $t_\circ \in (0,1/16)$ we obtain (24) taking $r^2=t_\circ$, bounding $r^{-|\alpha |}$ by $t_\circ ^{-\ell /2}$, and absorbing (chosing $c>0$ smaller and *C* larger) this negative power of $t_\circ$ in the exponential. $\square$

##### Lemma 2.17

Under the same assumptions as in Theorem 2.13, we have

28 $$\begin{aligned}{} & {} \int \limits _{M\setminus \varphi ({\mathcal {B}}_1(0))} \bigg |\frac{\partial ^{|\alpha |}}{\partial x^{\alpha }} H_M(\varphi (x),q,t)\bigg | dV_q\nonumber \\{} & {} \quad \le C \exp (-c/t), \hspace{7pt}\text{ for } (x,t)\in {\mathcal {B}}_{1/2}(0) \times [0,\infty ) \end{aligned}$$

and for every multi-index $\alpha$ with $|\alpha |\le \ell$, with $C,c>0$ depending only on *n* and $\ell$.

##### Proof

It is similar to the proof of Lemma 2.16. Let $\sigma : M{\setminus }\varphi ({\mathcal {B}}_1(0))\rightarrow \{+1,-1\}$ be any measurable function to be chosen. Consider

$$\begin{aligned}u(x,t): = \int \limits _{M\setminus \varphi ({\mathcal {B}}_1(0))}H_M(\varphi (x),q,t) \sigma (q)dV_q,\end{aligned}$$

By Lemma 2.15—since $H_M \ge 0$ and $\int _M H_M(p,q,t)dV_q\le 1$—we obtain

29 $$\begin{aligned} |u(x,t)|\le & {} \int \limits _{M\setminus \varphi ({\mathcal {B}}_{1/4}(0))} H(\varphi (x),q,t) dV_q\nonumber \\\le & {} C\exp (-c/t),\qquad \forall (x,t)\in {\mathcal {B}}_{3/4}(0)\times [0,\infty ). \end{aligned}$$

Notice that in this estimate, *C* and *c* are positive dimensional constants (and in particular, they do not depend on the choice of $\sigma$). Also, by the superposition principle *u* satisfies $u_t =Lu$, in ${\mathcal {B}}_1(0)\times [0,\infty )$ and $u\equiv 0$ at $t=0$, where *L* is as in (25).

Now proceeding exactly as in the proof of Lemma 2.16 we obtain that

$$\begin{aligned} \bigg |\frac{\partial ^{|\alpha |}}{\partial x^{\alpha }} u\bigg | \le C \exp (-c/t), \hspace{7pt}\text{ for } (x,t)\in {\mathcal {B}}_{1/2}(0) \times [0,\infty ) \end{aligned}$$

for $|\alpha |\le \ell$. Now, for any given $\alpha$, *x*, and *t*, we can choose $\sigma : M\setminus \varphi ({\mathcal {B}}_1(0))\rightarrow \{+1,-1\}$ so that

$$\begin{aligned} \frac{\partial ^{|\alpha |}}{\partial x^{\alpha }} u(x,t)= & {} \int _{M\setminus \varphi ({\mathcal {B}}_1(0))}\frac{\partial ^{|\alpha |}}{\partial x^{\alpha }}H_M(\varphi (x),q,t) \sigma (q)dV_q\\= & {} \int _{M\setminus \varphi ({\mathcal {B}}_1(0))}\bigg |\frac{\partial ^{|\alpha |}}{\partial x^{\alpha }}H_M(\varphi (x),q,t)\bigg | dV_q, \end{aligned}$$

and we are done. $\square$

##### Lemma 2.18

(Localization principle) Let (*M*, *g*) and $(M',g')$ be two Riemannian *n*-manifolds. Assume that both *M* and $M'$ satisfy the flatness assumptions $\mathrm{FA_\ell }(M,g,1,p, \varphi )$ and $\mathrm{FA_\ell }(M',g,1,p', \varphi ')$ respectively, and suppose that $g_{ij}\equiv g'_{ij}$ in ${\mathcal {B}}_1(0)$ in the coordinates induced by $\varphi ^{-1}$ and $(\varphi ')^{-1}$.

Then, letting $H(x,y,t): = H_M(\varphi (x),\varphi (y),t)$ and $H'(x,y,t): = H_{M'} (\varphi '(x),\varphi '(y),t)$, we have that the difference $(H-H')(x,y,t)$ is of class $C^\ell$ in ${\mathcal {B}}_{1/2}(0) \times {\mathcal {B}}_{1/2}(0)\times [0,\infty )$ and

$$\begin{aligned}{} & {} \bigg |\frac{\partial ^{|\alpha |}}{\partial x^{\alpha }} \frac{\partial ^{|\beta |}}{\partial y^{\beta }} (H-H')(x,y,t)\bigg |\\{} & {} \quad \le C \exp (-c/t)\quad \text{ for } (x,y,t)\in {\mathcal {B}}_{1/2}(0) \times {\mathcal {B}}_{1/2}(0)\times [0,\infty ), \end{aligned}$$

whenever $\alpha$ and $\beta$ are multi-indices satisfying $|\alpha |+|\beta | \le \ell$, with $C,c>0$ depending only on *n* and $\ell$.

##### Proof

Let us show that

30 $$\begin{aligned} |H-H'|\le C \exp (-c/t)\quad \text{ for } (x,y,t)\in {\mathcal {B}}_{3/4}(0) \times {\mathcal {B}}_{3/4}(0)\times [0,\infty ), \end{aligned}$$

with $C,c>0$ dimensional constants.

Indeed, fix $x_\circ \in {\mathcal {B}}_{3/4}(0)$ and let us show first that we have

31 $$\begin{aligned} \big |(H-H')(x_\circ ,y,t)\big | \le C\exp (-c/t) \quad \text{ for } \text{ all } y\in {\mathcal {B}}_{3/4}(0)\setminus {\mathcal {B}}_{1/8}(x_\circ ) \end{aligned}$$

Indeed, the $L^\infty$ estimate of Lemma 2.16—appropriately rescaled to have $\varphi (B_{1/8}(x_\circ ))$ instead of $\varphi ({\mathcal {B}}_1(0))$—gives

32 $$\begin{aligned} H_M(\varphi (x_\circ ), \varphi (y),t) \le C\exp (-c/t)\quad \text{ for } \text{ all } y\in {\mathcal {B}}_{3/4}(0)\setminus {\mathcal {B}}_{1/8}(x_\circ ), \end{aligned}$$

and the same estimate with $H_{M}$ replaced by $H_{M'}$. Hence (30) follows using $|H-H'|\le H+H'$.

Now observing that for all $x_\circ$ as above $u(y,t):=(H-H')(x_\circ , y,t)$ solves the heat equation $(\partial _t -L_y) u=0$ with zero intial condition, (30) easily follows from the maximum principle.

Finally, the estimate for the higher derivatives follows from standard parabolic estimates, noticing that $u(x,y,t):= (H-H')(x,y,t)$ solves

$$\begin{aligned} \partial _t u = \frac{1}{2} (L_{x,y} u)\end{aligned}$$

where

$$\begin{aligned} L_{x,y} u = \frac{1}{\sqrt{|g(x)|}} \frac{\partial }{\partial x^i} \bigg ( \sqrt{|g(x)|} g^{ij}(x) \frac{\partial }{\partial x^j} u \bigg )+\frac{1}{\sqrt{|g(y)|}} \frac{\partial }{\partial y^i} \bigg ( \sqrt{|g(y)|} g^{ij}(y) \frac{\partial }{\partial y^j} u\bigg ). \end{aligned}$$

is the sum of the Laplace–Beltrami operators with respect to the variables *x* and *y* (or, equivalently, the Laplace–Beltrami operator with respect to the product metric in ${\mathcal {B}}_1(0)\times {\mathcal {B}}_1(0)$). $\square$

##### Proposition 2.19

Assume that $M = ({\mathbb {R}}^n, g)$ with $g = (g_{ij}(x))$ satisfying

33 $$\begin{aligned} \tfrac{1}{2} \textrm{id} \le (g_{ij}) \le 2 \textrm{id} \qquad \text{ and }\qquad \bigg |\frac{\partial ^{|\alpha |}}{\partial x^{\alpha }} g_{ij}\bigg | \le 1\quad \text{ for } \text{ all } |\alpha |\le \ell , \end{aligned}$$

for some $\ell \ge 1$. Let *H*(*x*, *y*, *t*) be the heat kernel of *M*.

For $x\in {\mathbb {R}}^{n}$ let *A*(*x*) denote the (unique) positive definite symmetric square root of the matrix $g(x) = (g_{ij}(x))$, and define *h*(*z*, *x*, *t*) by the identity

$$\begin{aligned} H(x,y,t) = \frac{1}{t^{n/2}} \,h\bigg (\frac{A(x)(y-x)}{\sqrt{t}},x, t\bigg ). \end{aligned}$$

Define also:

$$\begin{aligned} h_\circ (x, z, t) = h_\circ (z):= \frac{1}{(4\pi )^{n/2}} e^{-|z|^2/4}\qquad \text{ and } \qquad {\widehat{h}}:= h-h_\circ . \end{aligned}$$

Then, there are positive dimensional *C* and *c* such that

$$\begin{aligned} |{\widehat{h}}| \le C \min (1,\sqrt{t}) e^{-c|z|^2} \quad \text{ for } \text{ all } (x,z,t)\in {\mathbb {R}}^n\times {\mathbb {R}}^n \times (0,\infty ). \end{aligned}$$

Morever, we have

$$\begin{aligned} \bigg |\frac{\partial ^{|\alpha |}}{\partial x^{\alpha }} \frac{\partial ^{|\beta |}}{\partial z^{\beta }}h\bigg |\le & {} C e^{-c |z|^2} \quad \text{ for } \text{ all } (x,z,t)\in {\mathbb {R}}^n \times {\mathbb {R}}^n\\{} & {} \quad \times (0,1) \text { and }\alpha , \beta \text { with }|\alpha |+|\beta |\le \ell , \end{aligned}$$

for positive constants *C* and *c* depending only on *n* and $\ell$.

##### Proof of Proposition 2.19

Notice first that since $H(x,y,t) = H(y,x,t)$ we have

$$\begin{aligned} H(x,y,t)= t^{-n/2} h\bigg (\frac{A(x)(y-x)}{\sqrt{t}},x, t\bigg )= t^{-n/2} h\bigg (\frac{A(y)(x-y)}{\sqrt{t}},y, t\bigg ).\end{aligned}$$

Let $L_xf:= \frac{1}{\sqrt{ |g|(x)}} \tfrac{\partial }{\partial x^i}\bigg (\sqrt{|g|(x)}g(x)^{ij} \tfrac{\partial }{\partial x^j}f\bigg )$ denote the Laplace–Beltrami operator (with respect to *x*). Direct computation shows:

$$\begin{aligned} \begin{aligned} LH&= t^{- \frac{n}{2} - 1} \left( \sqrt{t} \bigg (\frac{\partial _i \big (\sqrt{|g|}g^{ij}\big )}{\sqrt{|g|}} \bigg )(x) A^l_j(y) \frac{\partial }{\partial z^l } h(*) + g^{ij}(x) \big (A_i^k A_j^l\big )(y) \frac{\partial ^2}{\partial z^k\partial z^l } h(*)\right) , \\ \partial _t H&= t^{- \frac{n}{2} - 1} \bigg ( -\frac{n}{2} h(*) - \frac{1}{2} \frac{\partial }{\partial z^l } h(*) \frac{(A(y)(x-y))^l}{\sqrt{t}} + t\partial _t h(*)\bigg ), \end{aligned} \end{aligned}$$

where

$$\begin{aligned}(*) \text{ means } \text{ evaluated } \text{ at } \bigg ( \frac{A(y)(x-y)}{\sqrt{t}}, y, t\bigg ).\end{aligned}$$

This leads to the equation for $h = h(z,y,t)$, where we denote $\partial _i: = \frac{\partial }{\partial z^i}$ and $\partial _{ij}: = \frac{\partial ^2}{\partial z^i\partial z^j}$,

$$\begin{aligned} t\partial _t h = {\overline{L}} h: = a^{ij}(z,y,t) \partial _{ij} h + \big (\sqrt{t} b^i(z,y,t) + \tfrac{z^i}{2}\big ) \partial _i h + \frac{n}{2} h, \end{aligned}$$

where

$$\begin{aligned} a^{ij}(z,y,t):= g^{kl}\Big (y + \sqrt{t} z\Big ) \big (A_k^i A_l^j\big )(y) \end{aligned}$$

and

$$\begin{aligned} b^i(z,y,t):= \bigg (\frac{\partial _k \big (\sqrt{|g|}g^{kl}\big )}{\sqrt{|g|}} \bigg )\Big (y +\sqrt{t} z \Big )A^i_l(y); \end{aligned}$$

with initial condition:

$$\begin{aligned}h(z,y,0^+) = h_\circ (z) = (4\pi )^{-\frac{n}{2}} e^{-|z|^2/4}.\end{aligned}$$

(Notice that we defined *h* so that its initial condition is independent of *y*.)

We emphasize that, by the assumption (33), this equation is uniformly elliptic, and the derivatives of $a^{ij}$, $b^i$ up to order $\ell$ in the variables *z* and *y* are uniformly bounded for times $t\in (0,T_\circ )$ by constants depending only on the constants *n* and $T_\circ$.

Let us now compute an equation for ${\widehat{h}}= h - h_\circ$. Since

$$\begin{aligned}\delta ^{ij} \partial _{ij} h_\circ + \tfrac{z^i}{2} \partial _i h + \frac{n}{2} h_\circ =0, \end{aligned}$$

we obtain

$$\begin{aligned} \begin{aligned} t\partial _t {\widehat{h}} - {\overline{L}} {\widehat{h}}&= {\overline{L}} h_\circ = (a^{ij}-\delta ^{ij})\partial _{ij} h_\circ + \sqrt{t} b^i \partial _i h_\circ \\&= \big ((a^{ij}-\delta ^{ij}) (z_i z_j -\delta _{ij}) - \sqrt{t} b^i \tfrac{z^j}{2}\delta _{ij} \big ) h_\circ \,. \end{aligned} \end{aligned}$$

We emphasize that ${\widehat{h}}$ satisfies the initial condition

$$\begin{aligned} {\widehat{h}} (z,y, 0) \equiv 0 \end{aligned}$$

Notice that (since by definition *A*(*y*) is a square root of *g*(*y*)) we have, for all *y*

$$\begin{aligned} g^{kl}(y) \big (A_k^i A_l^j\big )(y) = \delta ^{ij} \end{aligned}$$

and hence, since $g^{kl}$ is smooth,

$$\begin{aligned} |a^{ij}(z,y,t) -\delta ^{ij}| \le C \sqrt{t} \end{aligned}$$

Hence, we have

34 $$\begin{aligned} |t\partial _t {\widehat{h}} - {\overline{L}} {\widehat{h}}| \le C (1+ |z|^2)\sqrt{t}\, h_\circ \end{aligned}$$

Let us now find some barrier allowing us to control ${\widehat{h}}$. We can use as barrier

$$\begin{aligned} b(z,t): = \sqrt{t} e^{ -(1/4 - \kappa )|z|^2 } \end{aligned}$$

Direct computation shows that, for $\sqrt{t}< \theta \kappa$ (so that $a^{ij}\delta _{ij}\ge n -C\theta \kappa$ and $|\delta ^{ij}-a^{ij}|z^k z^l\delta _{ik}\delta _{jl}\le C\theta \kappa |z|^2$)

$$\begin{aligned} \begin{aligned} t\partial _t b -{\overline{L}} b&= \bigg ( \frac{1}{2} - 4\left( \frac{1}{4} - \kappa \right) ^2 \, (a^{ij})z^k z^l\delta _{ik}\delta _{jl} + 2\left( \frac{1}{4} - \kappa \right) \,a^{ij}\delta _{ij}\\ {}&\quad + \left( \sqrt{t} b^i + \frac{z^i}{2}\right) 2\left( \frac{1}{4} - \kappa \right) z^j\delta _{ij} -\frac{n}{2} \bigg ) b \\&\ge \left( \frac{1}{2} + \left( \frac{1}{4} - \kappa \right) 4\kappa |z|^2 - C\theta \kappa |z|^2 - C\kappa - C\theta \kappa |z| \right) b \\&\ge \left( \frac{1}{4} + \frac{\kappa }{2}|z|^2\right) b \ge 0, \end{aligned} \end{aligned}$$

provided we chose $\theta >0$ and $\kappa >0$ sufficiently small.

Since clearly $b \ge \sqrt{t} \, h_\circ$ we obtain that *Cb* is a supersolution of (34) for $\sqrt{t}< \theta \kappa$. This shows that $|{\widehat{h}}|\le Cb$ for all *t* small enough.

Notice that the estimate $|{\widehat{h}}|\le Cb$ (fixing $\kappa >0$ and $\theta >0$ small dimensional) shows, in particular, that

35 $$\begin{aligned} |{\widehat{h}}(z,y,t)|\le C\sqrt{t}\exp (-c|z|^2) \end{aligned}$$

holds with $c>0$ dimensional for all “small” times $t\in (0,\theta ^2\kappa ^2)$. On the other hand, for “non-small” times $t\ge \theta ^2\kappa ^2$, the standard heat kernel estimate (22) for *H* (which holds with $c_i$ dimensional) immediately yields (35) with $\sqrt{t}$ replaced by 1.

In order to bound the derivatives of *h* with respect to *z* we notice we notice that, in logarithmic time $\tau =\log t$, the function $h(z,y, e^\tau )$ satisfies, for *y* fixed, a standard parabolic equation with smooth coefficients in the domain ${\mathbb {R}}^n \times (-\infty , 0)$. Then, thanks to (35), applying standard parabolic estimates in parabolic cylinders $\{|x-x_\circ |<2, |\tau -\tau _\circ |<2 \}$ we easily obtain the claimed bounds for all partial derivatives of *h* with respect to *z*.

In order to show the regularity in *y*, one can then differentiate the equation with respect to *y* as many times as needed (the coefficients depend in a very smooth way also in *y*) and notice that the initial condition will be zero (since $h_\circ$ is independent of *y*). By standard parabolic regularity arguments (e.g., using a Duhamel-type formula to represent the solutions), we obtain the estimates. $\square$

#### Estimates for the singular kernel $K_s(p,q)$

As a first consequence of Lemma 2.18 we have that the following “local version” of Lemma 2.14 above also holds.

##### Lemma 2.20

Let $s_0 \in (0,2)$ and $s \in (s_0,2)$. Let (*M*, *g*) be a Riemannian *n*-manifold and $p \in M$. Assume that $\textrm{FA}_1(M,g,p,1,\varphi )$ holds. Then

$$\begin{aligned} c_7 \frac{\alpha _{n,s}}{|x-y|^{n+s}} \le K_s(\varphi (x),\varphi (y)) \le c_8 \frac{\alpha _{n,s}}{|x-y|^{n+s}} , \end{aligned}$$

for all $x,y \in {\mathcal {B}}_{1/2}(0)$, where $c_7,c_8>0$ depends on *n* and $s_0$.

##### Proof

Take $\eta \in C^\infty _c({\mathcal {B}}_{1}(0))$ with $\chi _{{\mathcal {B}}_{1/2}(0)} \le \eta \le \chi _{{\mathcal {B}}_1(0)}$ and let $g'_{ij}:=g_{ij} \eta +(1-\eta )\delta _{ij}$. This is a metric on ${\mathbb {R}}^n$ with $g'_{ij}=g_{ij}$ in ${\mathcal {B}}_{1/2}(0)$. Denote by $K_s,K_s'$ and $H,H'$ the singular kernels and heat kernels of (*M*, *g*) and $({\mathbb {R}}^n, g')$ respectively. Then, by Lemma 2.18 applied to the manifolds (*M*, *g*) and $({\mathbb {R}}^n, g')$ we have, for $x,y \in {\mathcal {B}}_{1/4}(0)$:

$$\begin{aligned} \big | K_s(\varphi (x), \varphi (y))-K_s'(x,y) \big |&\le \frac{s/2}{\Gamma (1-s/2)} \int \limits _{0}^\infty \big | H(\varphi (x), \varphi (y),t)-H'(x,y,t) \big | \frac{dt}{t^{1+s/2}} \\ {}&\le \frac{C s}{\Gamma (1-s/2)} \int \limits _{0}^{\infty } e^{-c/t} \frac{dt}{t^{1+s/2}} \le C(2-s), \end{aligned}$$

for some dimensional $C=C(n)$. Then, the result follows directly by Lemma 2.14 (and the explicit formula (11) for $\alpha _{n,s}$) for $x,y \in {\mathcal {B}}_{1/4}(0)$, and the conclusion also holds for $x,y \in {\mathcal {B}}_{1/2}(0)$ by a standard covering argument. $\square$

Now, we have all the ingredients to give the proof of Theorem 2.13.

##### Proof of Theorem 2.13

Note that the statement is scaling invariant. Hence, with no loss of generality, we can (and do) assume that $R=1$. Moreover, it suffices to consider the case $M=({\mathbb {R}}^n,g)$, $p =0$, $\varphi = \textrm{id}$, and $g_{ij}$ satisfying the assumptions of Proposition 2.19:

Indeed, similarly to the proof of Corollary 2.20, in the general case we can fix a radially nonincreasing cutoff function $\eta \in C^\infty _c({\mathcal {B}}_1)$ such that $\eta \equiv 1$ in ${\mathcal {B}}_{7/8}$ and consider the “extended” metric $g'_{ij}: = g_{ij}\eta + \delta _{ij}(1-\eta )$. Observe that (*M*, *g*) and $({\mathbb {R}}^n,g')$ the assumptions of Lemma 2.18 with $M'={\mathbb {R}}^n$ and $\varphi '= \textrm{id}$. Let *H*(*x*, *y*, *t*) and $H'(x,y,t)$ be defined as in Lemma 2.18.

Recall that, by definition, for all $x,y\in {\mathcal {B}}_1(0)$

36 $$\begin{aligned} K(x,y)= & {} K_s(\varphi (x),\varphi (y)) = c_s\int \limits _0^\infty H_M(\varphi (x),\varphi (y),t) \frac{dt}{t^{1+s/2}}\nonumber \\= & {} c_s \int \limits _0^\infty H(x,y,t) \frac{dt}{t^{1+s/2}}, \end{aligned}$$

where $c_s =\frac{s/2}{\Gamma (1-s/2)}$. Let likewise

$$\begin{aligned} K'(x,y) = c_s \int \limits _0^\infty H'(x,y,t) \frac{dt}{t^{1+s/2}}. \end{aligned}$$

Now, thanks to Lemma 2.18 we obtain, for all $x,y\in {\mathcal {B}}_{1/2}$:

$$\begin{aligned} \bigg |\frac{\partial ^{|\alpha |}}{\partial x^{\alpha }} \frac{\partial ^{|\beta |}}{\partial y^{\beta }} (K-K')(x,y,t)\bigg |{} & {} \le c_s\int \limits _0^\infty \bigg |\frac{\partial ^{|\alpha |}}{\partial x^{\alpha }} \frac{\partial ^{|\beta |}}{\partial y^{\beta }} (H-H')(x,y,t)\bigg |\frac{dt}{t^{1+s/2}}\\ {}{} & {} \le Cs \int \limits _0^\infty e^{-c/t} \frac{dt}{t^{1+s/2}} \le C. \end{aligned}$$

So, as claimed, we are left to prove the estimate for the $M=({\mathbb {R}}^n,g)$, $p =0$, $\varphi = \textrm{id}$, and $g_{ij}$ satisfying the assumptions of Proposition 2.19.

Recalling (36), notice that

37 $$\begin{aligned} k(x,z)= & {} K(x, x+z) = c_s \int \limits _0^\infty H(x,x+z,t) \frac{dt}{t^{1+s/2}}\nonumber \\= & {} c_s \int \limits _0^\infty \,h\bigg (\frac{A(x)z}{\sqrt{t}},x, t\bigg )\frac{dt}{t^{n/2 +1+s/2}}. \end{aligned}$$

Also, recalling that $h_\circ (z):= (4\pi )^{-n/2} e^{-|z|^2/4}$, we have

$$\begin{aligned} \begin{aligned} {\widehat{k}}(x,z)&=k(x,z)- \frac{\alpha _{n,s}}{|A(x)z|^{n+s}} = c_s \int \limits _0^\infty \bigg (h\bigg (\frac{A(x)z}{\sqrt{t}},x, t\bigg )- h_\circ \bigg (\frac{A(x)z}{\sqrt{t}}\bigg ) \bigg )\frac{dt}{t^{n/2 +1+s/2}} \\ {}&= c_s \int \limits _0^\infty {\widehat{h}}\bigg (\frac{A(x)z}{\sqrt{t}},x,t\bigg ) \frac{dt}{t^{n/2 +1+s/2}}, \end{aligned} \end{aligned}$$

Therefore using the heat kernel estimates from Proposition 2.19 (and noticing $|A(x)z|\ge \tfrac{1}{\sqrt{2}} |z|$ for all *x*, *z* by assumption) we obtain

$$\begin{aligned} \begin{aligned} |{\widehat{k}}(x,z)|&\le c_s \int \limits _0 \bigg |{\widehat{h}}\bigg (\frac{A(x)z}{\sqrt{t}},x,t\bigg ) \bigg | \frac{dt}{t^{n/2 +1+s/2}} \le Cs \int \limits _0^\infty \sqrt{t} \exp (-c |z|/\sqrt{t}) \frac{dt}{t^{n/2 +1+s/2}} \\&= C|z|^{1-n-s}. \end{aligned} \end{aligned}$$

This proves (18). Similarly, the estimates (19) follow differentiating (37) and using the corresponding estimates for derivatives of the heat kernel from Proposition 2.19.

Finally, (20) and (21) follow analogously integrating the heat kernel estimates in Lemmas 2.16 and 2.17, respectively. $\square$

The next property concerns the behavior of the kernel when the two points *p* and *q* are separated from each other.

##### Proposition 2.21

Let (*M*, *g*) be a Riemannian *n*-manifold and $s\in (0,2)$. Assume that for some $p,q\in M$ both $\mathrm{FA_\ell }(M, g, 1, p, \varphi _p)$ and $\mathrm{FA_\ell }(M, g, 1, q, \varphi _q)$ hold, and suppose that $\varphi _p({\mathcal {B}}_1(0))\cap \varphi _q({\mathcal {B}}_1(0))= \varnothing$. Put $K_{pq}(x,y): = K_s(\varphi _p(x), \varphi _q(y))$. Then

$$\begin{aligned} \bigg |\frac{\partial ^{|\alpha |}}{\partial x^{\alpha }} \frac{\partial ^{|\beta |}}{\partial y^{\beta }} K_{pq}(x,y)\bigg | \le C(n,\ell )\quad \text{ for } \text{ all } |x|<\tfrac{1}{2} \text{ and } |y|< \tfrac{1}{2}, \end{aligned}$$

whenever $$|\alpha |+|\beta | \le \ell $$.

##### Proof

Let $H_*(x,y,t): =H_M(\varphi _p(x), \varphi _q(y),t)$. It follows from Lemma 2.16 that

$$\begin{aligned} \left| \frac{\partial ^{|\alpha |}}{\partial x^{\alpha }} H_*(x,y,t) \right| \le C \exp (-c/t) \end{aligned}$$

for all $|x|<\tfrac{3}{4}$ and $|y|< \tfrac{3}{4}$, where *C* and *c* depend only on *n*, and $|\alpha |$.

We now use that (by the symmetry of the heat kernel in *p* and *q*), for each $x\in {\mathcal {B}}_{1/2}$ fixed, the function $u(y,t): = \frac{\partial ^{|\alpha |}}{\partial x^{\alpha }} H_*(x,y,t)$ is solution of the heat equation $u_t = L u$, in the ball $|y|<1$, where *L* denotes the Laplace–Beltrami (with respect to *y*, in local coordinates). Since $|u|\le C\exp (-c/t)$ in ${\mathcal {B}}_{3/4}\times (0,\infty )$, reasoning exactly as in the proof of Lemma 2.16 (only that now the spatial variables are *y* instead of *x*) we obtain

$$\begin{aligned} \bigg |\frac{\partial ^{|\beta |}}{\partial y^{\beta }} u(y,t)\bigg |\le C \exp (-c/t), \end{aligned}$$

for some new positive constants *C* and *c* depending only on *n*, and $|\beta |$. This shows:

$$\begin{aligned} \bigg | \frac{\partial ^{|\alpha |}}{\partial x^{\alpha }}\frac{\partial ^{|\beta |}}{\partial y^{\beta }} H_*(x,y,t)\bigg |\le C\exp (-c/t) \end{aligned}$$

Then the proposition follows immediately after noticing that, by definition,

$$\begin{aligned} K_{pq}(x,y) = \frac{s/2}{\Gamma (1-s/2)} \int \limits _0^\infty H_*(x,y,t) \frac{dt}{t^{1+s/2}}, \end{aligned}$$

and hence

$$\begin{aligned} \bigg |\frac{\partial ^{|\alpha |}}{\partial x^{\alpha }} \frac{\partial ^{|\beta |}}{\partial y^{\beta }} K_{pq}(x,y)\bigg |= & {} \bigg | \frac{s/2}{\Gamma (1-s/2)} \int \limits _0^\infty \frac{\partial ^{|\alpha |}}{\partial x^{\alpha }}\frac{\partial ^{|\beta |}}{\partial y^{\beta }} H_*(x,y,t) \frac{dt}{t^{1+s/2}}\bigg |\\\le & {} Cs\int \limits _0^\infty \exp (-c/t)\frac{dt}{t^{1+s/2}} \le C , \end{aligned}$$

for some constant $C>0$ that depends only on *n* and $\ell$, and this concludes the proof. $\qquad \square$

### Extension definition

#### Definition 2.22

We define the weighted Sobolev space

$$\begin{aligned} \widetilde{H}^1({\mathbb {R}}^n \times (0,\infty )) = H^1({\mathbb {R}}^n \times (0,\infty ), z^{1-s}dxdz) \end{aligned}$$

as the completion of $C_c^\infty ({\mathbb {R}}^n \times [0,\infty ))$ with the norm

$$\begin{aligned} \Vert U\Vert ^2_{{\widetilde{H}}^1}:= \Vert U\Vert ^2_{L^2({\mathbb {R}}^n \times (0,\infty ), z^{1-s}dxdz)} + \Vert \widetilde{D} U\Vert ^2_{L^2({\mathbb {R}}^n \times (0,\infty ), z^{1-s}dxdz)} , \end{aligned}$$

where $\widetilde{D}U =\big (\frac{\partial U}{\partial x^1}, \dotsc , \frac{\partial U}{\partial x^n}, \frac{\partial U}{\partial z} \big )$ denotes the Euclidean gradient in ${\mathbb {R}}^{n+1}$. This is a Hilbert space with the natural inner product that induces the norm above. It is a known fact that any $U \in \widetilde{H}^1({\mathbb {R}}^n \times (0,\infty ))$ has a well defined trace in $L^2({\mathbb {R}}^n)$ that we denote by $U(x, \cdot )$.

The following essential result by Caffarelli and Silvestre shows that fractional powers of the Laplacian on ${\mathbb {R}}^n$ can be realized as a Dirichlet-to-Neumann map via an extension problem.

#### Theorem 2.23

([9]) Let $s \in (0,2)$ and $u \in H^{s/2}({\mathbb {R}}^n) \cap C^\infty ({\mathbb {R}}^n)$. Then, there is a unique solution $U = U(x,z): {\mathbb {R}}^n \times [0, +\infty ) \rightarrow {\mathbb {R}}$ among functions in $\widetilde{H}^1({\mathbb {R}}^n \times (0,\infty ))$ to the problem

38 $$\begin{aligned} {\left\{ \begin{array}{ll} \Delta _x U + \frac{\partial ^2 U}{\partial z^2} +\frac{1-s}{z} \frac{\partial U}{\partial z} =0\,, &{} \text{ on } \hspace{7pt}{\mathbb {R}}^n \times (0, \infty ) \\ U(x,0) = u(x) &{} \text{ for } \hspace{7pt}x \in {\mathbb {R}}^n\,, \end{array}\right. } \end{aligned}$$

and it satisfies

39 $$\begin{aligned} \lim _{z \rightarrow 0^+} z^{1-s} \frac{\partial U}{\partial z}(x,z) = - \beta _s^{-1} \, (-\Delta )^{s/2} u(x) , \end{aligned}$$

where $\Delta _x$ denotes the standard Laplacian on ${\mathbb {R}}^n$ and $\beta _s$ is a positive constant that depends only on *s*.

In [9], three different proofs of this fact are presented, but each of these proofs relies on some additive structure of the base space. To prove that the extension procedure produces the fractional power of the Laplacian also on a Riemannian manifold, which is the setting we are interested in, one has to rely on different ideas. It was proved by Stinga [21] that the unique solution to (38) verifying (39) admits the explicit representation

40 $$\begin{aligned} U(p,z)= \frac{z^{s}}{2^s\Gamma (s/2)} \int \limits _{0}^{\infty } P_t u (p) \, e^{-\frac{z^2}{4t}}\frac{dt}{t^{1+s/2}}, \end{aligned}$$

which expresses *U* in terms of the solution to the heat equation $P_t u$ (and thus makes sense also on a manifold). The proof of this fact does not strongly rely on the additive structure of ${\mathbb {R}}^n$ and will be proved now also on closed Riemannian manifolds.

First, let us define the weighted Sobolev spaces for the extension on compact manifolds.

#### Definition 2.24

We define the weighted Sobolev space

$$\begin{aligned} \widetilde{H}^1 ({\widetilde{M}}) = \widetilde{H}^1( M \times (0,\infty )) \end{aligned}$$

as the completion of $C_c^\infty ( M \times [0,\infty ))$ with the norm

$$\begin{aligned} \Vert U\Vert ^2_{{\widetilde{H}}^1}:= \Vert TU \Vert ^2_{L^2(M)} + \Vert {{\widetilde{\nabla }}} U\Vert ^2_{L^2({\widetilde{M}}, z^{1-s}dVdz)} , \end{aligned}$$

where $TU = U(\cdot , 0)$ is the trace of *U* and ${{\widetilde{\nabla }}} U =(\nabla U, U_z)$ denotes the gradient in $M\times (0,+\infty )$. This is a Hilbert space with the natural inner product that induces the norm above. Moreover, basically by definition, any $U \in \widetilde{H}^1 ({\widetilde{M}})$ leaves a trace in $L^2(M\times \{0\})$.

#### Theorem 2.25

Let $(M^n,g)$ be a closed Riemannian manifold, let $s\in (0,2)$ and $u:M\rightarrow {\mathbb {R}}$ be smooth. Consider the product manifold $\widetilde{M}=M\times (0,+\infty )$ endowed with the natural product metric.^5^ Then, there is a unique solution $U:M \times (0, \infty ) \rightarrow {\mathbb {R}}$ among functions in ${\widetilde{H}}^1({\widetilde{M}} )$ to

41 $$\begin{aligned} {\left\{ \begin{array}{ll} \widetilde{ \textrm{div}}(z^{1-s} {{\widetilde{\nabla }}} U) = 0 &{} \text{ in } \hspace{7pt}\widetilde{M} , \\ U(p,0) = u(p) &{} \text{ for } \hspace{7pt}p \in \partial \widetilde{M} = M, \end{array}\right. } \end{aligned}$$

given by (40), and it satisfies

42 $$\begin{aligned}{}[u]^2_{H^{{s/2}}(M)} = 2\beta _s \int \limits _{\widetilde{M}} |{{\widetilde{\nabla }}} U|^2 z^{1-s} \, dVdz , \end{aligned}$$

where $[u]^2_{H^{{s/2}}(M)}$ is defined through (2) and

43 $$\begin{aligned} \beta _s = \frac{2^{s-1} \Gamma (s/2)}{\Gamma (1-s/2)}. \end{aligned}$$

Moreover,

44 $$\begin{aligned} \lim _{z \rightarrow 0^+} z^{1-s} \frac{\partial U}{\partial z}(p,z) = -\beta _s^{-1} (-\Delta )^{s/2} u(p) , \end{aligned}$$

where the fractional Laplacian on the right-hand side is defined by either (7) or (8).

#### Proof

Note that functions in ${\widetilde{H}}^1({\widetilde{M}} )$ leave a well defined trace (that is, there exists a continuous trace operator with respect to the norm on ${\widetilde{H}}^1({\widetilde{M}} )$) on $M\times \{0\}$. Then, the fact that a solution among functions in ${\widetilde{H}}^1({\widetilde{M}} )$ exists follows by direct minimization of the associated energy $v \mapsto \int _{\widetilde{M}} |{{\widetilde{\nabla }}} v|^2z^{1-s} dVdz$ over ${\widetilde{H}}^1({\widetilde{M}})$. Since the energy is convex, the solution is also unique.

From here we divide the proof in two steps.

**Step 1.** We show that the (unique) solution $U \in {\widetilde{H}}^1({\widetilde{M}})$ to (41) is given by (40).

Making the identification $T(M \times (0,+\infty ) ) \simeq TM \times (0,+\infty )$ we have

$$\begin{aligned} \widetilde{ \textrm{div}}(z^{1-s} {{\widetilde{\nabla }}} U)&= \text {div}_g(z^{1-s} \nabla U) + \frac{d}{dz}(z^{1-s} U_z) \\ {}&= z^{1-s} \Delta U + (1-s)z^{-s} U_z + z^{1-s}U_{zz} \\ {}&= z^{1-s}\left( \Delta U + \frac{1-s}{z}U_z + U_{zz}\right) . \end{aligned}$$

Thus, in order to prove that *U* solves $\widetilde{ \textrm{div}}(z^{1-s} {{\widetilde{\nabla }}} U) = 0$ we show that *U* (weakly) solves

45 $$\begin{aligned} {\mathcal {L}}(U):= \Delta U + \frac{1-s}{z}U_z + U_{zz} =0 . \end{aligned}$$

Define

46 $$\begin{aligned} G(z,t):= \frac{1}{2^s \Gamma (s/2)} \frac{ z^{s}e^{-\frac{z^2}{4t}}}{t^{1+s/2}}, \end{aligned}$$

so that (40) rewrites simply as

47 $$\begin{aligned} U(\cdot ,z)=\int \limits _{0}^{\infty } (P_t u)G(z,t)\,dt. \end{aligned}$$

It can be easily checked that *G* satisfies

48 $$\begin{aligned} -G_t+\frac{1-s}{z}G_z+G_{zz} =0, \end{aligned}$$

and also

$$\begin{aligned} \lim _{t \rightarrow 0^+} \sup _{[z_1,z_2]} G(\cdot ,t) = 0 , \quad \lim _{t \rightarrow \infty } \sup _{[z_1,z_2]} G(\cdot ,t) = 0 , \end{aligned}$$

for every $[z_1,z_2]\subset (0,+\infty )$. Moreover, from the definition of *G* and the fact that *u* is smooth we se that the integral in the right-hand side of (47) is absolutely convergent in ${\widetilde{H}}^1({\widetilde{M}})$. Hence $U \in {\widetilde{H}}^1({\widetilde{M}})$.

Now we check that *U* weakly solves the desired problem. Let $\varphi \in C_c^\infty ({\widetilde{M}})$, $K:= \textrm{supp} (\varphi )$ and $z_1, z_2 \in (0,+\infty )$ such that $K\subset \subset M\times [z_1, z_2]$. Let also

$$\begin{aligned} {\mathcal {L}}^*(\varphi ):= \Delta \varphi +\partial _z \left( \frac{1-s}{z} \varphi \right) +\varphi _{zz} . \end{aligned}$$

This is the formal adjoint of the operator in (45). Clearly ${\mathcal {L}}^*(\varphi )$ still has compact support in $K\subset \subset {\widetilde{M}}$ and is smooth. Then

$$\begin{aligned} \int \limits _{{\widetilde{M}}} U {\mathcal {L}}^*(\varphi ) \, dV dz = \int \limits _{{\widetilde{M}}} \int \limits _{0}^\infty (P_t u) G(z,t) {\mathcal {L}}^*(\varphi ) \, dt dV dz , \end{aligned}$$

and we claim that this integral is absolutely convergent. Indeed

$$\begin{aligned} \int \limits _{{\widetilde{M}}} \int \limits _{0}^\infty&|(P_t u) G(z,t) {\mathcal {L}}^*(\varphi )| \, dt \, dV dz \le \Vert {\mathcal {L}}^*(\varphi )\Vert _{L^\infty } \int \limits _0^\infty \int \limits _K |P_t u ||G(z,t)| \, dV dz dt\\ {}&\le C \left( \int \limits _K |P_t u |^2|G(z,t)|^2 z^{1-s} \, dV dz \right) ^{\frac{1}{2}} \left( \int \limits _K \frac{1}{z^{1-s}} \, dV dz \right) ^{\frac{1}{2}} <+\infty , \end{aligned}$$

since the integral in (47) is absolutely convergent in ${\widetilde{H}}^1({\widetilde{M}})$. Hence we can exchange the order of integration and we get, integrating by parts in space many times

$$\begin{aligned}&\int \limits _{{\widetilde{M}}} U {\mathcal {L}}^*(\varphi ) \, dV dz = \int \limits _{0}^\infty \left( \int \limits _{K} (P_t u) G(z,t) {\mathcal {L}}^*(\varphi ) \, dV dz \right) dt \\&\quad = \int \limits _{0}^\infty \int \limits _{K} \left( G(z,t) \Delta (P_t u) + (P_t u) \frac{1-s}{z} G_z(z,t) + (P_t u) G_{zz}(z,t) \right) \varphi \, dVdz dt. \end{aligned}$$

Since $$P_t u $$ is smooth and solves the heat equation, the first term equals to

$$\begin{aligned} \int \limits _{0}^\infty \int \limits _{K} G(z,t) \Delta (P_t u) \, dV dzdt&= \int \limits _{0}^\infty \int \limits _{K} G(z,t) \partial _t(P_t u) \, dV dzdt\\&= \int \limits _{K} \int _{0}^\infty G(z,t) \partial _t(P_t u) \, dtdVdz \\ {}&= \int \limits _K (P_t u) G(z,t) \, dVdz \,\bigg |_{0^+}^\infty \\&- \int \limits _{K} \int \limits _{0}^\infty (P_t u ) G_t(z,t) \, dtdVdz. \end{aligned}$$

The boundary terms vanish since

$$\begin{aligned} \left| \int \limits _K (P_t u) G(z,t) \, dV dz \right|&\le \left( \int \limits _M |P_t u | \, dV \right) \left( \int \limits _{z_1}^{z_2} |G(z,t)| \, dz \right) \\ {}&\le |M|^{1/2} \Vert u \Vert _{L^2(M)}|z_2-z_1| \sup _{[z_1, z_2]} G(\cdot , t) \rightarrow 0, \end{aligned}$$

both as $t\rightarrow \infty$ and as $t\rightarrow 0^+$. Hence, putting all together and using (48)

$$\begin{aligned} \int \limits _{{\widetilde{M}}} {\mathcal {L}}(U) \varphi \, dVdz&= \int \limits _{{\widetilde{M}}} U {\mathcal {L}}^*(\varphi ) \, dV_p dz\\&= \int \limits _K \int \limits _0^\infty (P_t u) \left( -G_t +\frac{1-s}{z} G_z + G_zz \right) \varphi \, dVdzdt = 0 . \end{aligned}$$

Hence *U* given by (40) is a weak solution of (45), and by standard elliptic regularity it is also a classical solution.

Moreover, the fact that $U(\cdot , 0^+)=u$ follows by the explicit formula (40). Indeed, by a simple change of variable in the integral we have

$$\begin{aligned} U(p,z) = \frac{1}{\Gamma (s/2)} \int \limits _0^\infty (P_{z^2/4r} u)(p) \frac{e^{-r}}{r^{1-s}} dr , \end{aligned}$$

and taking $z\rightarrow 0^+$ in this formula gives $U(\cdot , 0^+)=u$. This concludes Step 1.

**Step 2.** Proof of (44).

Note that by the representation formula we just proved for *U* we have

$$\begin{aligned} \tau ^s_z U (p):= \frac{U(p,z)-u(p)}{z^{s}} = \frac{1}{2^s\Gamma (s/2)} \int \limits _{0}^{\infty } (P_tu(p)-u(p))e^{-\frac{z^2}{4t}} \frac{dt}{t^{1+s/2}}. \end{aligned}$$

Moreover, by L’Hopital’s rule

$$\begin{aligned} \lim _{z\rightarrow 0^+} \tau ^s_z U = \lim _{z \rightarrow 0^+} s^{-1} z^{1-s} \frac{\partial U}{\partial z} . \end{aligned}$$

Writing $$P_t u $$ as the convolution against the heat kernel $$H_M$$ of *M* we get

$$\begin{aligned} \tau ^s_z U (p) = \frac{1}{2^s\Gamma (s/2)} \int \limits _{0}^{\infty } \int \limits _M H_M(p,q,t)(u(q)-u(p)) \frac{e^{-\frac{z^2}{4t}}}{t^{1+s/2}} dV_q dt. \end{aligned}$$

Since *u* is smooth, and since $K_s^\varepsilon (p,q)$ is non-singular for $\varepsilon >0$ on the diagonal $\{p=q\}$ (recall (10)) there holds

$$\begin{aligned}&\int \limits _M \frac{s/2}{\Gamma (1-s/2)}\int \limits _{0}^{\infty } H_M(p,q,t)|u(q)-u(p)| \frac{e^{-\frac{z^2}{4t}}}{t^{1+s/2}} dt dV_q\\&\quad = \int \limits _M |u(q)-u(p)| K_s^z(p,q) \, dV_q <+\infty . \end{aligned}$$

Thus the integral in $\tau ^s_z U (p)$ is absolutely convergent, and we can exchange the order of integration to get

$$\begin{aligned} \tau ^s_z U (p) = \frac{\Gamma (1-s/2)}{s 2^{s-1}\Gamma (s/2)} \int \limits _M (u(q)-u(p)) K_s^z(p,q) \, dV_q . \end{aligned}$$

From here, by the very definition of the principal value

$$\begin{aligned} \lim _{z \rightarrow 0^+} z^{1-s} \frac{\partial U}{\partial z} = \lim _{z\rightarrow 0^+} s \cdot \tau ^s_z U&= \lim _{z\rightarrow 0^+} \beta _s^{-1} \int \limits _M (u(q)-u) K_s^z(p,q) \, dV_q \\ {}&= - \beta _s^{-1} \left( p.v. \int \limits _M (u-u(q)) K_s(p,q) \, dV_q \right) \\ {}&= -\beta _s^{-1} (-\Delta )^{s/2} u. \end{aligned}$$

This finishes Step 2.

Before proving (42) we prove also that the convergence in (44) holds in $L^r(M)$ for every $r\in [1, +\infty )$. Since we have pointwise convergence, we show that the sequence is dominated. In particular, we prove that for $z\le 1$ there holds

49 $$\begin{aligned} z^{1-s} | U_z(\cdot , z)| \le C , \end{aligned}$$

where *C* depends on $\Vert \Delta u\Vert _{L^\infty }$, $\Vert u\Vert _{L^\infty }$ and *s*. The proof is a standard barrier argument very similar to the proof of Lemma 3.5, we just sketch the argument. Consider $b(p,z):=u(p)-C(z^2-z^s)$, for $C>0$ that will be chosen soon. Since

$$\begin{aligned} \widetilde{ \textrm{div}}(z^{1-s} {{\widetilde{\nabla }}} b) = z^{1-s}(\Delta u - 2sC) , \end{aligned}$$

we see that *b* is a subsolution of (41) if we take $C=\tfrac{1}{2s}\Vert \Delta u\Vert _{L^\infty }$. Moreover $U \le b$ on $M\times [0, 1]$. Hence *b* is barrier for *U*, and by the maximum principle for $z\le 1$ we have

$$\begin{aligned} U(\cdot ,z) \le b(\cdot , z) = u-\tfrac{1}{2s}\Vert \Delta u\Vert _{L^\infty }(z^2-z^s) \le u+ \tfrac{z^s}{2s}\Vert \Delta u\Vert _{L^\infty } , \end{aligned}$$

and this implies

$$\begin{aligned} \lim _{z \rightarrow 0^+} z^{1-s} U_z = \lim _{z\rightarrow 0^+} s \frac{U(\cdot , z)-u}{z^s} \le \tfrac{1}{2}\Vert \Delta u\Vert _{L^\infty } . \end{aligned}$$

Completely analougusly using $-b$ as a barrier for $-U$ one gets also the reverse inequality. Moreover, note that the function $V:=z^{1-s}U_z$ solves $-(\Delta V + V_{zz})+\tfrac{1-s}{z}V_z =0$, thus by the maximum principle

$$\begin{aligned} \sup _{M\times (0,1] } |V| \le \max \left\{ \sup _{M} V(\cdot , 0^+), \sup _{M} V(\cdot ,1) \right\} \le \max \left\{ \tfrac{1}{2}\Vert \Delta u\Vert _{L^\infty }, \sup _{M} V(\cdot ,1) \right\} . \end{aligned}$$

But since $V(\cdot ,1)= U_z(\cdot ,1)$ we have by standard interior gradient estimates

$$\begin{aligned} |U_z(p,1)| \le C \sup _{{\widetilde{B}}_{1/10}(p,1)} |U| \le C\Vert u\Vert _{L^\infty } , \end{aligned}$$

for some absolute constant $C>0$ independent of *u*. Putting everything together

$$\begin{aligned} \sup _{M\times (0,1] } |V| = \sup _{M\times (0,1] } z^{1-s}|U_z| \le C(\Vert \Delta u\Vert _{L^\infty }, \Vert u\Vert _{L^\infty }) , \end{aligned}$$

and this concludes the proof of (49).

We’re left with proving (42). Integrating by parts, for every $\delta >0$ we find that

$$\begin{aligned} \int \limits _{M\times [\delta , \infty )} |{{\widetilde{\nabla }}} U|^2 z^{1-s} \, dVdz = \beta _s^{-1}\int \limits _{M} U(\cdot , \delta ) \delta ^{1-s}U_z(\cdot , \delta ) \, dV . \end{aligned}$$

Letting now $\delta \rightarrow 0^+$, by (44), (49) and dominated convergence on the right-hand side

$$\begin{aligned} \int \limits _{{\widetilde{M}}} |{{\widetilde{\nabla }}} U|^2 z^{1-s} \, dVdz = \beta _s^{-1} \int _M u(-\Delta )^{s/2} u \, dV = \frac{\beta _s^{-1}}{2} [u]^2_{H^{s/2}(M)} . \end{aligned}$$

This concludes the proof. $\quad \square$

We have just used the following fact, which is proved with a one-line computation using (8).

#### Proposition 2.26

For a smooth function, one has that

$$\begin{aligned}{}[u]_{H^{s/2}(M)}^2 = 2\int \limits _{M} u(-\Delta )^{s/2} u \, dV. \end{aligned}$$

#### Remark 2.27

Let us briefly comment on the role played by the energy space for the uniqueness of the extension in Theorem 2.25. One can note that, for every $C>0$, the function $V=Cz^s$ on ${\widetilde{M}}$ is a solution of $\widetilde{ \textrm{div}}(z^{1-s} {{\widetilde{\nabla }}} V) = 0$ with zero trace. Hence, uniqueness outside the energy space ${\widetilde{H}}^1({\widetilde{M}})$ does not hold in general, and every uniqueness result that does not rely on being in ${\widetilde{H}}^1({\widetilde{M}})$ must, in particular, rule out this phenomenon.

A simple uniqueness result that does not rely on the energy space is the following. Let *U* solve $\widetilde{ \textrm{div}}(z^{1-s} {{\widetilde{\nabla }}} U) = 0$, with $U(\cdot , 0)=0$ be such that

$$\begin{aligned} \limsup _{z\rightarrow \infty } \sup _{p\in M} |U(p,z)| z^{-s}=0 . \end{aligned}$$

That is, *U* grows at infinity slower than any multiple of $z^s$. Then $U\equiv 0$.

This can be proved using $Cz^s$ as a barrier. Indeed, this is a solution of $\widetilde{ \textrm{div}}(z^{1-s} {{\widetilde{\nabla }}} U) = 0$, with $U(\cdot , 0)=0$. By the growth hypothesis on *U* we have that there exists *C* large (depending on *U*) such that $Cz^s \ge 10 U$ on ${\widetilde{M}}$. In particular, with this *C*, we have $U < Cz^s$.

Now start decreasing *C*. The graph of $Cz^s$ can never touch *U* from above since this would contradict the maximum principle in the interior. Hence $U < Cz^s$ for every $C>0$ and sending $C\rightarrow 0^+$ gives $U\le 0$.

By the same argument from below one also gets $U\ge 0$. Hence $U\equiv 0$ and this concludes the proof.

## The fractional Sobolev energy

### Several definitions and their equivalence

We recall the definition for the fractional Sobolev seminorm that we have used in the previous section, and we define the associated functional space.

#### Definition 3.1

We define the fractional Sobolev seminorm $[u]_{H^{s/2}(M)}$ for $s \in (0,2)$ as

50 $$\begin{aligned}{}[u]^2_{H^{{s/2}}(M)} =\int \!\int _{M\times M} (u(p)-u(q))^2 K_s(p,q) \, dV_p dV_q \,. \end{aligned}$$

The associated functional space $H^{s/2}(M)$ is

51 $$\begin{aligned} H^{s/2}(M)=\{u\in L^2(M) \text {: } [u]^2_{H^{s/2}(M)}<\infty \} , \end{aligned}$$

and it is called the *fractional Sobolev space of order* *s*/2. This is a Hilbert space with norm given by

$$\begin{aligned} \Vert u\Vert _{H^{{s/2}}(M)}^2=\Vert u\Vert _{L^2(M)}^2+[u]^2_{H^{{s/2}}(M)}\,. \end{aligned}$$

The fractional Sobolev seminorm can also be expressed using spectral or extension approaches:

#### Proposition 3.2

Let $u\in H^{s/2}(M)$. Then, the fractional Sobolev seminorm (50) is equal to

52 $$\begin{aligned}{}[u]^2_{H^{{s/2}}(M)} = 2\sum _{k=1}^\infty \lambda _k^{s/2} \langle u, \phi _k\rangle _{L^2(M)} ^2 \end{aligned}$$

and

53 $$\begin{aligned}{}[u]^2_{H^{{s/2}}(M)} = \inf _{v \in {\widetilde{H}}^1({\widetilde{M}})} \left\{ 2\beta _s \int \limits _{\widetilde{M}} |{{\widetilde{\nabla }}} v|^2 z^{1-s} \, dVdz \,: \, v(\cdot ,0)=u(\cdot ) \text{ in } L^2(M) \right\} .\nonumber \\ \end{aligned}$$

Moreover, the conclusions of Theorem 2.25 also hold for *u* (with the exception of (44)), and the infimum in (53) is attained by the unique $U \in {\widetilde{H}}^1({\widetilde{M}})$ given by Theorem 2.25. In particular, we also have that

54 $$\begin{aligned}{}[u]^2_{H^{s/2}(M)}= 2\beta _s \int \limits _{\widetilde{M}} |{\widetilde{\nabla }} U|^2 z^{1-s} \, dVdz , \end{aligned}$$

where $\beta _s$ is the constant defined in (43).

#### Proof

**Step 1.** We show that (50) and (52) coincide for a function in $L^2(M)$.

Recall the regularised kernel $K_s^\varepsilon$ defined in (10), which is bounded, symmetrical and increases monotonically to $K_s$ as $\varepsilon \rightarrow 0$. By monotone convergence and these properties, for any function $u\in L^2(M)$ we can write

55 $$\begin{aligned}{}[u]^2_{H^{s/2}(M)}&=\int \!\int \limits _{M\times M} (u(p)-u(q))^2 K_s(p,q) \, dV_p dV_q \nonumber \\&= \lim _{\varepsilon \rightarrow 0} \int \!\int \limits _{M\times M} (u(p)-u(q))^2 K_s^\varepsilon (p,q)\, dV_p dV_q \nonumber \\&= \lim _{\varepsilon \rightarrow 0} \,2\int \!\int \limits _{M\times M} (u(p)-u(q))u(p) K_s^\varepsilon (p,q)\, dV_p dV_q \nonumber \\&= \lim _{\varepsilon \rightarrow 0} \,2\int \limits _{M} ((-\Delta )^{s/2}_\varepsilon u)(p) u(p) K_s^\varepsilon (p,q)\, dV_p\, , \end{aligned}$$

where we have set

$$\begin{aligned} ((-\Delta )^{s/2}_\varepsilon u)(p)&:=\int \limits _M (u(p)-u(q)) K_s^\varepsilon (p,q)\,dV_q\\&=\frac{s/2}{\Gamma (1-s/2)} \int \limits _M (u(p)-u(q)) \int \limits _{0}^{\infty } H_M(p,q,t) e^{-\varepsilon ^2/4t} \frac{dt}{t^{1+s/2}}\,dV_q\\&=\frac{s/2}{\Gamma (1-s/2)} \int \limits _{0}^{\infty }(u(p)-P_t u(p)) e^{-\varepsilon ^2/4t} \frac{dt}{t^{1+s/2}}\, . \end{aligned}$$

Now, if $(\phi _k)_{k \ge 0}$ is an orthonormal basis of $L^2(M)$ made of eigenfunctions for $(-\Delta )$, with eigenvalues

$$\begin{aligned} 0 = \lambda _0 < \lambda _1 \le \lambda _2 \le \dotsc \le \lambda _k \xrightarrow {\,k \rightarrow \infty \,} +\infty \,, \end{aligned}$$

then they are also eigenfunctions for $(-\Delta )^{s/2}_\varepsilon$ with eigenvalues

$$\begin{aligned} \lambda _{k,\varepsilon }^{s/2}:= \frac{s/2}{\Gamma (1-s/2)} \int \limits _{0}^{\infty }(1-e^{-\lambda _k t}) e^{-\varepsilon ^2/4t} \frac{dt}{t^{1+s/2}}\,, \end{aligned}$$

which one sees immediately by applying the formula above for $(-\Delta )^{s/2}_\varepsilon$ to $\phi _k$ and using that $P_t \phi _k =e^{-\lambda _k t} \phi _k$. These eigenvalues are uniformly bounded in *k* (for a fixed $\varepsilon >0$) and increase monotonically to the $\lambda _{k}^{s/2}$ as $\varepsilon \rightarrow 0^+$.

Expanding $u=\sum _{k=0}^\infty a_k \phi _k$, with $a_k:=\langle u, \phi _k\rangle _{L^2(M)}$, we deduce that

$$\begin{aligned} (-\Delta )^{s/2}_\varepsilon u= \sum _{k=0}^\infty \lambda _{k,\varepsilon }^{s/2} \langle u, \phi _k\rangle _{L^2(M)} \phi _k\,. \end{aligned}$$

We remark that the expression makes sense since the $\lambda _{k,\varepsilon }^{s/2}$ are bounded uniformly in *k* (for a fixed $\varepsilon$), and thus the sum is absolutely convergent in $L^2(M)$. Using this fact, substituting into (55) gives that

$$\begin{aligned}{}[u]^2_{H^{s/2}(M)}&=\lim _{\varepsilon \rightarrow 0} \,2\int \limits _{M} ((-\Delta )^{s/2}_\varepsilon u)(p) u(p) K_s^\varepsilon (p,q)\, dV_p =\lim _{\varepsilon \rightarrow 0} \,2\sum _{k=0}^\infty \lambda _{k,\varepsilon }^{s/2} a_k^2\, . \end{aligned}$$

Using again the monotone convergence theorem (for sums now), we deduce that

$$\begin{aligned}{}[u]^2_{H^{s/2}(M)}&=2\sum _{k=0}^\infty \lambda _{k}^{s/2} a_k^2 \end{aligned}$$

as desired.

**Step 2.** We show that *U* given by the representation formula (40), which was only used for smooth functions *u*, is valid for $u\in H^{s/2}(M)$ in general and moreover (54) still holds with this *U*.

Fix $u \in H^{s/2}(M)$, and let *U* be defined through the representation formula (40). We will first show that *U* has finite ${\widetilde{H}}^1({\widetilde{M}})$ energy, using the spectral expression (52) for the energy that we have just proved. Recall that if $\phi _k$ is an eigenfunction of $(-\Delta )$, then $P_t \phi _k =e^{-\lambda _k t} \phi _k$. Therefore, writing $u=\sum _{k=0}^\infty a_k \phi _k$, where $a_k:= \langle u, \phi _k\rangle _{L^2(M)}$, we have that

$$\begin{aligned} U(p,z)&= \frac{z^{s}}{2^s\Gamma (s/2)} \int \limits _{0}^{\infty } P_t u (p) \, e^{-\frac{z^2}{4t}}\frac{dt}{t^{1+s/2}}\\&=\frac{z^{s}}{2^s\Gamma (s/2)} \sum _{k=0}^\infty a_k \phi _k (p) \int \limits _{0}^{\infty } e^{-\lambda _k t - \frac{z^2}{4t}} \, \frac{dt}{t^{1+s/2}}\, . \end{aligned}$$

Then, we can compute (recall that $\nabla =\nabla _p$ denotes the gradient on *M*)

$$\begin{aligned} \nabla U(p,z)=\frac{z^{s}}{2^s\Gamma (s/2)} \sum _{k=1}^\infty a_k \nabla \phi _k (p) \int \limits _{0}^{\infty } e^{-\lambda _k t -\frac{z^2}{4t}} \, \frac{dt}{t^{1+s/2}}\, , \end{aligned}$$

and

$$\begin{aligned} \partial _z U(p,z)&= \frac{1}{2^s\Gamma (s/2)} \sum _{k=1}^\infty a_k\phi _k (p)\int \limits _{0}^{\infty } e^{-\lambda _k t -\frac{z^2}{4t}} \Big ( sz^{s-1} - \frac{z^{1+s}}{2t} \Big ) \frac{dt}{t^{1+s/2}} \\ {}&= \frac{z^{s-1}}{2^s\Gamma (s/2)} \sum _{k=1}^\infty a_k\phi _k (p)\int \limits _{0}^{\infty } e^{-\lambda _k t -\frac{z^2}{4t}} \Big ( s - \frac{z^2}{2t} \Big ) \frac{dt}{t^{1+s/2}}. \end{aligned}$$

Recall that the $\phi _i$ and $\phi _j$ are orthogonal in $L^2(M)$ and $H^1(M)$ seminorms for $i\ne j$, and that moreover $\int _M \phi _k^2=1$ and $\int _M |\nabla \phi _k |^2=\lambda _k$ for every *k*. Then, given $z\in {\mathbb {R}}_+$ we find that

$$\begin{aligned} \int \limits _{M\times \{z\}}|\nabla U(p,z)|^2dV_p&=\frac{z^{2s}}{2^{2s}\Gamma ^2(s/2)}\sum _{k=1}^\infty a_k^2 \left( \int \limits _{0}^{\infty } e^{-\lambda _k t -\frac{z^2}{4t}} \frac{dt}{t^{1+s/2}}\right) ^2 \int \limits _M |\nabla \phi _k|^2 \, dV \\&=\frac{z^{2s}}{2^{2s}\Gamma ^2(s/2)}\sum _{k=1}^\infty \lambda _ka_k^2 \left( \int \limits _{0}^{\infty } e^{-\lambda _k t -\frac{z^2}{4t}} \frac{dt}{t^{1+s/2}}\right) ^2 \\ {}&= \frac{z^{2s}}{2^{2s}\Gamma ^2(s/2)}\sum _{k=1}^\infty \lambda _k^{1+s}a_k^2 \left( \int \limits _{0}^{\infty } e^{-r-\frac{z^2\lambda _k}{4r}}\frac{dr}{r^{1+s/2}}\right) ^2 , \end{aligned}$$

where in the last line we have performed the change of variables $r=\lambda _k t$.

We can argue analogously for $\partial _z U$, which leads to

$$\begin{aligned} \int \limits _{M\times \{z\}} \big (\partial _z U(p,z)\big )^2dV_p&= \frac{z^{2s-2}}{2^{2s}\Gamma (s/2)^2} \sum _{k=1}^\infty a_k^2 \left( \int \limits _{0}^{\infty } e^{-\lambda _k t -\frac{z^2}{4t}} \Big ( s - \frac{z^2}{2t} \Big )\frac{dt}{t^{1+s/2}} \right) ^2 \\ {}&= \frac{z^{2s-2}}{2^{2s}\Gamma (s/2)^2} \sum _{k=1}^\infty a_k^2 \lambda _k^s \left( \int \limits _{0}^{\infty } e^{-r -\frac{z^2\lambda _k}{4r}} \Big ( s - \frac{z^2\lambda _k }{2r} \Big )\frac{dr}{r^{1+s/2}} \right) ^2. \end{aligned}$$

Now, multiplying by $z^{1-s}$ and integrating in *z* over $(0,\infty )$, and then performing the change of variables $z=\lambda _k^{-1/2} w$ (so that $z^2\lambda _k=w^2$), gives that

$$\begin{aligned}&\int \limits _{M\times {\mathbb {R}}_+}|\nabla U(p,z)|^2z^{1-s} dV_p\,dz\\&\quad =\frac{1}{2^{2s}\Gamma ^2(s/2)}\sum _{k=1}^\infty \lambda _k^{1+s}a_k^2 \int \limits _0^\infty z^{1+s}\left( \int \limits _{0}^{\infty } e^{-r -\frac{z^2\lambda _k}{4r}}\frac{dr}{r^{1+s/2}}\right) ^2 dz\\&\quad =\frac{1}{2^{2s}\Gamma ^2(s/2)}\sum _{k=1}^\infty \lambda _k^{s/2} a_k^2 \int \limits _0^\infty w^{1+s}\left( \int \limits _{0}^{\infty } e^{-r -\frac{w^2}{4r}}\frac{dr}{r^{1+s/2}}\right) ^2 dw \\&\quad =2 c_1(s)\sum _{k=1}^\infty \lambda _k^{s/2}a_k^2 =c_1(s)[u]_{H^{s/2}(M)}^2 , \end{aligned}$$

and similarly

$$\begin{aligned}&\int \limits _{M\times {\mathbb {R}}_+}|\partial _z U(p,z)|^2 z^{1-s} dV_p\,dz\\&\quad = \frac{1}{2^{2s}\Gamma ^2(s/2)} \sum _{k=1}^\infty \lambda _k^{s} a_k^2 \int \limits _0^\infty z^{s-1} \left( \int \limits _{0}^{\infty } e^{-r -\frac{z^2\lambda _k}{4r}} \Big ( s - \frac{z^2\lambda _k }{2r} \Big )\frac{dr}{r^{1+s/2}} \right) ^2 dz\\&=\frac{1}{2^{2s}\Gamma ^2(s/2)}\sum _{k=1}^\infty \lambda _k^{s/2} a_k^2 \int \limits _0^\infty w^{s-1} \left( \int \limits _{0}^{\infty } e^{-r -\frac{w^2}{4r}} \Big ( s - \frac{w^2}{2r} \Big )\frac{dr}{r^{1+s/2}} \right) ^2 dw \\ {}&=2 c_2(s)\sum _{k=1}^\infty \lambda _k^{s/2}a_k^2 =c_2(s)[u]_{H^{s/2}(M)}^2. \end{aligned}$$

Here, we have defined $c_1(s)$ and $c_2(s)$ implicitly as the corresponding constants (which depend only on *s*) resulting from the expression, and we have applied (52) in the last line in both computations.

Putting everything together, we get that

$$\begin{aligned} \int \limits _{M \times {\mathbb {R}}_+}|{{\widetilde{\nabla }}} U(p,z)|^2 z^{1-s} \, dV_p dz= \big (c_1(s)+c_2(s) \big ) [u]_{H^{s/2}(M)}^2. \end{aligned}$$

We could write the constant $\big (c_1(s)+c_2(s) \big )$ explicitly in terms of the resulting complicated integral expressions. On the other hand, thanks to (42) and (43) from Theorem 2.25 (which was proved only for smooth functions), we know that $c_1(s)+c_2(s) = (2\beta _s)^{-1}$. This proves (54) with *U* given by the representation formula (40).

In particular, we now know that *U* has finite energy for the extension problem. Moreover, arguing as in Step 1 of the proof of Theorem 2.25, it is simple to see that *U* has *u* as its trace in $L^2(M)$, and that it is a weak (meaning in duality with $C_c^\infty ({\widetilde{M}})$) solution to $\widetilde{ \textrm{div}}(z^{1-s} {{\widetilde{\nabla }}} U) = 0$. Let now $U_\textrm{min} \in {\widetilde{H}}^1({\widetilde{M}})$ be defined as the unique minimizer of (53). The fact that $U_\textrm{min}$ exists follows by a standard lower-semicontinuity argument, just as at the beginning of the proof of Theorem 2.25, together with the fact that the space of competitors is not empty (which holds since for example *U* defined above, which has finite ${\widetilde{H}}^1({\widetilde{M}})$ energy, is one such competitor). Clearly, $U_\textrm{min}$ is also a weak solution of $\widetilde{ \textrm{div}}(z^{1-s} {{\widetilde{\nabla }}} U_\textrm{min}) = 0$ with trace *u*.

**Step 3.** $$U=U_\textrm{min}$$.

This follows directly from the uniqueness of weak solutions shown in Lemma 3.3, which we state as a separate result after the present proof.

With this, we conclude the proof of Proposition 3.2. $\square$

#### Lemma 3.3

(Uniqueness of weak solutions) Let $u\in L^2(M)$, and denote by $T:{\widetilde{H}}^1({\widetilde{M}}) \rightarrow L^2(M)$ the trace operator. Then, there exists at most one solution $U \in {\widetilde{H}}^1({\widetilde{M}})$ to the problem

$$\begin{aligned} {\left\{ \begin{array}{ll} \widetilde{ \textrm{div}}(z^{1-s} {{\widetilde{\nabla }}} U ) = 0 &{} \text{ in } \text{ duality } \text{ with } \,\, C_c^\infty (M\times (0,\infty )) , \\ TU=u . \end{array}\right. } \end{aligned}$$

#### Proof

Suppose $U_1$ and $U_2$ are two such solutions and denote $V:=U_1-U_2$. By hypothesis $TV=0$.

We claim that there exists a sequence $(V_k)_k \in C^\infty _c(M\times (0,\infty ))$ such that

56 $$\begin{aligned} \int \limits _{{\widetilde{M}}} |{{\widetilde{\nabla }}} V_k - {{\widetilde{\nabla }}} V |^2 z^{1-s} \, dVdz \rightarrow 0 , \hspace{7pt}\text {as} \,\, k\rightarrow \infty . \end{aligned}$$

The point here being that $V_k$ is zero both in a neighborhood of $M\times \{0\}$ and in a neighborhood of infinity.

The proof is inspired by (a weighted version of) [16, Sect. 5.5, Theorem 2]. By the definition of the space ${\widetilde{H}}^1({\widetilde{M}})$ there exists a sequence $(U_k)_k \subset C^\infty (M \times [0,\infty ))$ with (as $k\rightarrow \infty$)

$$\begin{aligned} \int \limits _{{\widetilde{M}}} |{{\widetilde{\nabla }}} U_k - {{\widetilde{\nabla }}} V |^2 z^{1-s} \, dVdz \rightarrow 0 , \hspace{7pt}\text {and} \,\,\, TU_k = U_k(\cdot , 0) \rightarrow 0 \text{ in } L^2(M). \end{aligned}$$

Note also that *V* is smooth in $M\times (0, \infty )$. Now, for every $(p,z) \in {\widetilde{M}}$, by the fundamental theorem of calculus and Holder’s inequality

$$\begin{aligned} |U_k(p,z)|^2&\le 2|U_k(p,0)|^2 + 2\left( \int \limits _0^z |{{\widetilde{\nabla }}} U_k(p,y)| \, dy \right) ^2 \\ {}&\le C|U_k(p,0)|^2 + Cz^s \int \limits _0^z |{{\widetilde{\nabla }}} U_k(p,y)|^2 y^{1-s} \, dy \,, \end{aligned}$$

and integrating for $p\in M$ gives

$$\begin{aligned} \int \limits _M |U_k(p,z)|^2 \, dV_p \le C\int _M|U_k(\cdot ,0)|^2 + Cz^s \int \limits _M\int \limits _0^z |{{\widetilde{\nabla }}} U_k(p,y)|^2 y^{1-s} \, dy \, dV_p . \end{aligned}$$

Letting $k\rightarrow \infty$ we get

57 $$\begin{aligned} \int \limits _M |V(\cdot ,z)|^2 \, dV \le Cz^s \int \limits _M\int \limits _0^z |{{\widetilde{\nabla }}} V|^2 y^{1-s} \, dy \, dV_p . \end{aligned}$$

Now, for every $k\ge 10$, let $\eta _k \in C^\infty ([0,+\infty ))$ be a smooth cutoff function with $\eta =0$ on [0, 1/*k*], $\eta =1$ on $[2/k, \infty )$ and $|\eta '|\le C k$. We claim that the sequence $V \eta _k = V(p,z)\eta _k(z) \in C^{\infty }_c(M\times (0,\infty ))$ has the desired property. We have

$$\begin{aligned} \int \limits _{{\widetilde{M}}} |{{\widetilde{\nabla }}} (V \eta _k) - {{\widetilde{\nabla }}} V |^2 z^{1-s} \, dVdz&\le C \int \limits _{{\widetilde{M}}}| {{\widetilde{\nabla }}} V|^2 (1-\eta _k)^2 z^{1-s}\\&\quad + C \int \limits _{{\widetilde{M}}} |V|^2|\eta _k'|^2 z^{1-s} =: I_{1,k} + I_{2,k}. \end{aligned}$$

We estimate the two integrals separately.

For the first integral we have

$$\begin{aligned} I_{1,k} \le C \int \limits _{0}^{2/k} \int \limits _{M} | {{\widetilde{\nabla }}} V|^2 z^{1-s} \, dVdz \rightarrow 0 , \end{aligned}$$

as $k \rightarrow \infty$, since *V* has finite energy.

Moreover, by (57), we have regarding the second integral

$$\begin{aligned} I_{2,k}&\le C k^2 \int \limits _{0}^{2/k} \int \limits _M z^{1-s} |V|^2 \, dVdz \\ {}&\le C k^2 \int \limits _{0}^{2/k} z^{1-s} \left( z^s \int \limits _M \int \limits _0^z |{{\widetilde{\nabla }}} V|^2y^{1-s} \, dydV \right) dz \\ {}&\le C k^2 \left( \int \limits _{0}^{2/k} z \, dz \right) \left( \int \limits _0^{2/k} \int \limits _M |{{\widetilde{\nabla }}} V|^2 y^{1-s} \, dVdy \right) \\ {}&= C \int \limits _0^{2/k} \int \limits _M |{{\widetilde{\nabla }}} V|^2 y^{1-s} \, dVdy \rightarrow 0 \end{aligned}$$

as $k\rightarrow \infty$, again as *V* has finite energy.

Hence $V_k:= V\eta _k$ has the desired property (56), and it can be used as a test function in the weak formulation in duality with $C_c^\infty ({\widetilde{M}})$. Multiplying $\widetilde{ \textrm{div}}(z^{1-s} {{\widetilde{\nabla }}} V ) = 0$ by $V_k$, integrating on ${\widetilde{M}}$ and integrating by parts gives

$$\begin{aligned} \int \limits _{{\widetilde{M}}} ( {{\widetilde{\nabla }}} V \cdot {{\widetilde{\nabla }}} V_k) z^{1-s} \, dVdz =0 . \end{aligned}$$

Letting $k\rightarrow \infty$ and using (56) gives

$$\begin{aligned} \int \limits _{{\widetilde{M}}} | {{\widetilde{\nabla }}} V |^2 z^{1-s} \, dVdz =0 , \end{aligned}$$

hence *V* is constant, and then (since $TV=0$) it must be $V\equiv 0$. Thus, $U_1=U_2$ coincide, and the proof is complete. $\square$

### A note on noncompact manifolds

In this subsection, we briefly describe if and how the given previous definitions of the spaces $H^{s/2}(M)$ generalize to the case of complete, noncompact Riemannian manifolds (without boundary).

First, let us stress that all the properties and estimates for the heat kernel $H_M$, and thus also for the singular kernel $K_s$, in Sect. 2.2 hold for every complete Riemannian manifold (not necessarily compact).

Recall definitions (i)–(iii) from the introduction (see (1)–(4)). First, let us rewrite definitions (i) and (ii) of the $H^{s/2}$ seminorm, still on a closed manifold *M*, exploiting the corresponding fractional Laplacians. Indeed, note that definition (2) can be rewritten (say, for smooth functions) as

$$\begin{aligned}{}[u]_{H^{s/2}(M)}^2 = 2\int \limits _M u (-\Delta )^{s/2}_\textrm{Si} u \, dV , \end{aligned}$$

where $(-\Delta )^s_\textrm{Si}$ is the singular integral fractional Laplacian given by (8). Similarly, the spectral definition (3) of the seminorm can be written as

$$\begin{aligned}{}[u]_{H^{s/2}(M)}^2 = 2\int \limits _M u (-\Delta )^{s/2}_\textrm{Spec} u \, dV , \end{aligned}$$

where $(-\Delta )^{s/2}_\textrm{Spec}$ is the spectral fractional Laplacian given by

58 $$\begin{aligned} (-\Delta )^{s/2}_\textrm{Spec} u = \sum _{k \ge 0} \lambda _k^{s/2} \langle u, \varphi _k \rangle _{L^2(M)} \varphi _k . \end{aligned}$$

Here, the convergence on the right-hand side is to be understood in $L^2(M)$.

Both of these definitions can be generalized to the case of a noncompact manifold, perhaps without equality between them anymore:

The singular integral definition (8) applies verbatim to the case of noncompact manifolds. This requires dealing with the heat kernel on noncompact manifolds. We refer to the survey [18] for the construction and properties of the heat kernel on complete, noncompact Riemannian manifolds. In the case of the Euclidean space ${\mathbb {R}}^n$, this viewpoint is consistent (i.e. coincides) with the usual definition (see Remark 2.4).

Moreover, also the spectral fractional Laplacian expression has an interpretation on noncompact manifolds since actually it is not needed that the spectrum is discrete. Indeed, for every (possibly) noncompact manifold *M* we can regard $(-\Delta )$ as a densely defined, nonnegative, essentially self-adjoint unbounded operator on $L^2(M)$. Then, by the spectral theorem, there exists a unique *spectral resolution* *E* of $(-\Delta )$. That is, an operator-valued measure

$$\begin{aligned} E: \big \{ \text {Borel subsets of }[0, +\infty ) \big \} \rightarrow \big \{\text {Bounded linear operators on } L^2(M) \big \} \end{aligned}$$

supported on the spectrum $\sigma (-\Delta )\subset [0,+\infty )$ of $(-\Delta )$ (which can, in general, be non-discrete) such that, for every $u\in \textrm{Dom}(-\Delta )$ and $v\in L^2(M)$

$$\begin{aligned} \langle -\Delta u, v \rangle _{L^2(M)} = \int \limits _0^\infty \lambda \, d\langle E_\lambda u, v \rangle =\int \limits _{\sigma (-\Delta )} \lambda \, d\langle E_\lambda u, v \rangle . \end{aligned}$$

Actually $E_\lambda$ is a projector, that is a non-negative and self-adjoint operator with $E_\lambda ^2=E_\lambda$, for every $\lambda \in [0, +\infty )$. Then, the spectral fractional Laplacian is defined by the spectral theorem as

59 $$\begin{aligned} (-\Delta )^{s/2}_\textrm{Spec} u:= \int \limits _0^\infty \lambda ^{s/2} \, d E_\lambda u , \end{aligned}$$

for every *u* in its natural domain

$$\begin{aligned} u \in \textrm{Dom}( (-\Delta )^{s/2}_\textrm{Spec}) = \left\{ v \in L^2(M) \,\, \Big | \,\, \int \limits _0^\infty \lambda ^s \, d \Vert E_\lambda v \Vert ^2 < +\infty \right\} . \end{aligned}$$

This formula coincides with definition (58) we gave for closed manifolds since in the case of closed manifolds, the spectrum $\sigma (-\Delta )$ is discrete, and the spectral measure *E* is supported on the eigenvalues.

Moreover, it is important to notice that the spectral formula (59) coincides, essentially always (meaning on the natural function space where both formulas make sense and the integrals converge), with the one we gave in (7) using Bochner’s integrals. Indeed, for every *u* that makes the integral in (7) convergent in the sense of Bochner, then $u\in \textrm{Dom}((-\Delta )^{s/2}_\textrm{Spec})$ and the two Laplacians coincide $(-\Delta )^{s/2}_\textrm{Spec} u = (-\Delta )^{s/2}_{B} u$. Making a complete and precise proof of this is beyond the scope of this work, but the proof is essentially as follows. Let $u\in \textrm{Dom}((-\Delta )^{s/2}_\textrm{Spec})$ so that

$$\begin{aligned} \int \limits _0^\infty \lambda ^s \, d \Vert E_\lambda u \Vert ^2 < +\infty , \end{aligned}$$

and recall formula (6). Then (see Section A.5.4 in [19] to justify all the steps)

$$\begin{aligned} \Vert (-\Delta )^{s/2}_\textrm{Spec} u \Vert _{L^2(M)}^2&= \int \limits _0^\infty \lambda ^s \, d \Vert E_\lambda u \Vert ^2 \\ {}&= \int \limits _0^\infty \left( \frac{1}{\Gamma (-s/2)} \int \limits _0^\infty (e^{-\lambda t}-1)\frac{dt}{t^{1+s/2}} \right) ^2 \, d \Vert E_\lambda u \Vert ^2 \\ {}&= \left\| \frac{1}{\Gamma (-s/2)} \int \limits _0^\infty (e^{ t \Delta } u - u )\frac{dt}{t^{1+s/2}} \right\| _{L^2(M)}^2 = \Vert (-\Delta )^{s/2}_{B} u \Vert _{L^2(M)}^2 . \end{aligned}$$

From here, using that both $(-\Delta )^{s/2}_\textrm{Spec}$ and $(-\Delta )^{s/2}_{B}$ are self-adjoint one can depolarize the last identity of the norms to get $(-\Delta )^{s/2}_\textrm{Spec} u = (-\Delta )^{s/2}_{B} u$ in $L^2(M)$.

Moreover, it was proved in [13] that they also coincide, on a very general class of functions, with $(-\Delta )^{s/2}_\textrm{Si} u$ on every stochastically complete Riemannian manifold.

Regarding definition (iii), via the extension problem, it still generalizes well in the case of some non-compact manifolds. Some extra assumptions are needed in order to establish the equivalence between (i) and (iii)—see [3] for a related discussion concerning the definition of the fractional Laplacian on noncompact manifolds.

It will be clear from our proofs that, in the case of noncompact manifolds for which the equivalence of (i) and (iii) can be established, the fractional Sobolev spaces $H^{s/2}$ will enjoy the same properties as the ones established here in the case of compact manifolds (e.g. the monotonicity formula), with almost identical proofs.

### Monotonicity formula for stationary points of semilinear elliptic functionals and *s*-minimal surfaces

The monotonicity formula for minimizing *s*-minimal surfaces in ${\mathbb {R}}^n$ was proved in the seminal article [8], and for Allen–Cahn type critical points, it was first obtained in [7]. In [20], the monotonicity formula is shown to extend to stationary *s*-minimal surfaces. Here, we prove the analogous (local) monotonicity formula on a Riemannian manifold. The proof holds simultaneously for any *s*-minimal surface, that is, for any stationary point of the fractional perimeter regardless of second variation or regularity, and also for any stationary point of a semilinear elliptic functional with a nonnegative potential term, hence including the fractional Allen–Cahn energy. For $r>0$ and $p \in M$ denote

60 $$\begin{aligned} \begin{aligned} B_r(p)&= \big \{ q \in M \, : \, d_g(q, p)< r \big \} \,, \\ \widetilde{B}_{r}^+(p, 0)&= \big \{ (q,z) \in \widetilde{M} \, : \, d_{\widetilde{g}}((q,z), (p, 0) ) < r \big \} \,, \\ \partial \widetilde{B}_{r}^+(p, 0)&= \partial \left( \widetilde{B}_{r}^+(p, 0) \right) \, \\ \partial ^+ \widetilde{B}_{r}^+(p, 0)&= \partial \widetilde{B}_{r}^+(p, 0) \cap \{ z>0 \} \,. \end{aligned} \end{aligned}$$

In all this section, since there will be no possible ambiguity, we will use $\nabla$ instead of ${{\widetilde{\nabla }}}$ to denote the gradient in ${\widetilde{M}}$ with respect to the product metric.

#### Theorem 3.4

(Monotonicity formula) Let $(M^n, g)$ be an *n*-dimensional, closed Riemannian manifold. Let $s \in (0,2)$ and

$$\begin{aligned} {\mathcal {E}}(v)=[v]^2_{H^{s/2}(M)}+\int \limits _M F(v) \, dV, \end{aligned}$$

where *F* is any smooth nonnegative function. Let $u:M\rightarrow {\mathbb {R}}$ be stationary for ${\mathcal {E}}$ under inner variations, meaning that ${\mathcal {E}}(u)<\infty$ and for any smooth vector field *X* on *M* there holds $\frac{d}{dt}\big |_{t=0} {\mathcal {E}}(u\circ \psi _X^t)=0$, where $\psi _X^t$ is the flow of *X* at time *t*. For $(p_\circ ,0) \in \widetilde{M}$ and $R>0$ define

$$\begin{aligned} \Phi (R):= \frac{1}{R^{n-s}} \left( \frac{\beta _s}{2}\int \limits _{\widetilde{B}^+_R(p_\circ ,0)} z^{1-s}| \nabla U(p,z)|^2 \, dV_p dz + \int \limits _{B_R(p_\circ )} F(u) \, dV \right) , \end{aligned}$$

where *U* is the unique solution given by Theorem 2.25. Then, there exist constants $C=C(n)$ and $R_\textrm{max}=R_\textrm{max}(M, p_\circ )>0$ with the following property: whenever $R_\circ \le R_\textrm{max}$ and *K* is an upper bound for all the sectional curvatures of *M* in $B_{R_\circ }(p_\circ )$, then

$$\begin{aligned} R \mapsto \Phi (R)e^{C \sqrt{K} R } \hspace{7pt}\textit{is non-decreasing for} \hspace{7pt}R < R_\circ , \end{aligned}$$

and the inequality

$$\begin{aligned} \Phi '(R)&\ge - C \sqrt{K} \Phi (R)+\frac{ s }{R^{n-s+1}} \int \limits _{B_R(p_\circ )} F(u) \, dV\\&\quad + \frac{2\beta _s}{R^{n-s}} \int \limits _{\partial ^+ \widetilde{B}_R^+(p_\circ ,0)} z^{1-s} \langle \nabla U , \nabla d \rangle ^2 \, d\,{\widetilde{\sigma }} \end{aligned}$$

holds for all $$R < R_0 $$, with $$d(\cdot ) = d_{\widetilde{g}}((p_\circ ,0), \,\cdot \, )$$ the distance function on $$\widetilde{M}$$ from the point $$(p_\circ , 0)$$.

Moreover, in the particular case where $M={\mathbb {R}}^n$, $F\equiv 0$, $s\in (0,1)$, and $u=\chi _E-\chi _{E^c}$ is a stationary set for the fractional *s*-perimeter, there holds

$$\begin{aligned} \Phi '(R) = \frac{\beta _{s}}{2}{R^{n-s}} \int \limits _{\partial ^+ \widetilde{{\mathcal {B}}}_R^+(p_\circ ,0)} z^{1-s} \langle \nabla U , \nabla d \rangle ^2 \, dxdz \ge 0, \end{aligned}$$

which shows that $\Phi$ is nondecreasing and that it is constant if and only if *E* is a cone.

#### Remark 3.5

It will follow from the proof that the radius $R_\textrm{max}$ in Theorem 3.4 can be taken to be $R_\textrm{max} = \text {inj}_{M}(p_\circ )/4$. Moreover, since *M* is compact $R_\textrm{max}$ is uniformly bounded below as $R_\textrm{max} (M,p_\circ ) \ge \text {inj}_M/4$, for all $p_\circ \in M$.

Before proving the monotonicity formula of Theorem 3.4, we will need two preliminary lemmas from Riemannian geometry, which will allow us to bound the “Riemannian errors" in two formulas regarding the distance function.

#### Lemma 3.6

Let $(M^n,g)$ be an *n*-dimensional Riemannian manifold, $p\in M$, $R_0 < \text {inj}_M(p)$ and let *K* be an upper bound for all the sectional curvatures in $B_{R_0}(p)$. Denote by *d* the distance function to the point *p*. Then, for all $R<\min \{ R_0, \tfrac{1}{\sqrt{K}}\}$ there holds in $B_R(p)$:

$$\begin{aligned} |\langle \nabla _{V}(d\nabla d),V\rangle - |V|^2| \le \sqrt{K}R|V|^2 , \end{aligned}$$

for every vector field *V* on *M*.

#### Proof

We can compute

$$\begin{aligned} \langle V, \nabla _{V} (d\nabla d) \rangle&= \langle V, \langle V,\nabla d\rangle \nabla d \rangle + d \langle V, \nabla _{V} (\nabla d) \rangle \\ {}&= \langle V , \nabla d \rangle ^2+ d \, \nabla ^2 d(V , V ). \end{aligned}$$

On the other hand, the Hessian Comparison theorem—see Lemma 7.1 in [14]—gives that

$$\begin{aligned} | d \, \nabla ^2 d (V,V)- |V- \langle V, \nabla d \rangle \nabla d|^2| \le d \sqrt{K} |V|^2 \end{aligned}$$

in $B_R(p)$, whenever $R < \min \{\text {inj}_{M}(p), \frac{1}{\sqrt{K}}\}$. Moreover, since $|\nabla d|^2 =1$, we also have that

$$\begin{aligned} |V- \langle V, \nabla d \rangle \nabla d|^2 = |V|^2-2 \, \langle V, \nabla d \rangle ^2 + \langle V, \nabla d \rangle ^2 |\nabla d|^2 = |V|^2- \langle V, \nabla d \rangle ^2 . \end{aligned}$$

Hence

$$\begin{aligned} | d \, \nabla ^2 d (V,V)+ \langle V, \nabla d \rangle ^2 - |V|^2 | \le d \sqrt{K} |V|^2 \le R\sqrt{K}|V|^2 \end{aligned}$$

holds in $B_R(p)$, as long as $R < \min \{ R_0, \frac{1}{\sqrt{K}}\}$, and this conludes the proof. $\square$

#### Lemma 3.7

Let $$(M^n,g)$$ be an *n*-dimensional Riemannian manifold, $$p\in M$$, $$R_0 < \text {inj}_M(p)$$ and let *K* be an upper bound for all the sectional curvatures in $$B_{R_0}(p)$$. Then, there exists $$ C=C(n) > 0 $$ such that, for all $$ R < R_0$$, in $$B_R(p)$$ we have that

$$\begin{aligned} |\text {div}(d\nabla d)-n| \le C K R^2 . \end{aligned}$$

#### Proof

Fix $p\in M$, and denote $d(p,\cdot )$ just by $d(\cdot )$. Observe first that every geodesic $\sigma$ with $\sigma (0)=p$ and contained in $B_{R_0}(p)$ is uniquely minimizing. For any $R< R_0$ and $x \in B_{R}(p)$, let $\gamma : [0,d ] \rightarrow M$ be the normalized geodesic with $\gamma (0)=p$ and $\gamma (d) = x$. Note also that

$$\begin{aligned} \text {div}(d\nabla d)= |\nabla d|^2 + d \Delta d = 1 +d\Delta d . \end{aligned}$$

Consider $${\dot{\gamma }}(d) \in T_{x}M$$, and complete it to an orthonormal basis $$ \{e_1:={\dot{\gamma }}(d), e_2, \dotsc , e_n \}$$ of $$T_x M $$. For $$i=2, 3, \dotsc ,n$$, let $$\gamma _i $$ be the geodesic with $$\gamma _i(0)=x$$ and $${\dot{\gamma }}_i(0)=e_i$$. We can compute

$$\begin{aligned} \Delta d(x) = \sum _{i=1}^n \nabla ^2 d(x)(e_i, e_i) = \sum _{i=1}^n \frac{d^2}{ds^2}\bigg |_{s=0} (d \circ \gamma _i ) = \sum _{i=2}^n \frac{d^2}{ds^2}\bigg |_{s=0} (d \circ \gamma _i ), \end{aligned}$$

where we have used that $\frac{d^2}{ds^2}\bigg |_{s=0} (d \circ \gamma ) = \frac{d^2}{ds^2}\bigg |_{s=0} (d(x) +s )=0$.

Let $J_i$ be the Jacobi field along $\gamma$ with $J_i(0)=0$ and $J_i(d)=e_i$, well defined by uniqueness of geodesics between endpoints. Denote by

$$\begin{aligned} I(X,Y)= \int \limits _0^d \langle D_t X, D_t Y \rangle - \text {Rm}({\dot{\gamma }}, X, {\dot{\gamma }}, Y) \, dt \end{aligned}$$

the index form associated to $\gamma$ on [0, *d*]. Since $\gamma$ is minimizing along all curves with the same endpoints, for every vector field *X* on $\gamma ([0,d])$ orthogonal to ${\dot{\gamma }}$ and with $X(0)=0$ and $X(d)=e_i$ we must have

$$\begin{aligned} 0 \le I(J_i-X, J_i-X) = I(J_i,J_i) -2I(J_i,X) + I(X,X) \,. \end{aligned}$$

Since $J_i$ is a Jacobi field, one can easily check that $I(J_i,X)=I(J_i,J_i)$, hence $I(J_i, J_i) \le J(X,X)$. Take $X(t)=\frac{t}{d}E_i(t)$, where $E_i(t)$ is the parallel transport of $e_i \in T_x M$ along $\gamma$. From the second variation formula for arc length we get

$$\begin{aligned} \frac{d^2}{ds^2}\bigg |_{s=0} (d \circ \gamma _i )&= \int \limits _{0}^d |D_t J_i|^2 - \text {Rm}({\dot{\gamma }}, J_i, {\dot{\gamma }}, J_i) \, dt = I(J_i, J_i) \\ {}&\le I(X,X) = \int \limits _{0}^d |D_t X|^2 - \text {Rm}({\dot{\gamma }}, X, {\dot{\gamma }}, X) \, dt \\ {}&\le \int \limits _{0}^d |D_t X|^2 + K|X|^2 \, dt \,, \end{aligned}$$

where we have used that $\sup _{p\in B_{R_0}}|\text {Sec}_p| \le K$. Thus

$$\begin{aligned} \frac{d^2}{ds^2}\bigg |_{s=0} (d \circ \gamma _i ) \le \int \limits _{0}^d |D_t X|^2 + K|X|^2 \, dt = \int \limits _{0}^d \frac{1}{d^2}+K\frac{t^2}{d^2} \, dt = \frac{1}{d} \left( 1+ K\frac{d^2}{3} \right) . \end{aligned}$$

Hence

$$\begin{aligned} d \Delta d = \sum _{i=2}^n \frac{d^2}{ds^2}\bigg |_{s=0} (d \circ \gamma _i ) \le n-1+ K\frac{n-1}{3}d^2 \,, \end{aligned}$$

or equivalently

$$\begin{aligned} |\text {div}(d\nabla d)(x) -n | = |d(x)\Delta d(x) +1 -n | \le K\frac{n-1}{3}d^2 \le K\frac{n-1}{3} R^2 , \end{aligned}$$

and this completes the proof with $C(n)=\frac{n-1}{3}>0$. $\square$

We can now prove the monotonicity formula.

#### Proof of Theorem 3.4

Since during the entire proof, the point $p_\circ \in M$ will be fixed, we will not specify the center of the balls in what follows, as this will always be $(p_\circ ,0)$ for balls inside $\widetilde{M}$ and $p_\circ$ for balls on *M*. We divide the proof into two steps.

**Step 1.** First, we show that if *u* is stationary for the energy ${\mathcal {E}}(v)=[v]^2_{H^{{s/2}}(M)}+\int _M F(v)$ under inner variations, then its Caffarelli–Silvestre extension *U* is stationary for the energy

$$\begin{aligned} U \mapsto \frac{\beta _s}{2} \int \limits _{{\widetilde{M}}} z^{1-s} |\nabla U|^2 \, dVdz+\int \limits _{M}F(U|_{M})\,dV , \end{aligned}$$

under inner variations on ${\widetilde{M}}$ given by vector fields *Y* on ${\widetilde{M}}$ such that $Y|_M$ is tangent to *M*.

Recall that the Caffarelli–Silvestre extension of *u* is given by (41).

Let *Y* be a vector field on ${\widetilde{M}}$ such that $Y|_{M}$ is tangent to *M*, and let $\psi _Y^t$ denote its flow at time *t*. Let also $V_{t}$ be the Caffarelli–Silvestre extension of $u\circ \psi _{Y|_{M}}^{t}$, for any $t\in {\mathbb {R}}$. By the minimality of the extension in the energy space, we have

$$\begin{aligned}&\frac{d}{dt}\Big |_{t=0} \frac{\beta _s}{2} \int \limits _{{\widetilde{M}}} z^{1-s} |\nabla (U\circ \psi _Y^{-t})|^2 \\&\quad = \lim _{t\rightarrow 0} \frac{1}{t}\left( \frac{\beta _s}{2} \int \limits _{{\widetilde{M}}} z^{1-s} |\nabla U|^2- \frac{\beta _s}{2} \int \limits _{{\widetilde{M}}} z^{1-s} |\nabla (U\circ \psi _Y^{t})|^2 \right) \\&\quad \le \lim _{t\rightarrow 0} \frac{1}{t}\left( \frac{\beta _s}{2}\int \limits _{{\widetilde{M}}} z^{1-s} |\nabla U|^2- \frac{\beta _s}{2} \int \limits _{{\widetilde{M}}} z^{1-s} |\nabla V_t|^2 \right) \\&\quad =\lim _{t\rightarrow 0} \frac{ [u]^2_{H^{{s/2}}(M)}-[ u\circ \psi _Y^{t}]^2_{H^{{s/2}}(M)}}{t} =\frac{d}{dt}\Big |_{t=0} [ u\circ \psi _Y^{-t}]^2_{H^{{s/2}}(M)} \,, \end{aligned}$$

and likewise

$$\begin{aligned}&\frac{d}{dt}\Big |_{t=0} \frac{\beta _s}{2}\int \limits _{{\widetilde{M}}} z^{1-s} |\nabla (U\circ \psi _Y^{-t})|^2\\&\quad = \lim _{t\rightarrow 0}\frac{1}{t}\left( \frac{\beta _s}{2} \int \limits _{{\widetilde{M}}} z^{1-s} |\nabla (U\circ \psi _Y^{-t})|^2- \frac{\beta _s}{2}\int \limits _{{\widetilde{M}}} z^{1-s} |\nabla U|^2\right) \\&\quad \ge \lim _{t\rightarrow 0} \frac{1}{t}\left( \frac{\beta _s}{2} \int \limits _{{\widetilde{M}}} z^{1-s} |\nabla V_{-t}|^2- \frac{\beta _s}{2} \int \limits _{{\widetilde{M}}} z^{1-s} |\nabla U|^2\right) \\&\quad =\lim _{t\rightarrow 0} \frac{ [u\circ \psi _Y^{-t}]^2_{H^{{s/2}}(M)}-[u]^2_{H^{{s/2}}(M)}}{t}\\&\quad =\frac{d}{dt}\Big |_{t=0} [ u\circ \psi _Y^{-t}]^2_{H^{{s/2}}(M)} \,. \end{aligned}$$

Hence

$$\begin{aligned} \frac{d}{dt}\Big |_{t=0} \frac{\beta _s}{2} \int \limits _{{\widetilde{M}}} z^{1-s} |\nabla (U\circ \psi _Y^{-t})|^2=\frac{d}{dt}\Big |_{t=0} [ u\circ \psi _Y^{-t}]^2_{H^{{s/2}}} \, . \end{aligned}$$

Since *u* is stationary for the energy ${\mathcal {E}}(v)=[v]^2_{H^{{s/2}}(M)}+\int _M F(v) \,dV$ under inner variations, this shows that *U* is stationary for the energy $U \mapsto \frac{\beta _s}{2} \int _{{\widetilde{M}}} z^{1-s} |\nabla U|^2 \, dVdz+\int _{M}F(U|_{M}) \,dV$ under inner variations on ${\widetilde{M}}$, with vector fields *Y* as above, and this concludes the first step.

**Step 2.** We now compute such an inner variation for a suitably chosen *Y*. First, the variation of the potential part of the energy is

61 $$\begin{aligned} \frac{d}{dt} \Big |_{t=0}\int \limits _M F(u\circ \psi _Y^{-t}) \, dV&=\frac{d}{dt} \Big |_{t=0}\int \limits _M F(u)J_t(p) \, dV_p\nonumber \\&=\int \limits _M F(u)\text {div}_g (Y|_M) \, dV . \end{aligned}$$

The quantity $\text {div}_g(Y|_M)$ will be estimated later. We now focus on computing the variation for the Sobolev part of the energy. Once again, we change variables in the integral using the flow $\psi _Y^t$, obtaining

62 $$\begin{aligned} \int \limits _{{\widetilde{M}}} z^{1-s} |\nabla (U\circ \psi _Y^{-t})|^2 \, dVdz&=\int \limits _{{\widetilde{M}}} (z\circ \psi _Y^t )^{1-s} |\nabla (U\circ \psi _Y^{-t})|^2 \circ \psi _Y^{t} \,J_t(p,z)\,dV_pdz \,. \end{aligned}$$

Now, we choose the vector field *Y*. We take $Y=\eta (d) d\nabla d$, where $d=d_{{\widetilde{g}}}((p_\circ , 0), \, \cdot \,)$ is the distance on ${\widetilde{M}}$ from the point $(p_\circ ,0)$ and $\eta =\eta _\delta$ is a single variable smooth function with $\eta \equiv 1$ on [0, *R*], decreasing to zero on $[R,R+\delta ]$, and $\eta \equiv 0$ on $[R+\delta ,+\infty )$. Since the distance $d_{{\widetilde{g}}}((p_\circ , 0), \, \cdot \,)$ restricts to the distance $d_g(p_\circ , \, \cdot \,)$ on *M* when computed on points on ${\widetilde{M}}$ with $z=0$, clearly $Y|_M$ is tangent to *M*. We want to exchange the order of derivation and integration in (62). Hence, we compute separately the three terms that will appear in doing so. For the first term, using that $d_{{\widetilde{g}}}^2((p,z), (p_\circ ,0)) = d_g^2(p, p_\circ )+z^2$ and the definition of *Y* we see that

$$\begin{aligned} \frac{d}{dt}\left| _{t=0}(z\circ \psi _Y^{t})^{1-s}=(1-s)z^{-s}\eta (d) z=(1-s)z^{1-s}\eta (d)\,\right. . \end{aligned}$$

As for the second term that will appear, a simple general computation—see for example the lines after Lemma 3.1 in [17]—shows that

$$\begin{aligned} \frac{d}{dt}\Big |_{t=0}|\nabla (U\circ \psi _Y^{-t})|^2(\psi _Y^{t}(x))=-2\langle \nabla _{\nabla U}Y,\nabla U\rangle \,. \end{aligned}$$

Moreover, using the form chosen for *Y* we have that

$$\begin{aligned} \langle \nabla _{\nabla U}Y,\nabla U\rangle&=\langle \nabla _{\nabla U}(\eta (d) d\nabla d),\nabla U\rangle \\&=\langle \nabla U,\nabla \eta (d)\rangle \langle d\nabla d,\nabla U\rangle +\eta (d)\langle \nabla _{\nabla U}(d\nabla d),\nabla U\rangle \\&=\eta '(d)\langle \nabla U,\nabla d\rangle \langle d\nabla d,\nabla U\rangle +\eta (d)\langle \nabla _{\nabla U}(d\nabla d),\nabla U\rangle \\&=d\eta '(d)|\langle \nabla U,\nabla d\rangle |^2+\eta (d)\langle \nabla _{\nabla U}(d\nabla d),\nabla U\rangle \, . \end{aligned}$$

Notice that *K* is also an upper bound for all the sectional curvatures on $\widetilde{M}$ in $\widetilde{B}_{R_\circ }(p_\circ ,0)$ and that $\text {inj}_{M}(p_\circ )=\text {inj}_{\widetilde{M}}(p_\circ ,0)$. Thus, by Lemma 3.6 applied to $V=\nabla U$,

$$\begin{aligned} \langle \nabla _{\nabla U}Y,\nabla U\rangle&=d\eta '(d)|\langle \nabla U,\nabla d\rangle |^2+\eta (d)(1+O(\sqrt{K}R))|\nabla U|^2 \end{aligned}$$

for all $R< \min \left\{ R_\circ , \tfrac{1}{\sqrt{K}} \right\}$. Lastly, for the remaining factor in the integral, Lemma (3.7) gives that

$$\begin{aligned} \frac{d}{dt}\Big |_{t=0} J_t ={{\widetilde{\text {div}}}} (Y)&=\eta '(d)d|\nabla d|^2 + \eta (d){{\widetilde{\text {div}}}} (d\nabla d) \\&= d\eta '(d)+\eta (d)(n+1)(1+O(\sqrt{K}R))\, , \end{aligned}$$

in $$B_R(p_\circ )$$, for $$R<\min \left\{ R_\circ , \tfrac{1}{\sqrt{K}} \right\} $$.

Now, analogously applying Lemma (3.7) on *M* instead of ${\widetilde{M}}$ to (61), we already find an estimation for the potential energy:

$$\begin{aligned} \frac{d}{dt} \Big |_{t=0}\int \limits _M F(u\circ \psi _Y^{-t}) \, dV&=\int \limits _M F(u)\big (d\eta '(d)+\eta (d)n(1+O(\sqrt{K}R))\big ) \, dV\, . \end{aligned}$$

Moreover, it follows from (the local version of) Bonnet-Myers’ theorem that $R_\circ< R_\textrm{max}:= \text {inj}_{M}(p_\circ )/4 < \min \left\{ \text {inj}_{M}(p_\circ ), \tfrac{1}{\sqrt{K}} \right\}$, and this will be our final choice of $R_\textrm{max}$ for the statement. From now on, we always consider $R<R_\circ \le R_\textrm{max}=\text {inj}_{M}(p_\circ )/4$.

Regarding the Sobolev part of the energy, exchanging differentiation and integration and substituting the estimates we have obtained so far gives:

$$\begin{aligned} \frac{d}{dt}\Big |_{t=0}&\int \limits _{{\widetilde{M}}} z^{1-s} |\nabla (U\circ \psi _Y^{-t})|^2 \\&\quad = \int \limits _{{\widetilde{M}}} (1-s)z^{1-s}\eta (d)|\nabla U|^2 + z^{1-s} \big (-2d\eta '(d)|\langle \nabla U, \nabla d \rangle |^2\\&\qquad -2 \eta (d)(1+O(\sqrt{K}R))|\nabla U|^2 \big ) \\&\qquad + \int \limits _{{\widetilde{M}} }z^{1-s} |\nabla U |^2 \big (d\eta '(d)+\eta (d)(n+1)(1+O(\sqrt{K}R)) \big ) \, dVdz \\ {}&= (n-s)(1+O(\sqrt{K}R))\int \limits _{{\widetilde{B}}_{R+\delta }^+ } z^{1-s} |\nabla U|^2 \eta (d)\\&\qquad + \int \limits _{ {{\widetilde{B}}_{R+\delta }^+ } \setminus {{\widetilde{B}}_{R}^+ } } z^{1-s} d\eta '(d) \big ( |\nabla U|^2-2|\langle \nabla U, \nabla d \rangle |^2 \big ) . \end{aligned}$$

Adding the expressions for the potential and Sobolev parts of the energy, we get

$$\begin{aligned} \frac{d}{dt}\bigg |_{t=0} \bigg ( \frac{\beta _s}{2} \int \limits _{{\widetilde{M}}} z^{1-s}&|\nabla (U\circ \psi _Y^{-t})|^2 + \int _M F(u\circ \psi _Y^{-t}) \bigg ) \\ {}&=(n-s) (1+O(\sqrt{K}R)) \frac{\beta _s}{2} \int \limits _{{\widetilde{B}}_{R+\delta }^+ } \eta (d) z^{1-s}|\nabla U|^2\\&\hspace{0.4cm}+ \frac{\beta _s}{2} \int \limits _{{{\widetilde{B}}_{R+\delta }^+ } \setminus {{\widetilde{B}}_{R}^+ }} d\eta '(d) z^{1-s}(|\nabla U|^2-2 \langle \nabla U,\nabla d\rangle ^2 )\\&\hspace{0.4cm}+n(1+O(\sqrt{K}R))\int \limits _{B_{R+\delta }} \eta (d) F(u)+\int _{B_{R+\delta } \setminus B_R} d\eta '(d) F(u) \,. \end{aligned}$$

By stationarity of *u* and Step 1 we know that the left-hand side is equal to 0 for every *Y*, thus the right-hand side vanishes for all $\eta = \eta _\delta$ defined as above. Since this holds for all $\delta >0$, we now let $\delta \searrow 0$ so that $\eta _\delta$ converges to the characteristic function of [0, *R*]. This gives (for a.e. $R\in (0, R_\circ )$)

$$\begin{aligned} 0&=(n-s)(1+O(\sqrt{K}R)) \frac{\beta _s}{2} \int \limits _{{\widetilde{B}} ^+_R} z^{1-s}|\nabla U|^2-R \frac{\beta _s}{2}\int \limits _{\partial {\widetilde{B}} ^+_R} z^{1-s}|\nabla U|^2\\&\quad +2R\frac{\beta _s}{2}\int \limits _{\partial ^+ {\widetilde{B}} ^+_R} (\partial _\nu U)^2\\&\quad +n(1+O(\sqrt{K}R))\int _{B_R} F(u)-R\int \limits _{\partial B_R} F(u). \end{aligned}$$

Rearranging the terms and multiplying by $R^{-n+s-1}$, we deduce that

$$\begin{aligned} -\frac{(n-s)}{R^{n-s+1}}&\left( \frac{\beta _s}{2} \int \limits _{{\widetilde{B}}^+_R} z^{1-s }|\nabla U|^2+ \int \limits _{B_R} F(u) \right) \\&\qquad + \frac{1}{R^{n-s}} \left( \frac{\beta _s}{2} \int \limits _{\partial {\widetilde{B}}^+_R} z^{1-s }|\nabla U|^2 + \int \limits _{\partial B_R} F(u) \right) \\&\quad \ge -\frac{C\sqrt{K}}{R^{n-s}} \left( \frac{\beta _s}{2} \int \limits _{{\widetilde{B}}^+_R} z^{1-s }|\nabla U|^2 + \int \limits _{B_R} F(u) \right) \\&\qquad +\frac{\beta _s}{R^{n-s}}\int \limits _{\partial ^+ {\widetilde{B}}^+_R} z^{1-s} \langle \nabla U,\nabla d\rangle ^2 +\frac{s}{R^{n-s+1}} \int _{B_R} F(u) \,, \end{aligned}$$

for some absolute constant $C > 0$. In other words,

$$\begin{aligned} \Phi '(R) \ge - C \sqrt{K} \Phi (R) + \frac{ \beta _{s}}{R^{n-s}} \int \limits _{\partial ^+ \widetilde{B} _R^+ } z^{1-s} \langle \nabla U,\nabla d\rangle ^2 +\frac{ s }{R^{n-s+1}} \int \limits _{B_R} F(u) \, dV \,, \end{aligned}$$

and this implies, in particular, that

$$\begin{aligned} \frac{d}{dR} \Big (e^{C\sqrt{K}R} \Phi (R)\Big )\ge 0 \quad \text{ for } \text{ all } \hspace{7pt}R< R_\circ . \end{aligned}$$

Lastly, in the case where $M={\mathbb {R}}^n$, $F\equiv 0$, $s\in (0,1)$ and $u=\chi _E-\chi _{E^c}$ is a stationary set for the fractional *s*-perimeter, instead of the two bounds used above

$$\begin{aligned} \langle \nabla _{\nabla U}(d\nabla d),\nabla U\rangle&=(1+O(\sqrt{K}R))|\nabla U|^2 \,, \\ {\widetilde{\text {div}}}(d\nabla d)&= (n+1)(1+O(\sqrt{K}R)) \,, \end{aligned}$$

given respectively by Lemmas 3.6 and 3.7, one has the equalities

$$\begin{aligned} \langle \nabla _{\nabla U}(d\nabla d),\nabla U\rangle _{{\mathbb {R}}^{n+1}}&=|\nabla U|^2 \,, \\ \text {div}_{{\mathbb {R}}^{n+1}}(d\nabla d)&= n+1 \,, \end{aligned}$$

where *U* is the extension of $u=\chi _E-\chi _{E^c}$. Thus, following the proof one finds the exact expression

$$\begin{aligned} \Phi '(R) = \frac{\beta _{s}}{R^{n-s}} \int \limits _{\partial ^+ \widetilde{{\mathcal {B}}} _R^+ (p_\circ ,0)} z^{1-s} \langle \nabla U,\nabla d\rangle ^2 \, dxdz \ge 0 . \end{aligned}$$

In particular, $\Phi$ is constant if and only if $\langle \nabla U,\nabla d\rangle =0$, that is, if and only if *E* is dilation-invariant for dilations with center at $p_\circ \in {\mathbb {R}}^n$. With this, we conclude the proof. $\square$

### The fractional Sobolev energy under inner variations

We next study how the fractional Sobolev energy behaves under inner variations. For this, we need first to study how the singular kernel $K_s$ behaves when translating its arguments under the flow of a vector field.

#### Proposition 3.8

Let (*M*, *g*) be a closed *n*-dimensional Riemannian manifold and $s\in (0,2)$. Consider any smooth vector field $X \in {\mathfrak {X}}( M)$, and fix points $p,q\in M$. Writing $\psi ^t$ for the flow of *X* at time *t*, then the kernel satisfies

63 $$\begin{aligned} \bigg | \frac{d^\ell }{dt^\ell }\bigg |_{t=0} K_s(\psi ^t(p),\psi ^t(q)) \bigg |\le C(1 +K_s(p,q)). \end{aligned}$$

for some constant $C=C(M,s,\ell , \max _{0\le k\le \ell } \Vert \nabla ^k X\Vert _{L^\infty (M)} )$ which stays bounded for *s* away from 0 and 2.

#### Proof

This follows from the estimates of Theorem 2.13, in particular by (19) and (20). We prove the result just for $\ell =1$, as the general case just follows by induction by the very same arguments. Let $R=R(M)>0$ be such that the flatness assumption $\textrm{FA}_\ell (M,g,16R, p, \varphi _p)$ holds for every $p\in M$; such an *R* exists by Remark 2.10. We split in two cases.

**Case 1:** $$q \in \varphi _p({\mathcal {B}}_{4R}(0))$$.

In this case, denoting $K(x,y):= K_s(\varphi _p(x), \varphi _p(y))$ and $k(x, z): = K(x,x+z)$ as in Theorem 2.13, we have that

$$\begin{aligned} K_s(\psi ^t\circ \varphi _p(x),\psi ^t\circ \varphi _p(y)) = K(\psi _p^t(x), \psi _p^t(y)) = k (\psi _p^t(x), \psi _p^t(y)-\psi _p^t(x)), \end{aligned}$$

where $\psi _p^t$ is the flow of $\xi = (\varphi _p)^* X$, i.e. the vector field $\xi =\xi _p\in {\mathfrak {X}} ({\mathcal {B}}_{16R}(0))$ such that $X\circ \varphi _p = (\varphi _p)_* \xi$. Then, for all $x,y\in {\mathcal {B}}_{2R}(0)$ we have:

$$\begin{aligned} \frac{d}{dt}\bigg |_{t=0} K(\psi _p^t(x) , \psi _p^t(y))&= \frac{d}{dt}\bigg |_{t=0} k ( \psi _p^t(x), \psi _p^t(y)-\psi _p^t(x)) \\ {}&= \frac{\partial k}{\partial x^\alpha }(x,y-x) \xi ^\alpha (x) + \frac{\partial k}{\partial z^\alpha }(x,y-x) (\xi ^\alpha (x) - \xi ^\alpha (y) ) \,, \end{aligned}$$

where sum over repeated indices is assumed. Hence, by (19) of Theorem 2.13 we get

$$\begin{aligned} \left| \frac{d}{dt}\bigg |_{t=0} K(\psi _p^t(x) , \psi _p^t(y)) \right|&\le \frac{C}{|y-x|^{n+s}} \Vert \xi \Vert _{L^{\infty }} + \frac{C}{|y-x|^{n+s+1}} \Vert D \xi \Vert _{L^{\infty }}|y-x| \\ {}&\le \frac{C}{|y-x|^{n+s}} \le C K(x,y) \end{aligned}$$

for some $C=C(n,s, \Vert \xi \Vert _{C^{0,1}})$, where in the last line we have also used Lemma 2.20. Finally, evaluating this inequality at $x=0$ and $y= \varphi _p^{-1}(q)$ we obtain

$$\begin{aligned} \left| \frac{d}{dt}\bigg |_{t=0} K(\psi ^t(p), \psi ^t(q))\right| = \left| \frac{d}{dt}\bigg |_{t=0} K(\psi _p^t(0), \psi _p^t(y)) \right| \le C K(0,y) = C K_s(p,q), \end{aligned}$$

as wanted.

**Case 2:** $q \notin \varphi _p({\mathcal {B}}_{4R}(0))$. Then $\textrm{FA}_\ell (M,g,R,q, \varphi _q)$ holds and the sets $\varphi _p({\mathcal {B}}_{R}(0))$ and $\varphi _q({\mathcal {B}}_{R}(0))$ are disjoint. Hence, by Proposition 2.21 the kernel $K_{pq}(x,y): = K_s(\varphi _p(x), \varphi _q(y))$ is smooth (with uniform estimates on all derivatives) in the domain ${\mathcal {B}}_{R/2}(0)\times {\mathcal {B}}_{R/2}(0)$. Hence

$$\begin{aligned} \frac{d}{dt}\bigg |_{t=0} K_s(\psi ^t\circ \varphi _p(x),\psi ^t\circ \varphi _q(x))&= \frac{d}{dt}\bigg |_{t=0} K_{pq}(\psi _p^t(x),\psi _p^t(y)) \\ {}&= \frac{\partial K_{pq}}{\partial x^{\alpha }} (x,y) \xi _p^{\alpha }(x) + \frac{\partial K_{pq}}{\partial y^{\alpha }} (x,y) \xi _q^{\alpha }(y) . \end{aligned}$$

Using Proposition 2.21 to bound the derivatives of $K_{pq}$, and then evaluating at $(x,y)=(0,0)$ gives

$$\begin{aligned} \left| \frac{d}{dt}\bigg |_{t=0} K_s(\psi ^t(p),\psi ^t(q)) \right| \le \frac{C}{R^{n+s}} , \end{aligned}$$

for some $$C=C(n,s, \Vert \xi _p \Vert _{L^{\infty }}, \Vert \xi _q \Vert _{L^{\infty }})$$.

Putting together the two cases above, we get

$$\begin{aligned} \left| \frac{d}{dt}\bigg |_{t=0} K_s(\psi ^t(p),\psi ^t(q)) \right| \le C(1+K_s(p,q)) , \end{aligned}$$

for some $C=C(M, n, s, \Vert X \Vert _{C^1(M)})$ and conclude the proof. $\quad \square$

We also record a version of Proposition 3.8, which depends only on local quantities:

#### Proposition 3.9

Let (*M*, *g*) be a closed *n*-dimensional Riemannian manifold and $s\in (0,2)$. Assume that the flatness assumption $\textrm{FA}_\ell (M,g,R,p,\varphi )$ holds, and let $X\in {\mathfrak {X}}( M)$ be a smooth vector field supported on $\varphi ({\mathcal {B}}_{R/4})$. Writing $\psi ^t$ for the flow of *X* at time *t*, then for every $x,y \in {\mathcal {B}}_{R/4}(0)$ we have

64 $$\begin{aligned} \bigg | \frac{d^\ell }{dt^\ell }\bigg |_{t=0} K_s(\psi ^t(\varphi (x)),\psi ^t(\varphi (y))) \bigg |\le C K_s(\varphi (x), \varphi (y)) \le C\frac{\alpha _{n,s}}{|x-y|^{n+s}}, \end{aligned}$$

for some constant $C=C(n,s, \Vert X \Vert _{C^\ell (\varphi ({\mathcal {B}}_R))})$. Moreover, given $T>0$ we have that, for all $0\le t \le T$,

65 $$\begin{aligned} \bigg | \frac{d^\ell }{dt^\ell } K_s(\psi ^t(\varphi (x)),\psi ^t(\varphi (y))) \bigg |\le C_T K_s(\varphi (x), \varphi (y)) \le C_T\frac{\alpha _{n,s}}{|x-y|^{n+s}}, \end{aligned}$$

where $$C_T=C_T(n,s, T, \Vert X \Vert _{C^\ell (\varphi ({\mathcal {B}}_{R/4}))})$$.

The constants stay bounded for *s* away from 0 and 2.

#### Proof

By scaling, we can assume $R=1$. The second inequality in both (64) and (65) then follows from Lemma 2.20. As for the first inequality of (64), it follows from the proof of Case 1 in Proposition 3.8, since it only depends on local estimates for *X*. Finally, (65) can be deduced from (64). Indeed, note that for all $1 \le k \le \ell$ and $0\le t\le T$,

66 $$\begin{aligned} \left| \frac{d^k}{d t^k} K_s(\psi ^t(\varphi (x)),\psi ^t(\varphi (y))) \right|{} & {} = \left| \frac{d^k}{d r^k} \bigg |_{r=0} K_s(\psi ^{t+r}(\varphi (x)),\psi ^{t+r}(\varphi (y))) \right| \nonumber \\{} & {} \le C_0 K_s(\psi ^t(\varphi (x)),\psi ^t(\varphi (y))) , \end{aligned}$$

with $C_0=C_0(n,s, \Vert X \Vert _{C^\ell (\varphi ({\mathcal {B}}_1))})$. Thus, we are only left with proving that

$$\begin{aligned} K_s(\psi ^t(\varphi (x)),\psi ^t(\varphi (y))) \le C_T K_s(\varphi (x),\varphi (y)) \end{aligned}$$

for some $C_T=C_T(n,s, T, \Vert X \Vert _{C^\ell (\varphi ({\mathcal {B}}_1))})$. But this follows itself from (66), with $k=1$, since we can write the inequality as

$$\begin{aligned} \frac{d}{dt} \big [e^{-C_0t}K_s(\psi ^t(\varphi (x)),\psi ^t(\varphi (y)))\big ]\le 0\,, \end{aligned}$$

and integrating we find that

$$\begin{aligned} K_s(\psi ^t(\varphi (x)),\psi ^t(\varphi (y))) \le e^{C_0 T} K_s(\varphi (x),\varphi (y)) \end{aligned}$$

for every $$0\le t \le T$$. $$\square $$

Proposition 3.8 can be used to bound time derivatives of the energy of “flown objects” by their energy at time zero. We show this for the fractional Sobolev energy:

#### Lemma 3.10

Let $s \in (0,2)$ and $v\in H^{s/2}(M)$ be a function with $|v|\le 1$. Let $X \in {\mathfrak {X}} (M)$ be a smooth vector field and $v_t:= v \circ \psi ^{-t}$, where $\psi ^t$ is the flow of *X* at time *t*. Then, for all $T>0$ there holds

$$\begin{aligned} \sup _{0<t<T} \bigg | \frac{d^\ell }{d t^\ell }{\mathcal {E}}_{M}(v_t) \bigg |\le C\big (1+ {\mathcal {E}}_{M}(v)\big )\,, \end{aligned}$$

for some constant $C=C(M,s,\ell ,T, \max _{0\le k\le \ell } \Vert \nabla ^k X\Vert _{L^\infty (M)} )$ which stays bounded for *s* away from 0 and 2.

#### Proof

Let *C* denote a constant that depends only on *M*, *s*, $\ell$, *T* and $\max _{0\le k\le \ell } \Vert \nabla ^k X\Vert _{L^\infty (M)}$.

The idea of the proof is to change variables using the flow $\psi ^t$ in the corresponding integrals defining the Allen-Cahn energy, and after that to exchange integration and differentiation.

Let us start with the Sobolev part of the energy. We have, denoting by $J_t$ the Jacobian of the flow:

67 $$\begin{aligned} \frac{d^\ell }{d t^\ell }{\mathcal {E}}^\textrm{Sob}_{M}({v_t})&=\frac{d^\ell }{d t^\ell }\int \!\int |v(\psi ^{- t}(p))-v(\psi ^{- t}(q))|^2K_s(p,q) \, dV_p\, dV_q\nonumber \\&=\frac{d^\ell }{d t^\ell }\int \!\int |v(p)-v(q)|^2 K_s(\psi ^{ t}(p),\psi ^{ t}(q))\,J_{ t}(p)J_{ t}(q) \, dV_p\, dV_q \nonumber \\&=\int \!\int |v(p)-v(q)|^2\frac{d^\ell }{d t^\ell }\Big [K_s(\psi ^{ t}(p),\psi ^{ t}(q)) \,J_{ t}(p)J_{ t}(q)\Big ] dV_p\, dV_q. \end{aligned}$$

Since $0< t < T$, the derivatives in time of the Jacobians $J_t$ can of course be bounded by a constant *C* with the right dependencies. What remains in order to bound (67) by $C(1+{\mathcal {E}}^\textrm{Sob}_M(v))$ is to control the first *k*-th derivatives in time of $K_s(\psi ^{ t}(p),\psi ^{ t}(q))$ by $C (1+K_s(p,q))$, for all $0<t<T$. The main bound is given by Proposition 3.8, which gives for all $1 \le k \le \ell$:

68 $$\begin{aligned}{} & {} \left| \frac{d^k}{d t^k} K_s(\psi ^{ t}(p),\psi ^{ t}(q)) \right| = \left| \frac{d^k}{d r^k} \bigg |_{r=0} K_s(\psi ^{ t+r}(p),\psi ^{ t+r}(q)) \right| \nonumber \\{} & {} \quad \le C \big (1+K_s(\psi ^{ t}(p),\psi ^{ t}(q))\big ). \end{aligned}$$

Now, integrating this inequality for $k=1$ similarly to how we proceeded in the proof of Lemma 3.9, we conclude that

69 $$\begin{aligned} K_s(\psi ^{ t}(p),\psi ^{ t}(q))\le C (1+K_s(p,q)),\hspace{7pt}\text { for all } 0< t<T\,. \end{aligned}$$

We can now go back to (67) and apply the bounds that we just derived. We get that

$$\begin{aligned} \left| \frac{d^\ell }{d t^\ell }{\mathcal {E}}^\textrm{Sob}_{M}(v_t) \right|&\le \int \!\int |v(p)-v(q)|^2\frac{d^\ell }{d t^\ell }\Big [K_s(\psi ^{ t}(p),\psi ^{ t}(q)) \,J_{ t}(p)J_{ t}(q)\Big ] dV_p\, dV_q \\&\le C \int \!\int |v(p)-v(q)|^2 (1+K_s(p,q)) \, dV_p dV_q\\&= C(1+{\mathcal {E}}^\textrm{Sob}_{M}(v)) \end{aligned}$$

for all $0< t<T$, where *C* has the right dependencies.

The potential part of the energy is simpler to deal with. Indeed, we have

70 $$\begin{aligned} \frac{d^\ell }{d t^\ell }{\mathcal {E}}^\textrm{Pot}_{M}({v_t}) =\frac{d^\ell }{d t^\ell }\int \varepsilon ^{-s} W(v(\psi ^{- t}(p)) dV_p\, = \int \varepsilon ^{-s} W(v(p))\frac{d^\ell }{d t^\ell }\,J_{ t}(p) dV_p,\nonumber \\ \end{aligned}$$

from which we directly conclude that

$$\begin{aligned} \left| \frac{d^\ell }{d t^\ell }{\mathcal {E}}^\textrm{Pot}_{M}({v_t}) \right| \le C{\mathcal {E}}^\textrm{Pot}_{M}({v}), \end{aligned}$$

finishing the proof. $\square$

Lemma 3.10 has a local version, which comes from applying local estimates for the kernel instead.

#### Lemma 3.11

Let *M* satisfy the flatness assumptions $\textrm{FA}_\ell (N,g,p,R,\varphi )$. Let $s \in (0,2)$ and $v\in H^{s/2}(M)$ be a function with $|v|\le 1$. Let $X \in {\mathfrak {X}} (M)$ be a smooth vector field supported on $\varphi ({\mathcal {B}}_{R/2})$, and put $v_t:= v \circ \psi _X^{-t}$, where $\psi _X^t$ is the flow of *X* at time *t*. Then, for all $T>0$ there holds

$$\begin{aligned} \sup _{0< t<T} \left| \frac{d^\ell }{d t^\ell }{\mathcal {E}}_{\varphi ({\mathcal {B}}_{R/2})}(v_{t}) \right| \le C(1+{\mathcal {E}}_{\varphi ({\mathcal {B}}_{R/2})}(v))\,, \end{aligned}$$

for some constant $C=C(s,\ell ,T, \max _{0\le k\le \ell } \Vert \nabla ^k X\Vert _{L^\infty (\varphi ({\mathcal {B}}_{R/2}))} )$ which stays bounded for *s* away from 0 and 2.

#### Proof

We modify the proof of Lemma 3.10 accordingly. First, by scaling, it suffices to prove the Lemma in the case $R=1$. Since *X* is supported on $\varphi ({\mathcal {B}}_{1/2})$, the integrand in (67) is supported then on

$$\begin{aligned}&(N\times N)\setminus (\varphi ({\mathcal {B}}_{1/2})^c\times \varphi ({\mathcal {B}}_{1/2})^c)\\&\quad =\Big [(\varphi ({\mathcal {B}}_{2/3})\times \varphi ({\mathcal {B}}_{2/3}))\setminus (\varphi ({\mathcal {B}}_{1/2})^c\times \varphi ({\mathcal {B}}_{1/2})^c)\Big ]\cup \\&\qquad \cup \Big [(\varphi ({\mathcal {B}}_{1/2})\times (N\setminus \varphi ({\mathcal {B}}_{2/3})))\cup ((N\setminus \varphi ({\mathcal {B}}_{2/3}))\times \varphi ({\mathcal {B}}_{1/2}))\Big ]\, , \end{aligned}$$

so that

$$\begin{aligned} \bigg |&\frac{d^k}{d t^k} {\mathcal {E}}_{M}^\textrm{Sob}(v_{t})\bigg |=\bigg |\frac{d^k}{d t^k}{\mathcal {E}}_{\varphi ({\mathcal {B}}_{1/2})}^\textrm{Sob}(v_{t})\bigg |\\&=\bigg |\int \!\int _{(\varphi ({\mathcal {B}}_{2/3})\times \varphi ({\mathcal {B}}_{2/3}))\setminus (\varphi ({\mathcal {B}}_{1/2})^c\times \varphi ({\mathcal {B}}_{1/2})^c)} |v(p)\\&\qquad -v(q)|^2\frac{d^k}{d t^k}\Big [K(\psi _Y^{ t}(p),\psi _Y^t(q))J_{ t}(p)J_{ t}(q)\Big ]\,dV_p\,dV_q \\&\qquad +2\int \!\int _{\varphi ({\mathcal {B}}_{1/2})\times (N\setminus \varphi ({\mathcal {B}}_{2/3}))} |v(p)-v(q)|^2\frac{d^k}{d t^k}\Big [K(\psi _Y^{ t}(p),q)J_{ t}(p)\Big ]\,dV_p\,dV_q\bigg | \\&\le C\int \!\int _{({\mathcal {B}}_{2/3}\times {\mathcal {B}}_{2/3})\setminus ({\mathcal {B}}_{1/2}^c\times {\mathcal {B}}_{1/2}^c)} |v(\varphi (x))\\&\qquad -v(\varphi (y))|^2\Big |\frac{d^k}{d t^k}\Big [K(\varphi (\psi _X^{ t}(x)),\varphi (\psi _X^{ t}(y)))J_{ t}(\varphi (x))J_{ t}(\varphi (y))\Big ]\Big |\,dx\,dy\\&\qquad +C\int \!\int _{{\mathcal {B}}_{1/2}\times (N\setminus \varphi ({\mathcal {B}}_{2/3}))} |v(\varphi (x))-v(q)|^2\Big |\frac{d^k}{d t^k}\Big [K(\varphi (\psi _X^{ t}(x)),q)J_{ t}(\varphi (x))\Big ]\Big |\,dx\,dV_q\, . \end{aligned}$$

Bounding the derivatives in time of the Jacobians by a constant with the right dependencies, using (64) to bound the kernel in the first double integral, and using (21) to bound the integral in *q* in the second double integral by a constant, we conclude that

$$\begin{aligned} \left| \frac{d^k}{d t^k}{\mathcal {E}}_{\varphi ({\mathcal {B}}_{1/2})}^\textrm{Sob}(v_{t})\right| \le C(1+{\mathcal {E}}_{\varphi ({\mathcal {B}}_{1/2})}^\textrm{Sob}(v))\,. \end{aligned}$$

Regarding the potential part of the energy, from the computation in (70) we readily find that

71 $$\begin{aligned} \left| \frac{d^\ell }{d t^\ell }{\mathcal {E}}^\textrm{Pot}_{\varphi ({\mathcal {B}}_{1/2})}({v_t}) \right| \le C{\mathcal {E}}^\textrm{Pot}_{\varphi ({\mathcal {B}}_{1/2})}({v}) \end{aligned}$$

where *C* has the right dependencies, which completes the proof. $\square$

### Estimates for the extension problem

#### Lemma 3.12

Let $s\in (0,2)$ and *M* satisfy the flatness assumption $\textrm{FA}_2(M,g,2,p,\varphi )$. Let also $U: \widetilde{B}^+_2(p,0) \rightarrow {\mathbb {R}}$ be any function solving

$$\begin{aligned} \widetilde{\text {div}}(z^{1-s} {{\widetilde{\nabla }}} U) =0 \hspace{7pt}in \hspace{7pt}\widetilde{B}^+_2(p,0) , \end{aligned}$$

and let *u* be its trace on $B_2(p)$. Assume also $U \in L^{\infty }(\widetilde{B}^+_2(p,0))$ and $u=0$ in $B_{3/2}(p)$. Then there exists $C=C(n)>0$ such that

$$\begin{aligned} z^{1-s}| {{\widetilde{\nabla }}} U (q,z) | \le \frac{C}{s} \Vert U \Vert _{L^\infty ({\widetilde{B}}^+_{6/5}(p,0) )} . \end{aligned}$$

for every $$(q,z) \in {\widetilde{B}}_1^+(p,0)$$.

#### Proof

Let *C* denote a constant that depends only on *n*. This estimate is proved by a barrier argument. Let $\alpha , \beta >0$ to be chosen later, and for $q_\circ \in B_{11/10}(p)$ define

$$\begin{aligned} b_{q_\circ }(q,z):= \frac{\alpha }{2} \big |\varphi ^{-1}(q) - \varphi ^{-1}(q_\circ ) \big |^2- \beta ( z^2 - 2 z^s) . \end{aligned}$$

Denote by $\Delta _g$ the Laplace–Beltrami operator of (*M*, *g*). Then, by $\textrm{FA}_2(M,g,2,p,\varphi )$, for $(x,z) \in {\widetilde{B}}_{6/5}^+$ there holds $| \Delta _g b_{q_\circ } (q,z)| \le C$. Moreover

$$\begin{aligned} \big ( \partial _{zz} +\tfrac{1-s}{z} \partial _z \big )z^2 =2s \hspace{7pt}\hspace{7pt}\text {and} \hspace{7pt}\hspace{7pt}\big ( \partial _{zz} +\tfrac{1-s}{z} \partial _z \big )z^s =0 . \end{aligned}$$

Hence

$$\begin{aligned} {{\widetilde{\text {div}}}} (z^{1-s} {{\widetilde{\nabla }}} b_{q_\circ } ) = z^{1-s} \left( \Delta _g b_{q_\circ } + \partial _{zz} b_{q_\circ } + \frac{1-s}{z} \partial _z b_{q_\circ } \right) \le z^{1-s}(C \alpha - 2 s \beta ) \le 0 , \end{aligned}$$

provided we take $\beta = C \alpha /s$.

Since $U =0$ in $B_{3/2}(p) \times \{0\}$ clearly $|U| \le b_{q_\circ }(\cdot ,0)$ in $B_{6/5}(p) \times \{0\}$. Moreover, for every $(x,z) \in \partial ^+ {\widetilde{B}}^+_{6/5}(p,0)$ there holds

$$\begin{aligned} b_{q_\circ }(q,z) \ge C\alpha - \beta ( z^2-2 z^s) \ge C\alpha \ge \Vert U \Vert _{L^\infty ({\widetilde{B}}^+_{6/5}(p,0) )} \ge U(x,z) , \end{aligned}$$

provided we choose $\alpha = C\Vert U \Vert _{L^\infty ({\widetilde{B}}^+_{6/5}(p,0) ) }$.

Hence, with this choice of $\alpha$ and $\beta$, $|U| \le b_{q_\circ }$ on the full boundary $\partial {\widetilde{B}}_{6/5}^+(p,0)$. Since also *U* solves $\widetilde{\text {div}}(z^{1-s} {{\widetilde{\nabla }}} U) =0$ in ${\widetilde{B}}_{6/5}^+(p,0)$, by the maximum principle we get

72 $$\begin{aligned} |U (q_\circ ,z)| \le b_{q_\circ }(q_\circ ,z) \le \frac{Cz^s}{s} \Vert U \Vert _{L^\infty ({\widetilde{B}}^+_{6/5}(p,0))}, \end{aligned}$$

for $(q_\circ ,z) \in {\widetilde{B}}^+_{11/10}(p,0)$. Moreover, by standard (interior) gradient estimates for uniformly elliptic equations, for all $(q,z) \in {\widetilde{B}}^+_1(p,0)$ we have

$$\begin{aligned} | {{\widetilde{\nabla }}} U (q,z) | \le \Vert {{\widetilde{\nabla }}} U \Vert _{L^{\infty }({\widetilde{B}}^+_{z/100}(q,z))} \le \frac{C}{z} \Vert U\Vert _{L^{\infty }({\widetilde{B}}^+_{z/50}(q,z))} , \end{aligned}$$

which, since ${\widetilde{B}}^+_{z/50}(q,z) \subset {\widetilde{B}}^+_{11/10}(p,0)$, together with (72) implies

$$\begin{aligned} | {{\widetilde{\nabla }}} U (x,z) | \le \frac{C}{s} z^{s-1} \Vert U \Vert _{L^\infty ({\widetilde{B}}^+_{6/5})} , \end{aligned}$$

and this concludes the proof. $\square$

#### Lemma 3.13

Let $s_0 \in (0,2)$, $s \in (s_0,2)$. Consider the Riemannian manifold $({\mathbb {R}}^n,g)$ with $\big (1-\tfrac{1}{100}\big ) |v|^2 \le g_{ij}(x)v^i v^j \le \big (1+\tfrac{1}{100}\big )|v|^2$ and $\Vert g_{ij}\Vert _{C^{1,1}({{\overline{{\mathbb {R}}}}}^n)} \le 1$. Let also $u:{\mathbb {R}}^n\rightarrow [-1,1]$ and $U: {\mathbb {R}}^{n}\times {\mathbb {R}}_+ \rightarrow [-1,1]$ be the extension of *u* (in the sense of Theorem 2.25). Then

73 $$\begin{aligned} \int \limits _{{\mathcal {B}}_{1}^+(0,0)} |{{\widetilde{\nabla }}} U|^2 z^{1-s}dVdz \le C \int \!\int \limits _{{\mathbb {R}}^n\times {\mathbb {R}}^n \setminus ({\mathcal {B}}_2^c \times {\mathcal {B}}_2^c)} \frac{|u(x) - u(y)|^2}{|x-y|^{n+s}} \,dxdy, \end{aligned}$$

with *C* depending only on *n* and $s_0$.

#### Proof

We proceed as in [8, Proposition 7.1]. Assume without loss of generality that $\int _{{\mathcal {B}}_2}u(x) dx=0.$ Let $\xi : {\mathbb {R}}^n \rightarrow {\mathbb {R}}$ be a cutoff function such that $\xi = 1$ in ${\mathcal {B}}_{3/2}(0)$ and it is compactly supported in ${\mathcal {B}}_2(0)$. We write $u= u \xi + u(1-\xi )= u_1 + u_2$ and $U= U_1+ U_2$.

On the one hand, since $u_1$ is compactly supported we have

$$\begin{aligned} \begin{aligned}&\beta _s \int \limits _{{\mathbb {R}}^{n+1}_+}z^{1-s} | {{\widetilde{\nabla }}} U_1|^2 dVdz = \Vert u_1\Vert _{H^{s/2}({\mathbb {R}}^n,g)} \\&\quad = \int \!\int \limits _{{\mathbb {R}}^n\times {\mathbb {R}}^n\setminus ({\mathcal {B}}_2^c\times {\mathcal {B}}_2^c)} |u_1(x) - u_1(y)|^2 K(x,y) \;dV_x dV_y\\&\quad \le C \alpha _{n,s} \int \!\int \limits _{{\mathcal {B}}_2\times {\mathbb {R}}^n} \frac{|u(x) - u(y)|^2}{|x-y|^{n+s}} \xi ^2(x) \,dxdy \\&\quad + C \alpha _{n,s} \int \!\int \limits _{{\mathcal {B}}_2\times {\mathbb {R}}^n} |u(x)|^2 \frac{|\xi (x) - \xi (y)|^2}{|x-y|^{n+s}} \,dxdy\\&\quad \le C \alpha _{n,s}\int \!\int \limits _{{\mathbb {R}}^n\times {\mathbb {R}}^n \setminus ({\mathcal {B}}_2^c \times {\mathcal {B}}_2^c)} \frac{|u(x) - u(y)|^2}{|x-y|^{n+s}} \,dxdy + C \int \limits _{{\mathcal {B}}_2} |u(x)|^2 dx. \end{aligned} \end{aligned}$$

Moreover, using the fractional Poincaré inequality (recall $\int _{{\mathcal {B}}_2}u(x) dx=0$):

$$\begin{aligned}{} & {} \int \limits _{{\mathcal {B}}_2} |u(x)|^2 dx \le C\alpha _{n,s}\int \!\int \limits _{{\mathcal {B}}_2\times {\mathcal {B}}_2} \frac{|u(x) - u(y)|^2}{|x-y|^{n+s}} \,dxdy\\{} & {} \quad \le C\alpha _{n,s}\int \!\int \limits _{{\mathbb {R}}^n\times {\mathbb {R}}^n \setminus ({\mathcal {B}}_2^c \times {\mathcal {B}}_2^c)} \frac{|u(x) - u(y)|^2}{|x-y|^{n+s}} \,dxdy =: \textrm{I} \end{aligned}$$

On the other hand (using again $\int _{{\mathcal {B}}_2}u(y) dy=0$)

$$\begin{aligned}\int \limits _{{\mathbb {R}}^n}\frac{u(x)^2}{(1+|x|^2)^{\frac{n+s}{2}}}dx= & {} \int \!\int \frac{(u(x)-u(y))^2}{(1+|x|^2)^{\frac{n+s}{2}}}\frac{\chi _{{\mathcal {B}}_2}(y)}{|{\mathcal {B}}_2|} dxdy\\{} & {} - \int \!\int \frac{u(y)^2}{(1+|x|^2)^{\frac{n+s}{2}}}\frac{\chi _{{\mathcal {B}}_2}(y)}{|{\mathcal {B}}_2|}dxdy \le C\textrm{I},\end{aligned}$$

and by Holder inequality

$$\begin{aligned} \int \limits _{{\mathbb {R}}^n}\frac{|u(x)|}{(1+|x|^2)^{\frac{n+s}{2}}}dx \le \left( \int \limits _{{\mathbb {R}}^n} \frac{|u(x)|^2}{(1+|x|^2)^{\frac{n+s}{2}}} dx \right) ^{1/2} \left( \int \limits _{{\mathbb {R}}^n} \frac{1}{(1+|x|^2)^{\frac{n+s}{2}}} dx \right) ^{1/2} \le C \textrm{I}^{1/2}. \end{aligned}$$

**Claim.** There is $C=C(n)>0$ such that for $(x,z) \in {\mathcal {B}}_1^+$

74 $$\begin{aligned} z^{1-s} |{{\widetilde{\nabla }}} U_2(x,z)| \le C \int \limits _{{\mathbb {R}}^n}\frac{|u_2(y)|}{{(1+|y|^2)^{\frac{n+s}{2}}}}dy . \end{aligned}$$

We postpone the proof of this claim and first see how to conclude the proof of Lemma 3.13 with it.

By the claim, if $(x,z) \in {\mathcal {B}}_1^+$ then

$$\begin{aligned}z^{1-s} |{{\widetilde{\nabla }}} U_2(x,z)| \le C \int \limits _{{\mathbb {R}}^n}\frac{|u_2(y)|}{{(1+|y|^2)^{\frac{n+s}{2}}}}dy \le C \int \limits _{{\mathbb {R}}^n}\frac{|u(y)|}{{(1+|y|^2)^{\frac{n+s}{2}}}}dy \le C \textrm{I}^{1/2}\end{aligned}$$

But then the inequality

$$\begin{aligned} \int \limits _{{\mathcal {B}}_1^+}z^{1-s} |{{\widetilde{\nabla }}} U_2|^2 dxdz\le & {} C\textrm{I}^{1/2} \int \limits _{{\mathcal {B}}_1^+} |{{\widetilde{\nabla }}} U_2| dxdz\\\le & {} C\textrm{I}^{1/2} \left( \,\int \limits _{{\mathcal {B}}_1^+} z^{1-s}|{{\widetilde{\nabla }}} U_2|^2 dxdz\right) ^{1/2} \left( \,\int \limits _{{\mathcal {B}}_1^+} z^{s-1} dxdz\right) ^{1/2}, \end{aligned}$$

gives

$$\begin{aligned} \int \limits _{{\mathcal {B}}_1^+}z^{1-s} |{{\widetilde{\nabla }}} U_2|^2 dxdz \le C\textrm{I}, \end{aligned}$$

and the lemma follows.

It only remains to prove (74). Let $H_N(x,y,t)$ be the heat kernel of $N:=({\mathbb {R}}^n,g)$. By (40) the fractional Poisson kernel^6^${\mathbb {P}}_N: {\mathbb {R}}^n \times {\mathbb {R}}^n \times (0,\infty ) \rightarrow [0, \infty )$ of *N* can be represented as

$$\begin{aligned} {\mathbb {P}}_N(x,y,z)= \frac{z^{s}}{2^s\Gamma (s/2)} \int \limits _{0}^{\infty } H_N(x,y,t) e^{-\frac{z^2}{4t}}\frac{dt}{t^{1+s/2}}, \end{aligned}$$

and the solution $U_2$ to the extension problem with trace $u_2$ is $U_2(x,z) = \int _{{\mathbb {R}}^n} {\mathbb {P}}_N(x,y,z) u_2(y) \, dV_y$. Then, by Lemma 2.14 we have that ${\mathbb {P}}_N$ is comparable (up to dimensional constants) to the fractional Poisson kernel of ${\mathbb {R}}^n$ with its standard metric, that is

$$\begin{aligned} cs \frac{z^s}{(|x-y|^2+z^2)^{\frac{n+s}{2}}} \le {\mathbb {P}}_N(x,y,z) \le Cs \frac{z^s}{(|x-y|^2+z^2)^{\frac{n+s}{2}}} , \end{aligned}$$

for some $C,c>0$ dimensional. Hence, for every $(x,z) \in {{\widetilde{{\mathcal {B}}}}}_{6/5}^+$

$$\begin{aligned} |U_2 (x,z)| \le Cs \int \limits _{{\mathbb {R}}^n} \frac{|u_2(y)|}{(|x-y|^2+z^2)^{\frac{n+s}{2}}} \, dy \le Cs \int \limits _{{\mathbb {R}}^n \setminus {\mathcal {B}}_2} \frac{|u_2(y)|}{|x-y|^{n+s}} \, dy . \end{aligned}$$

Since $x\in {\mathcal {B}}_{6/5}$ and $y\in {\mathbb {R}}^n {\setminus } {\mathcal {B}}_2$ there holds $|x-y|\ge \frac{1}{100}\sqrt{1+|y|^2}$, and hence

$$\begin{aligned} \Vert U_2\Vert _{L^\infty ({\mathcal {B}}^+_{6/5})} \le Cs \int \limits _{{\mathbb {R}}^n} \frac{|u_2(y)|}{(1+|y|^2)^{\frac{n+s}{2}}} \, dy . \end{aligned}$$

From here, the result follows directly by Lemma 3.12. $\square$

We will now give an interpolation result for the extended energy. We will use the following standard interpolation result on a Euclidean ball:

#### Proposition 3.14

Let $s\in (0,1)$, and let $u:{\mathcal {B}}_1\subset {\mathbb {R}}^n\rightarrow [-1,1]$ be a function of bounded variation. Then,

$$\begin{aligned} \int \!\int _{{\mathcal {B}}_1\times {\mathcal {B}}_1} \frac{|u(x)-u(y)|^2}{|x-y|^{n+s}}\,dx\,dy \le \frac{C(n)}{(1-s)s}[u]_{BV({\mathcal {B}}_1)}^s\Vert u\Vert _{L^1({\mathcal {B}}_1)}^{1-s}\,. \end{aligned}$$

#### Proof

See for instance Proposition 4.2 in [5] for a simple proof; see also [6]. $\square$

#### Lemma 3.15

Let $s_0\in (0,1)$ and $s \in (s_0,1)$. Let *M* satisfy flatness assumptions $\textrm{FA}_1(M,g,1,p,\varphi )$. Let also $U: \widetilde{B}^+_1(p,0) \rightarrow (-1,1)$ be any function solving

75 $$\begin{aligned} \widetilde{\text {div}}(z^{1-s} {{\widetilde{\nabla }}} U) =0\,, \end{aligned}$$

and let *u* be its trace on $B_1(p)$. Then for all $\varrho >0$, $R\ge 1$, $k \in {\mathbb {R}}$ and $q\in B_{1/2}(p)$ such that $B_{R\varrho }(q)\subset B_{3/4}(p)$,

$$\begin{aligned}{} & {} \varrho ^{s-n} \beta _s \int \limits _{{\widetilde{B}}^+_{\varrho }(q,0)} z^{1-s} |{{\widetilde{\nabla }}} U|^2\,dVdz\\{} & {} \quad \le \frac{C}{R^s} + \frac{C}{1-s}\left( \varrho ^{-n}\int \limits _{B_{R \varrho }(q)}|u + k|\,dV\right) ^{1-s} \left( \varrho ^{1-n}\int \limits _{B_{R \varrho }(q)} |\nabla u|\,dV\right) ^s, \end{aligned}$$

where the constant *C* depends only on *n* and $s_0$.

#### Proof

Let us show that if *U* and $U'$ are two different solutions $\widetilde{B}^+_1(p,0) \rightarrow (-1,1)$ of (75) with the same trace *u* on $B_{3/4}(p)$, then

76 $$\begin{aligned} \int \limits _{{\widetilde{B}}^+_{\varrho }(q,0)} z^{1-s} \big (|{{\widetilde{\nabla }}} U|^2-|{{\widetilde{\nabla }}} U'|^2\big )\,dVdz \le C\int \limits _{{\widetilde{B}}^+_{\varrho }(q,0)} z^{1-s} |{{\widetilde{\nabla }}} U'|^2\,dVdz. \end{aligned}$$

Indeed, by Lemma 3.12 (rescaled) we have $z^{1-s} \big |{{\widetilde{\nabla }}} (U-U')\big | \le C$ in ${\widetilde{B}}^+_{1/2}(p,0)$. Thus, we obtain

$$\begin{aligned} \begin{aligned}&\int \limits _{{\widetilde{B}}^+_{\varrho }(q,0)} z^{1-s} \big (|{{\widetilde{\nabla }}} U|^2-|{{\widetilde{\nabla }}} U'|^2\big )\,dVdz \\&\quad = \int \limits _{{\widetilde{B}}^+_{\varrho }(q,0)} z^{1-s} \big ({{\widetilde{\nabla }}} (U-U')\big )\cdot \big ( {{\widetilde{\nabla }}}(U+U'))\,dVdz \\&\quad \le C \left( \int \limits _{{\widetilde{B}}^+_{\varrho }(q,0)} z^{s-1} dVdz\right) ^{\frac{1}{2}} \left( \int \limits _{{\widetilde{B}}^+_{\varrho }(q,0)} z^{1-s} \big (|{{\widetilde{\nabla }}} U|^2+|{{\widetilde{\nabla }}} U'|^2 \big ) dVdz\right) ^{\frac{1}{2}}\\&\quad \le \frac{1}{2} \int \limits _{{\widetilde{B}}^+_{\varrho }(q,0)} z^{1-s} \big (|{{\widetilde{\nabla }}} U|^2-|{{\widetilde{\nabla }}} U'|^2\big )\,dVdz + C\int \limits _{{\widetilde{B}}^+_{\varrho }(q,0)} z^{1-s} |{{\widetilde{\nabla }}} U'|^2\,dVdz \end{aligned} \end{aligned}$$

Thus (76) follows.

Now let $g_{ij}$ be the components of the metric in the coordinates $\varphi ^{-1}$, $\eta \in C^\infty _c({\mathcal {B}}_1)$ be a nonnegative smooth cut-off function satisfying $\eta \equiv 1$ in ${\mathcal {B}}_{3/4}$, and put $g'_{ij} = g_{ij}\eta + \delta _{ij}(1-\eta )$, a metric defined in the whole ${\mathbb {R}}^n$. Thanks to (76) it is enough to prove the lemma for the manifold $({\mathbb {R}}^n, g')$ with $p=0$ and with *U* replaced by the (unique!) bounded solution $U'$ of (75) (with respect to the metric $g'$) in all of ${\mathbb {R}}^n\times {\mathbb {R}}_+$. But in this case we can use Lemma 3.13 (rescaled) and obtain

$$\begin{aligned} \begin{aligned} \varrho ^{s-n} \int \limits _{{\widetilde{B}}^+_{\varrho }} z^{1-s} |{{\widetilde{\nabla }}} U'|^2\,dVdz&\le C \varrho ^{s-n}\int \!\int \limits _{({\mathbb {R}}^n\times {\mathbb {R}}^n) \setminus ({\mathcal {B}}_{2\varrho }^c\times {\mathcal {B}}_{2\varrho }^c)} \frac{|u(x)-u(y)|^2}{|x-y|^{n+s}} dxdy\\&\le C \varrho ^{s-n} 2\int \!\int \limits _{ {\mathcal {B}}_{R\varrho }\times {\mathcal {B}}_{R\varrho } \, \cup \, {\mathcal {B}}_{\varrho }\times {\mathcal {B}}_{R\varrho }^c} \frac{|u(x)-u(y)|^2}{|x-y|^{n+s}} dxdy \\&\le C \varrho ^{s-n} \bigg ( \int \!\int _{ {\mathcal {B}}_{R\varrho }\times {\mathcal {B}}_{R\varrho } } \frac{|u(x)-u(y)|^2}{|x-y|^{n+s}} dxdy + \frac{C \varrho ^{n-s}}{R^s}\bigg ) , \end{aligned} \end{aligned}$$

where we have used that

$$\begin{aligned} \int \!\int \limits _{{\mathcal {B}}_\varrho \times {\mathcal {B}}_{R\varrho }^c} \frac{|u(x)-u(y)|^2}{|x-y|^{n+s}} \, dxdy \le C \int _{{\mathcal {B}}_\varrho }\left( \int \limits _{{\mathcal {B}}_{R\varrho }} \frac{1}{(|y|-\varrho )^{n+s}} dy\right) dx \le \frac{C\varrho ^{n}}{(R\varrho )^{s}} \,. \end{aligned}$$

We conclude using the interpolation inequality of Proposition 3.14, since the modulus of the Euclidean gradient in ${\mathbb {R}}^{n+1}$ and the metric gradient $|{{\widetilde{\nabla }}} U|$ are comparable. $\square$

## Data Availability

This paper does not use any data set.

## Footnotes

1 As customary, by heat kernel here we mean the fundamental solution of the heat equation $\partial _t u = \Delta u$ on *M*, where $\Delta$ denotes the Laplace–Beltrami operator on *M*.

2 Notice that for functions taking values in $\{\pm 1\}$ the potential part of the energy vanishes and the Sobolev part of the energy gives the fractional perimeter.

3 Note that $$\frac{1}{|\Gamma (-s/2)|}= \frac{s/2}{\Gamma (1-s/2)}$$.

4 That is $\varphi _p = (\exp _p\circ \, i)|_{{\mathcal {B}}_{R_0}(0)}$ for any isometric identification of $i: {\mathbb {R}}^n \rightarrow TM_p$

5 That is, the metric defined by ${\widetilde{g}} \big ((\xi _1, z_1), (\xi _2,z_2)\big )= g(\xi _1, \xi _2) + z_1z_2$, and where $\widetilde{\textrm{div}}$ and ${{\widetilde{\nabla }}}$ denote the divergence and Riemannian gradient with respect to this product metric respectively.

6 Which equals $\sigma _{n,s} \frac{z^s}{(|x-y|^2+z^2)^{\frac{n+s}{2}}}$ on ${\mathbb {R}}^n$ with its standard metric, for some normalization constant $\sigma _{n,s}>0$.
